# Epigenetic Regulation of *Verticillium dahliae* Virulence: Does DNA Methylation Level Play A Role?

**DOI:** 10.3390/ijms21155197

**Published:** 2020-07-22

**Authors:** Jorge A. Ramírez-Tejero, Carmen Gómez-Lama Cabanás, Antonio Valverde-Corredor, Jesús Mercado-Blanco, Francisco Luque

**Affiliations:** 1 Center for Advanced Studies in Olive Grove and Olive Oils, Department of Experimental Biology, Univ. Jaén, 23071 Jaén, Spain; jrtejero@ujaen.es; 2 Department of Crop Protection, Institute for Sustainable Agriculture, Agencia Estatal Consejo Superior de Investigaciones Científicas (CSIC), Campus ‘Alameda del Obispo’, Avenida Menéndez Pidal s/n, 14004 Apartado, Córdoba, Spain; cgomezlama@ias.csic.es (C.G.-L.C.); valverde@ias.csic.es (A.V.-C.); jesus.mercado@ias.csic.es (J.M.-B.)

**Keywords:** *Verticillium dahliae*, DNA methylation, defoliating pathotype, epigenetics, genomics, olive, virulence

## Abstract

*Verticillium dahliae* is the etiological agent of Verticillium wilt of olive. The virulence of Defoliating *V. dahliae* isolates usually displays differences and high plasticity. This work studied whether an epigenetic mechanism was involved in this plasticity. An inverse correlation between virulence and DNA methylation of protein-coding genes was found. A set of 831 genes was selected for their highly consistent inverse methylation profile and virulence in the five studied isolates. Of these genes, ATP-synthesis was highly represented, which indicates that the more virulent D isolates are, the more energy requirements they may have. Furthermore, there were numerous genes in the protein biosynthesis process: genes coding for the chromatin structure, which suggests that epigenetic changes may also affect chromatin condensation; many transmembrane transporter genes, which is consistent with denser compounds, traffic through membranes in more virulent isolates; a fucose-specific lectin that may play a role in the attachment to plant cell walls during the host infection process; and pathogenic cutinases that facilitate plant invasion and sporulation genes for rapid spreading alongside plants. Our findings support the notion that differences in the virulence of the Defoliating *V. dahliae* isolates may be controlled, at least to some extent, by an epigenetic mechanism.

## 1. Introduction

*Verticillium dahliae* Kleb. is a soil-borne hemibiotrophic phytopathogenic fungus that causes vascular diseases (Verticillium wilts) in hundreds of plant species worldwide, including high-value herbaceous and woody crops [1,2]. Verticillium wilts are extremely difficult to control because of factors like the broad host range displayed by the pathogen, its ability to produce microsclerotia (resting structures able to endure for long time periods in soil and infected plant residue), or the systemic nature of infections [3].

*Verticillium dahliae* causes serious problems in many areas where olive (*Olea europaea* L. subsp. *europaea* var. *europaea*) is cultivated. Indeed, Verticillium wilt of olive (VWO) is one of the most important diseases to affect this tree crop, particularly in the Mediterranean Basin where olive cultivation is of outstanding economic, social, and historical significance [4]. To date, available control measures have proven unsuccessful when applied as individual strategies. Therefore, in order to effectively control VWO, the implementation of an integrated disease management framework is highly recommendable [5]. 

*Verticillium dahliae* isolates that infect olive and cotton (*Gossypium hirsutum* L.) are typically classified as highly-virulent, defoliating (D, lineage 1A) [6] and mildly-virulent, non-defoliating (ND) pathotypes. Currently, there is much concern in many olive-cultivating areas about the alarming spread of the D pathotype [7,8,9]. D isolates produce severe defoliation in infected olive trees, whereas ND isolates usually behave as moderately virulent by causing symptoms, of which heavy defoliation of green leaves is not usually observed [10,11]. Nevertheless, the boundaries between the wilting syndromes caused by D and ND isolates are sometimes fuzzy, and a ‘continuum of virulence’ has been reported, even among the D isolates that infect olive cultivars and display a differential susceptibility level to VWO [7]. Moreover, the D isolates that originate from cotton or olive have been shown to display differential virulence in distinct accessions of *Arabidopsis thaliana* [12].

Comparative genomics is a crucial tool to unravel differences/similarities between species, and between isolates/strains of the same species. It enables the sequence differences that are potentially responsible for phenotype changes among strains of a given pathogen to be identified [13,14]. For instance, with a comparative population genomics/transcriptome sequencing combined approach, the Ave1 effector molecule secreted by isolates of *V. dahliae* race 1, which are recognized by the Ve1 immune receptor of resistant tomato plants, has been identified [14,15]. In this case, the genomes of race 1 isolates were compared to the genomes of race 2 isolates that evade recognition. Comparative genomics has also been valuable for uncovering events of horizontal gene transfer from fungi and bacteria to *V. dahliae*, which seems crucial for evolution and the host adaptation of this asexual plant pathogen species [16,17]. A comparison of the whole-genome sequences of the *V. dahliae* D and ND isolates has also enabled D pathotype-specific genomic regions to be identified, which have helped to design improved PCR-based diagnosis markers [18]. Finally, a comparative population genomics strategy, combined with gene deletion and complementation, gene expression analysis, and the quantification of *V. dahliae* secondary metabolites, has been recently implemented to identify at least one of the mechanisms involved in the defoliating phenotype [19]. 

Recent studies have shown that the genomes of *V. dahliae* isolates/strains display plasticity (i.e., extensive intra- and interchromosomal rearrangements) that explain the appearance of the unique lineage-specific regions involved in aggressiveness [15,20,21]. As part of this plasticity, the involvement of epigenetic mechanisms can be hypothesized. Epigenetic changes can produce heritable modifications without affecting the DNA sequence. Unfortunately, studies about the role of epigenetics to determine the virulence of fungal plant pathogens are lacking [22]. Epigenetic regulation has been extensively studied in yeasts and the filamentous fungus *Neurospora crassa* [23,24,25]. DNA methylation and histone modifications are the mechanisms of epigenetic changes in these fungi. An epigenetic control of effectors has been proposed for plant pathogenic fungi, and avirulence (Avr) factors would be switched on or off by epigenetic regulation [26]. It has been demonstrated that milRNA (VdmilR1) mediates the repression of a virulence gene (*VdHy1*) in *V. dahliae* [27]. This mechanism of epigenetic regulation may enhance the ability of the pathogen to adapt to different host immune systems. For this reason, the main objective of this work was to test the hypothesis that epigenetic changes may be relevant for determining the virulence of D *V. dahliae* isolates. We focused on the DNA methylation level and searched for differentially methylated genes in the D isolates displaying a distinct level of virulence. 

## 2. Results

### 2.1. Verticillium Dahliae D Isolates Show A ‘Continuum of Virulence’ in Olive

The *V. dahliae*-olive bioassay confirmed that all the tested isolates were pathogenic in ‘Picual’ plants, and their virulence ranged from highly to mildly virulent. The control (non-inoculated) plants did not develop any symptoms over time. The first symptoms (chlorosis, pale green leaves, inward rolling of leaves, and defoliation) in the *V. dahliae*-inoculated plants appeared 23 (V937I and V937I-HP), 27 (V138I and V150I), and 31 (V403I) days after inoculation (dai) with the pathogen, and some inoculated plants died 6 weeks after inoculation. The number of dead plants varied depending on the virulence of the isolate (from 2 for V138I to 12 for the V937I-HP). The ANOVA analysis of parameters S (severity) and area under the disease progress curve (AUDPC) showed significant (*p* < 0.05) differences among the tested isolates. These parameters correlated with Disease incidence (DI), Disease intensity index (DII), and mortality (M) data (Table 1). Overall, isolate V937I-HP was significantly more virulent than the other tested isolates, except for V-150I, which, however, produced lower mortality than isolate V937I-HP. The isolates from cotton (V138I) and artichoke (V403I) displayed the least virulence level in olive plants, as well as the lowest mortality rate (Table 1). Both parameters did not differ significantly from isolate V937I. According to all the analyzed disease parameters, the *V. dahliae* D isolates herein evaluated can be ordered from the most to the least virulent as follows: V937I-HP, V150I, V937I, V403I, and V138I. 

### 2.2. Assembly and Annotation of the V. dahliae D Genomes

More than 20 M Illumina paired-end (PE) reads were sequenced for each D isolate and used to assemble their genomes (Table 2). The first attempted approach was to map the PE reads to the “*Verticillium_dahliae.ASM15067v2.dna.toplevel.fa*”, available at ftp://ftp.ensemblgenomes.org/pub/fungi/release-38/fasta/verticillium_dahliae/dna/, as the reference genome. This alignment was performed using the BWA (version 0.7.17) software. However, the calling of single nucleotide variants (SNV) and indel variants with the Genome Analysis Toolkit (GATK) HaplotypeCaller (HC) in the Genomic Variant Call Format (GVCF) mode in the normal mode or with Lumpy produced inconsistent results. In fact, most of the variants labeled as unique to some isolates by the analysis pipeline were false-positives. So the *V. dahliae ASM15067v2* genome was not useful as a reference genome for assembling the *V. dahliae* D genomes of this study. For this reason, we decided to perform our own assembly using isolate V937I as the reference genome. An exhaustive evaluation of the assembly quality was made with QUAST software. The genome contained 5787 scaffolds, of which 2384 were ≥500 bp, for a total genome length of 34980341, 1 Mbp longer than the *V. dahliae ASM15067v2* genome. The largest scaffold was 208496 bp, N50 was 40727 bp, and L50 was 260 bp long. The number of Ns per 100 kbp was as low as 3.68 bp. The structural annotation of the V937I genome comprised 9170 protein-coding genes, of which 5793 were functionally annotated and 3377 remained unknown. The quality of the annotated transcriptome was analyzed by BUSCO (Table 3). Most BUSCOs were complete and single copy (e.g., 3085 / 3215), which indicated a good quality transcriptome of the V937I genome.

### 2.3. Type and Level of DNA Methylation in the V. dahliae D Genomes

**Figure 1:**
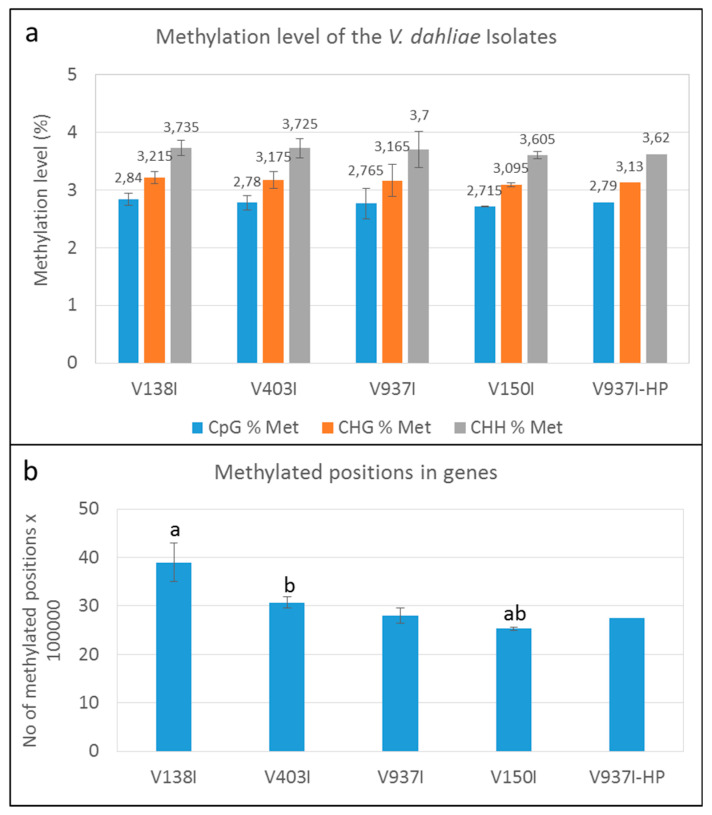
DNA methylation level in the *Verticillium dahliae* defoliating isolates. (**a**) Methylation level in the genome (%) of the *V. dahliae* isolates in the CpG, CHG, and CHH sequence contexts. (**b**) Methylated cytosines in the genes of the four non parented *V. dahliae* isolates. “a” or “b” above the bars indicate that those data significantly differed (*p* < 0.05). Only one datum of the V937I-HP biological replicate sequenced by bisulfite treatment is represented.

The methylation level was analyzed in the PE reads obtained after bisulfite treatment and mapped in each case to their own genome using BISMARK v.0.21.0. The average number of reads per isolate, having removed the duplicated reads, is presented in Table 2. The mapping efficiency with its own genome for each sample was around 42.33 ± 2.08%, except for V937I-HP sample B (mapping efficiency < 30%), which was considered an outlier and was removed from the analysis (Figure S1). The average percentages of the methylated cytosines in isolate V937I were 2.765% in the CpG, 3.165% in the CHG and 3.7% in the CHH sequence contexts. Similar percentages were found in the other isolates (Figure 1A). 

The results indicated that methylation at the whole genome level showed a minor tendency toward lower methylation in those isolates displaying higher virulence. Nevertheless, this trend was almost negligible and insignificant. Remarkably, however, a clear trend in which methylation inversely correlated with virulence of the D isolates was observed when only the gene coding sequences were analyzed (Figure 1B). 

### 2.4. The Genes of the Highly Virulent V937I-HP Isolate are, on Average, Less Methylated than in Parental Isolate V937I

**Figure 2:**
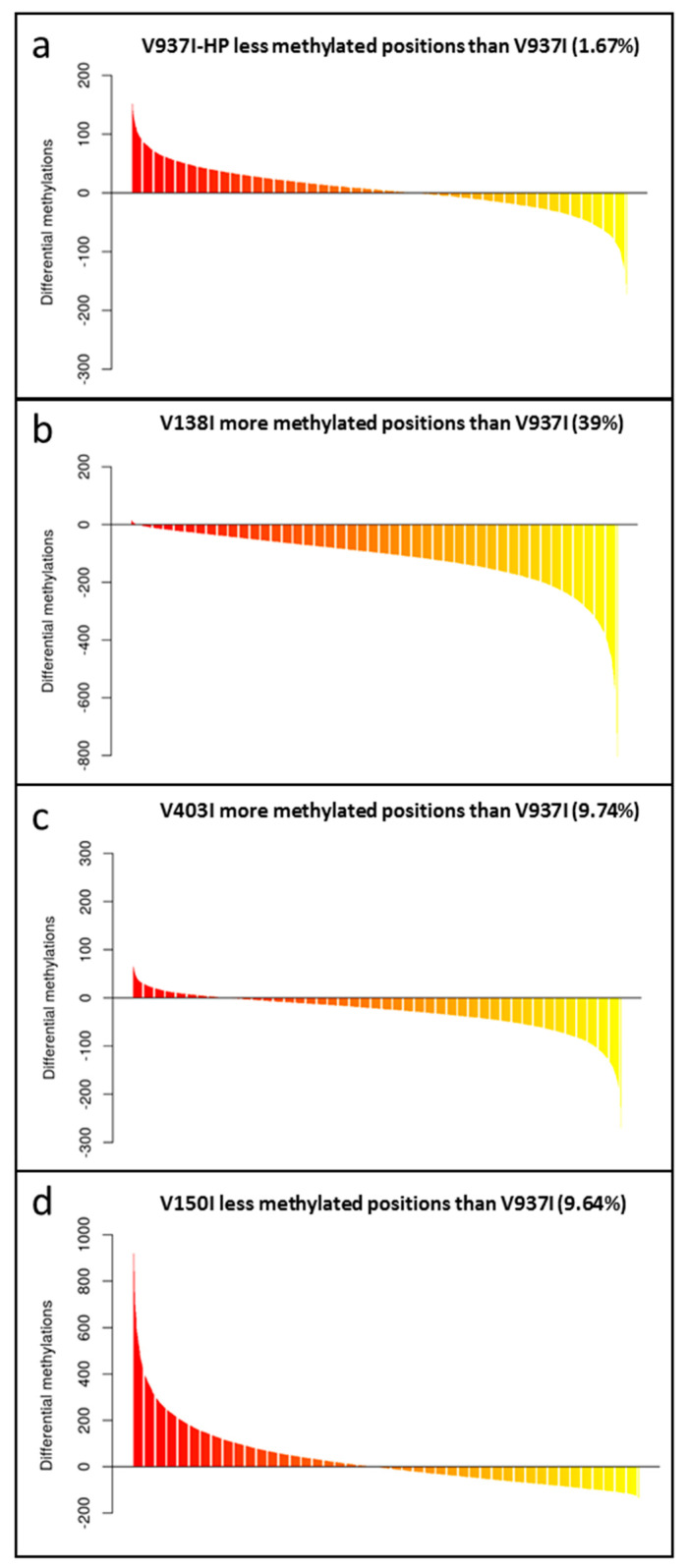
The gene methylation pattern of the *Verticillium dahliae* isolates compared to the reference V937I isolate. The average methylation % differences of the protein-coding gene sequences compared to reference isolate V937I are represented from the more positive to more negative values. Isolate V397I-HP is represented in (**a**), V138I in (**b**), V403I in (**c**) and V150I in (**d**). Data correspond to two bisulfite-treated sequenced DNA samples, except for the V397I-HP derived isolate, for which only one sample was available.

To test the role of DNA methylation in determining the virulence of the D *V. dahliae* isolates, the methylation level of the genes in V397I and its more virulent derivative V397I-HP were first compared. The analysis showed different gene methylation patterns for each isolate. While some genes were less methylated in the more virulent isolate V937I-HP, other genes did the exact opposite compared to parental isolate V973I. On average, isolate V937I-HP showed 1.67% less methylation than that observed in isolate V937I when all the gene sequences were analyzed on the whole (Figure 2a. In addition, 3145 genes were found to be at least 1% less methylated in isolate V937I-HP than in isolate V937I, and the gene with the biggest difference in the methylation level was as high as 67.11%. Considering the close genetic relation between both isolates, it is tentative to hypothesize that the differential methylation level could be involved in determining the big differences found in virulence between them.

### 2.5. The Methylation Level of Many Genes Inversely Correlates with D Isolate Virulence

The finding that overall lower gene methylation could be associated with the higher virulence of the isolates was supported by the analysis of the general methylation level of four of the non-isogenic D isolates obtained from different hosts and/or geographical origins (Figure 1B). In fact, the degree of virulence in olive and the gene methylation level showed opposite trends. The more virulent isolate V150I was significantly less methylated than the less virulent isolates V138I and V403I (Figure 1B). 

**Figure 3:**
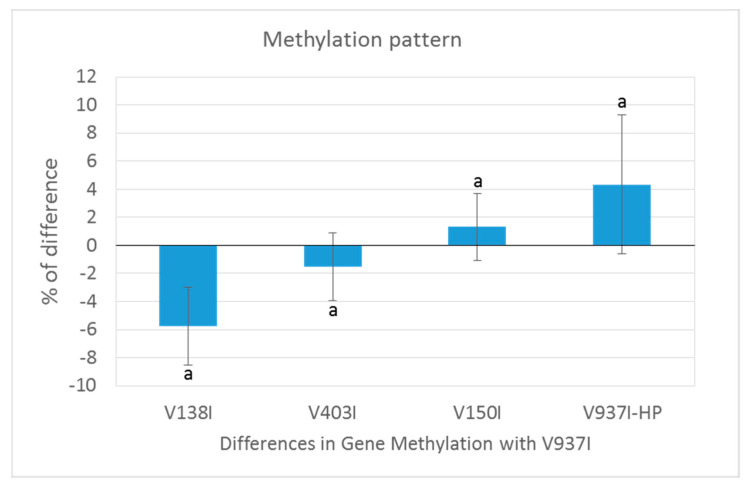
Methylation pattern of the genes selected as being less methylated in the isogenic and more virulent V937I-HP than its parental isolate V937I. The 3145 genes that were at least 1% less methylated in V937I-HP than in the isogenic and less virulent V937I are represented. Bars indicate the average difference of methylation in each isolate compared to reference isolate V937I. The *Verticillium dahliae* D isolates are shown from the least (left) to the most (right) virulent. ‘a’ means that all the comparisons were significant (*p* < 0.001).

**Figure 4:**
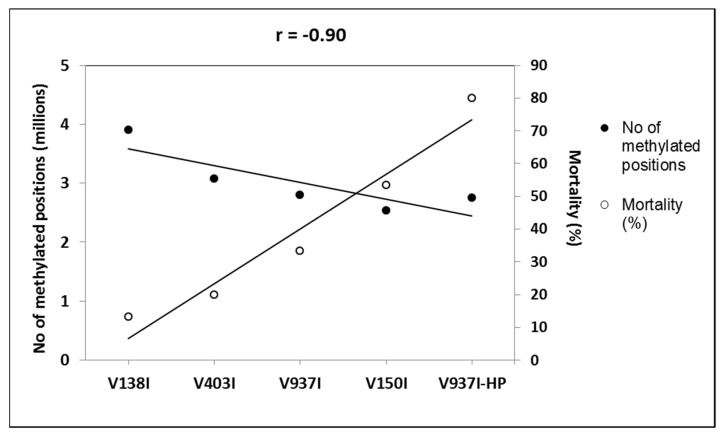
Negative correlation between virulence and DNA methylation of the protein-coding genes. The number of the total methylation positions in the protein-coding genes and the mortality induced data are represented. The isolates are placed from left to right, and they are ordered by increasing virulence.

The gene methylation level between isolate V937I and all the other D isolates was compared. The results showed that the less virulent isolates (V138I and V403I) had higher methylation levels than that of V937I (39% and 9.7%, respectively), whereas the more virulent isolate (V150I) displayed less methylated genes (9.6%) (Figure 2b–d). In addition, the 3145 genes that were at least 1% less methylated in V937I-HP than in V937I were also less methylated in the highly-virulent isolate V150I than in V937I and were more methylated in the less virulent V138I and V403I than in V937I. The general differential methylation pattern of these genes is represented in Figure 3. A strong negative correlation of virulence and differential methylation in the protein-coding genes was observed r = −0.90 (*p* < 0.1) (Figure 4). In addition, 831 genes fulfilled the criteria of being at least 1% more methylated in the less virulent isolates V138I and V403I than in isolate V937I and were 1% less methylated in the more virulent ones (V150I and V937I-HP). This subgroup of genes showed how the methylation pattern inversely correlated with the virulence of the isolates, and it was selected for further analyses (Table S1). 

### 2.6. Analysis of Less Methylated Genes in the D Isolates Showing Higher Virulence in Olive

**Figure 5:**
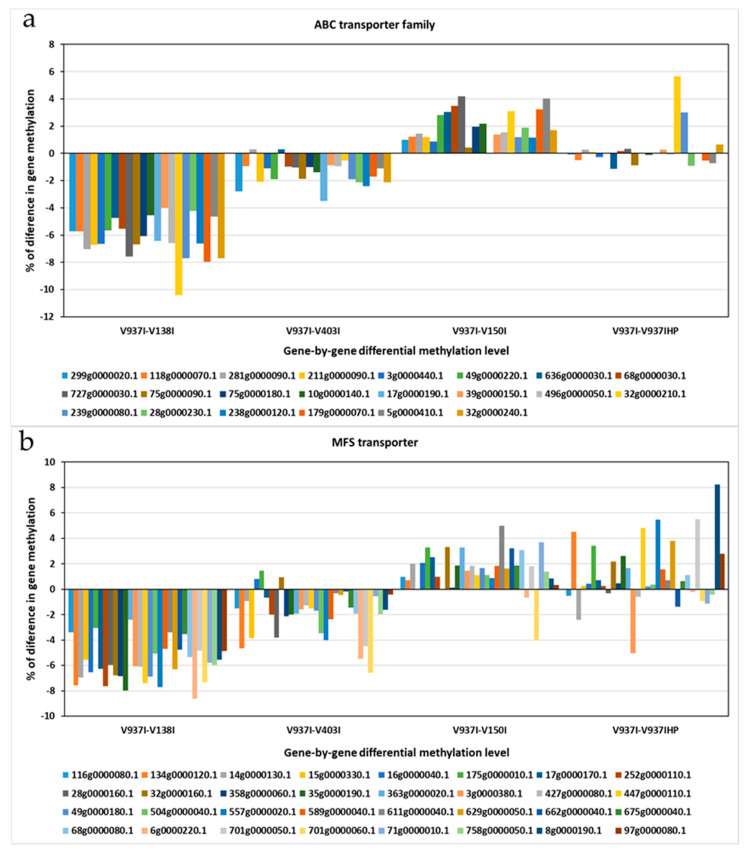
The differential methylation profile of the annotated genes for the two major transporter superfamilies. The gene methylation pattern of the *Verticillium dahliae* D isolates compared to reference isolate V937I is represented for each gene. (**a**) ATP-Binding Cassette (ABC) transporter superfamily. (**b**) Major Facilitator Superfamily (MFS) transporters superfamily. The methylation differences between isolates for each gene was calculated from average data in the biological replicates. Gen ID can be consulted in Table S2.

The 831 previously selected genes covered a wide range of Gene Ontology (GO) term Biological Processes (BP), where oxidation-reduction (2.91%), transmembrane transport (1.99%), translation (1.77%), ion transport (1.18%), and regulation of transcription (1.18%) processes were the most represented, followed by other BP involved in general cellular processes, like cell cycle (0.81%), methylation (0.51%), or pathogenesis (0.40%) (Figure S2). The Molecular Function (MF) also revealed a wide range of MF present among the 831 differentially methylated genes, which indicates that many BP and MF are affected by the differential methylation of these genes (Figure S2). The most represented MF were metal ion binding (6.56%), nucleotide binding (4.89%), transferase activity (4.24%), and ATP binding (3.95%). Other highly represented MF terms were oxidoreductase activity (2.97%), DNA binding (2.89%), and protein binding (2.40%). The Cellular Component (CC) was found to be extremely well represented. The most represented were the cytoplasm (10.67%), membrane (10.62%), and nucleus (8.93%), with a high proportion of integral components of membranes (7.95%) and plasma membranes (5.91%) (Figure S2). In fact, this subset of genes included one of the ATP-Binding Cassette (ABC) primary transporter superfamily transporters and six of the Major Facilitator Superfamily (MFS) transporters. The finding that transmembrane transporter genes were less methylated in the more virulent D isolates was a general tendency for all the annotated transporters genes for both gene families ABC and MFS (Figure 5). 

**Figure 6:**
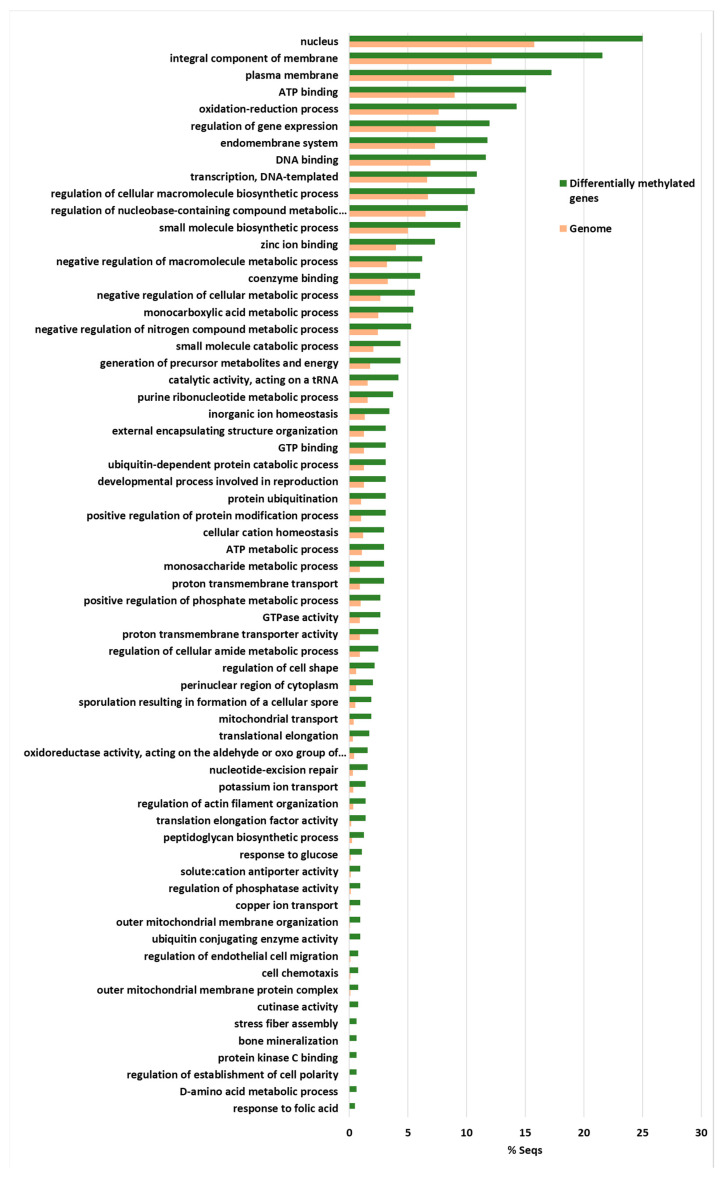
The Fischer exact test-enriched GO terms of genes displaying an inverse pattern of virulence and DNA methylation in the *Verticillium dahliae* D isolates. The 831 genes that fulfilled the criteria of being at least 1% more methylated in V937I that in the more virulent V937I-HP and V150I isolates, and 1% less methylated in V937I than in the less virulent V403I and V138I, are represented. The methylation differences between isolates for each gene was calculated from average data in the biological replicates.

The highest enriched GO terms were the nucleus, and gene expression regulation and DNA templated transcription and DNA binding were also highly enriched terms (Figure 6). 

**Figure 7:**
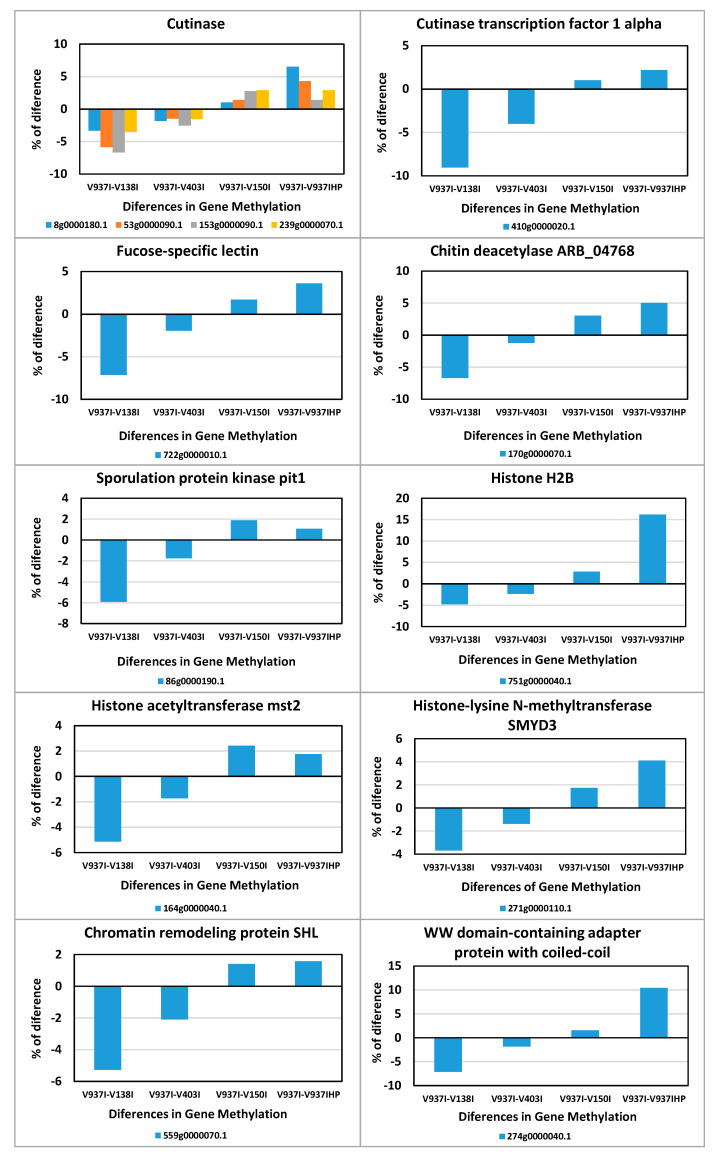
Differences in methylation of some of the genes involved in pathogenicity, sporulation, or chromatin condensation. The differential methylation profiles of some of the 831 selected genes involved in relevant processes are represented.

This indicates that gene expression regulation was over-represented among these 831 genes. In addition, the integral components of membranes, plasma membranes and the endomembrane system were also highly enriched, as were ATP binding and the oxidation-reduction process (Figure 6). Thus, many of the genes involved in ATP production and protein biosynthesis were represented in this group of differentially methylated genes. This group included four Cutinase-coding genes, a Cutinase Transcription Factor 1 alpha gene (*CTF1-ALPHA*) involved in pathogenesis, as well as a Fucose-specific lectin gene potentially involved in cell-cell interactions and parasitic relations [28] (Figure 7). A Chitin deacetylase ARB_04768 gene was also present and plays a potential role in fungal hyphae penetration into plants by modifying chitin, which can be recognized by a plant resistance system [29]. Additionally, chitin deacetylases can be involved in the sporulation process and fruiting body development [30,31] (Figure 7). A gene essential for sporulation, such as *pit1*, which codes for a protein kinase, has also been included in the 831 differentially methylated genes [32]. Several genes affecting chromatin structure and transcription activation are also differentially methylated. Thus, the genes coding for histone H2B, histone acetyltransferase, histone-lysine N-methyltransferase, chromatin remodeling protein SHL, and the WW domain-containing adapter protein with coiled-coil are among them (Figure 7).

## 3. Discussion

Virulence of plant pathogenic fungi has been studied, based mainly on gene mutations, chromosome rearrangements and gene expression profile. Thus, with *V. dahliae*, specific genomic regions have been involved in aggressiveness [15,20,21]. Several genes have been associated with virulence based on transcriptomic analyses [4,33]. Epigenetics provides heritable genome changes without affecting DNA sequences. As epigenetic changes can be reverted, they may provide fungi with a higher plasticity mechanism than gene mutations or chromosomal rearrangements. However, studies that have analyzed the role of epigenetic changes in the virulence of plant pathogenic fungi in general [22], and in *V. dahliae* in particular, are lacking. 

In this work, two strategies were followed to study the putative role of DNA methylation in determining the virulence of the *V. dahliae* D isolates. On the one hand, whether the DNA methylation level correlated with the virulence level of four D isolates was evaluated. To do so, the DNA of the four isolates was sequenced, and one of them (V937I) was used to assemble the *V. dahliae* D genome. This genome was used as a reference genome to assemble the other three D genomes (V138I, V403I and V150I). B bisulfite sequencing was also performed to determine the methylated positions in the four genomes. On the other hand, a highly-aggressive derivative of isolate V937I was obtained. This new isolate (V397I-HP) was then sequenced with bisulfite treatment and compared with the reference isolate (parental in this case) V937I. 

A first analysis of the DNA methylation in *V. dahliae* done in our reference genome (V937I) showed that the CHH sequence context was more methylated (3.7%) than the CHG (3.16%) and CpG (2.76%) sequence contexts (Figure 1A). This low level of DNA methylation found in *V. dahliae* is in concordance with most fungi, for instance, in *Aspergilli* species, and about 0.25% of cytosines are methylated [34]. However, low levels in DNA methylation may play a role in gene regulation. In this sense, induced methylation inhibition led to morphological and virulence changes [35,36]. While the methylation level and aggressiveness were inversely correlated (i.e., the greater the virulence, the lower the methylation level; Table 1 and Figure 1A), the differences observed in methylation among D isolates were negligible and not statistically significant. However, it was only when coding sequences were considered, instead of the whole genome, did differences become clearly significant (Figure 1B). This indicates that small changes in the genome DNA methylation level can suffice to account for marked epigenetic changes to some or many protein-coding genes. Therefore, in order to find the genes whose methylation levels could be relevant for the virulence of the D isolates, we searched for the consistently less methylated genes in the more virulent isolates. 

Isogenic isolates V937I and V397I-HP were first compared, and 3145 genes were found to be less methylated in highly aggressive isolate V937I-HP. These genes presented an inverse pattern of methylation and virulence when considering the five isolates, which led to a very negative correlation (r = −0.90) (Figure 4). A further selection was made by taking into account only those genes that were at least 1% more methylated in less virulent isolates V138I and V403I than in reference isolate V937I, and were 1% less methylated than more virulent isolates V150I and V937I-HP. By adopting this criterion, a set of 831 genes was obtained based on their consistent methylation level, which was inverse to the virulence of the five *V. dahliae* isolates, measured as induced plant mortality.

Such a large number of genes involved many of, if not all, the main BP and MF. For this reason, analyzing and discussing all processes are unpractical, but it is worth highlighting the following processes. ATP-synthesis was well represented among the selected genes, which indicates that more virulent isolates could have higher energy requirements. The genes involved in protein biosynthesis were also highly represented. The fact that several genes coding for chromatin structure (e.g., histone H2B, histone acetyltransferase, histone-lysine N-methyltransferase, chromatin remodeling protein SHL, and WW domain-containing adapter protein with coiled-coil) were found in this subset of genes (Figure 7) suggests that epigenetic changes might not be due only to DNA methylation, but also to chromatin condensation. These results indicate that more virulent D isolates could have less condensed chromatin in less methylated gene sequences. 

Many membrane protein-coding genes were also represented in the 831 differentially-methylated genes subset. Of these, transport protein-coding genes were well represented. In fact, when analyzing all the ABC and MFS transporter gene families, including many of the selected 831 genes, an overall tendency toward less methylation in the more aggressive isolates was found (Figure 5). This suggests denser compounds traffic through membranes in more virulent isolates. A membrane integral protein fucose-specific lectin was also one of the selected genes (Figure 7). Fucosylated glycans are present in plant cell walls and can act as targets for bacterial receptors [37]. In pathogenic fungi, such as the genus *Aspergillus*, lectins are involved in cell surface attachment and host-pathogen interactions during infection, and they contribute to its pathogenicity [38,39]. Similarly, the fucose-specific lectin protein of *V. dahliae* may employ the plant cell wall as a target by playing a role in the attachment to it during host infection. 

Two genes presumably involved in sporulation were also less methylated in the more virulent D isolates. On the one hand, *pit1* is essential for sporulation but not for meiosis in *Schizosaccharomyces pombe* [32]. On the other hand, a chitin deacetylase gene may be involved in the sporulation process [30,31] and might, alternatively, play a role in fungal hyphae penetration into plants [40] (Figure 7). Spore formation may facilitate the dissemination of fungi inside plants, where they may move through the vascular system and seed distant places. In addition to the chitin deacetylase gene, four cutinase-coding genes and a *cutinase transcription factor 1 alpha* gene (*CTF1-ALPHA*) fulfilled the criterion adopted to generate the subset of 831 genes (Figure 7). Cutinases are involved specifically in plant infection by fungi and its specific inhibition blocked infectivity in several pathogen/host systems [41]. While some cutinases are essential for pathogenesis, others are expressed during saprophytic growth on cutin as a carbon source [42,43]. In this case, the differential DNA methylation profile suggests that these five genes may be involved in *V. dahliae* infection of plants.

In conclusion, the genes involved in epigenetic modification, transmembrane traffic and plant-pathogen interaction, and the genes essential for pathogenesis, were found to be differentially methylated, a process that correlated inversely with the virulence of *V. dahliae* D isolates herein analyzed. Our findings support the hypothesis that differences in the virulence of D isolates may be controlled, at least to some extent, by an epigenetic mechanism.

## 4. Methods

### 4.1. Verticillium Dahliae Isolates

Five *V. dahliae* isolates, all representative of the highly-virulent, defoliating (D) pathotype (linage 1A) [6], were herein used. Isolates V150I [44] and V937I [33] originated from diseased olive trees, isolate V138I was obtained from an infected cotton plant [45], and isolate V403I originated from a wilted artichoke plant [46]. All these isolates were molecularly characterized by different approaches, and information about them is found in the above-cited studies. Moreover, their athogenicity and virulence in different hosts have been consistently reported and assessed [7,12,46]. Finally, isolate V937I-HP was obtained from the stems of diseased olive (cv. Picual) plants after two consecutive rounds of artificial inoculation with isolate V937I. Briefly, after the first infection round, a single-spore isolate was obtained that was used for the second round. Then, a new single-spore isolate was obtained and used in this study. Isolate V937I-HP shows the typical PCR A pattern [47] associated with D isolates, similarly to that displayed by isolate V937I. The previous routine experiments using ‘Picual’ plants revealed that V937I-HP was consistently more virulent than parental isolate V937I. All the isolates are kept in the fungal culture collection of the Department of Crop Protection, Institute for Sustainable Agriculture (CSIC), Córdoba, Spain. 

### 4.2. Verticillium Dahliae-Olive Bioassay

A bioassay to re-assess the degree of virulence of the *V. dahliae* isolates used in this study was carried out under non gnotobiotic conditions. Olive plants (cv. Picual, 4 months old) from a commercial nursery from the province of Córdoba province were acclimated for 3 weeks in a greenhouse under the conditions described below. One day before the inoculation with the *V. dahliae* isolates, plants were carefully uprooted from the original substrate and transplanted in new polypropylene pots (9 × 9 × 11 cm, one plant per pot) containing fresh potting substrate without causing intentional wounding to roots. Pots were randomly distributed in three blocks (15 plants per treatment) under natural lighting and at day/night temperatures of 27/21 °C. Plants were inoculated by adding to each pot 150 mL of a conidia suspension (1 × 10^6^ conidia/mL) of each isolate, prepared as previously described [48]. The non-inoculated (control) plants were watered with the same volume of water. Disease development was assessed by scoring symptom severity (S) on a scale from 0 to 4 according to the percentage of affected leaves and twigs (0, no symptoms; 1, 1–33%; 2, 34–66%; 3, 67–100%; 4, dead plant) [49] twice weekly for 84 days after pathogen inoculation. The area under the disease progress curve (AUDPC) was calculated, and an analysis of variance (ANOVA) for this parameter was carried out [50]. The mean values were compared by Fisher’s protected protected least significant difference (LSD) at *p* = 0.05 using the Statistix program (Version 10.0 for Windows Analytical software 1985–2013). Other parameters, such as Disease incidence (DI), Mortality (M), and Disease intensity index (DII), were also calculated for each treatment.

### 4.3. DNA Extraction

The *Verticillium dahliae* isolates were grown in potato-dextrose-broth (PDB, Difco, Detroit, MI, USA) medium in an orbital shaker (165 rpm) at 25 °C for 7days. Then, mycelia were filtered using sterile gauzes, lyophilized, and ground to a fine powder using MM 301 mixer mills (Retsch GmbH, Haan, Germany). The conidia suspensions obtained after filtering were used to inoculate olive plants (see below). Therefore, the fungal biomasses grown in each culture batch were used for: (i) DNA extraction and purification (mycelia, for the genome sequencing and DNA methylation analysis); (ii) assessment of the virulence level of each D isolate (conidia). DNA was extracted from 50 mg ground mycelium by the procedure described in Reference [51] and purified using the DNA Clean & Concentrator Kit (Zymo Research, Irvine, CA, USA) according to the manufacturer’s instructions. The purity and quality of the DNA samples were checked by electrophoresis in 0.8% (*w/v*) agarose gels stained with RedSafe™ nucleic acid staining solution (iNtRON Biotechnology, Inc., Burlington, MA 01803, USA) and visualized under UV light, as well as by a Qubit 2.0 fluorometer (Invitrogen, Waltham, MA, USA). 

### 4.4. Genome Sequencing, Assembly and DNA Methylation Analysis 

The genomic DNA from the five D *V. dahliae* isolates was paired-end (PE) sequenced (2 × 150 bp) at the qGenomics S.L. Sequencing NGS Service (Esplugues de Llobregat, Barcelona, Spain) on an Illumina NextSeq 500 platform with a Mid Output Flow Cell (130 M). The number of raw sequenced reads is shown in Table 1. The depth of coverage achieved in the genomic sequencing was typically >100X. All the *V. dahliae* genomes were assembled de novo with the SPADES v3.11.1 alignment software [52] using its adapted algorithms to assemble multiple PE reads [53,54]. The quality of the assembled genomes was tested by BUSCO v1.2 [55] by applying the *sordariomyceta_obd9* as BUSCO lineage and *Fusarium graminearum* as supporter species for gene finding, as well as QUAST v3.0.2 with the default parameters. The structural annotation of the low complexity repetitive sequences was performed with RepeatModeler, RepeatMasker, and MITEHunter. For predictions of genes and transcripts, an evidence-based method was used with software Augustus, SNAP, HiSat, and StringTie. The functional annotation was made using the blastp search in the Swissprot database. Finally, for the methylation analysis, two biological replicates (A and B) of each isolate were PE-(150 bp) sequenced after bisulfite treatment at the qGenomics S.L. NGS Service. The analysis of each isolate bisulfite treated PE reads was performed with BISMARK v.0.21.0 by mapping to their own genomes [29]. The methylated positions in all the contexts were extracted from the BISMARK methylation extractor tool by focusing the analysis on two approaches: number of the methylated position across the genome; number of the methylated positions placed inside the mRNA coordinates. Finally, a GO terms analysis was conducted with the Blas2GO software [56] and by applying a Gene Enrichment Analysis based on a Fischer Exact t-test with a 1% False Discovery Rate (FDR) threshold. The lists of the differentially methylated genes among strains were used as a test set and compared against the whole genome (reference set).

## Figures and Tables

**Table 1:** Differential virulence in olive (cv. Picual) plants displayed by the *Verticillium dahliae* defoliating isolates used in this study.

	Disease Parameters				
Treatments	S	AUDPC	DI (%)	DII	M (%)
V-937I	1.65 bc	57.9 bc	53.3	0.41	33.3
V-937I-HP	3.32 a	133 a	93.3	0.83	80
V-138I	1.18 c	43.7 c	53.3	0.3	13.3
V-150I	2.57 ab	92.6 ab	73.3	0.64	53.3
V-403I	1.47 bc	42.3 c	60	0.33	20

AUDPC, area under the disease progress curve over time. S, mean of disease severity symptoms (from 0 to 4) at the end of the experiment; Final DI, final disease incidence (%); Final DII, disease intensity index (ranging 0–1) calculated with data on incidence and severity of the recorded symptoms; M, dead plants (%) at the end of the experiment. All the parameters are referred to at the end of the experiment (84 days after pathogen inoculation). Data are the average of three randomly-distributed blocks, each with five pots (plants) per treatment. The non-inoculated (control) plants presented no disease symptoms and were not included in the statistical analysis. The means in each column followed by different letters are significantly different according to Fisher’s protected least significant difference (LSD) test (*p* = 0.05).

**Table 2:** Sequencing data of the *Verticillium dahliae* defoliating isolates.

Isolate DNA	Without Bisulfite Treatment (No Reads)	With Bisulfite Treatment (No Reads in Two Replicates)	
V-937I	29,352,982	18,662,110	10,547,437
V-937I-HP	24,087,863	16,318,447	11,068,341
V-138I	22,157,160	16,817,943	22,237,529
V-150I	21,766,421	12,384,988	14,527,876
V-403I	20,520,224	15,113,143	13,553,206

**Table 3:** Transcriptome quality.

Summarized Benchmarks in the BUSCO Notation C: 86%(D:3.4%),F: 6.1%,M:7.5%,n: 3725	
Complete and single-copy BUSCOs	3085
Complete BUSCOs	3215
Complete and duplicated BUSCOs	130
Fragmented BUSCOs	229
Missing BUSCOs	281
Total BUSCO groups searched	3725

BUSCO was run in the genome mode.

## Data Availability

The raw NGS sequence data set supporting the results of this study and the assembled genomes are deposited in the National Center for Biotechnology Information (NCBI) Sequence Read Archive (SRA) and are available under Project Number PRJNA599551.

## Supplementary Materials

The following are available online at https://www.mdpi.com/1422-0067/21/15/5197/s1, Figure S1: Mapping efficiency of bisulfite treated PE reads with its own genome, Figure S2: GO term analysis of the 831 genes selected, Table S1: List of genes that showed a methylation pattern inversely correlated to the virulence of the isolates, Table S2: ABC and MFS transporters superfamilies.
