# Supplementary material for: Epigenetic Regulation of Verticillium dahliae Virulence: Does DNA Methylation Level Play A Role?
Source: Int J Mol Sci. 2020 Jul 22;21(15):5197. doi: 10.3390/ijms21155197 (PMC7432615; doi:10.3390/ijms21155197)

Epigenetic regulation of *Verticillium dahliae* virulence: does DNA methylation level play a role?

Jorge A. Ramírez-Tejero[1], Carmen Gómez-Lama Cabanás[2], Antonio Valverde-Corredor[2], Jesús Mercado-Blanco[2] and Francisco Luque[1]*

[1]Center for Advanced Studies in Olive Grove and Olive Oils, Department of Experimental Biology, Univ. Jaén, 23071-Jaén, Spain

[2]Department of Crop Protection, Institute for Sustainable Agriculture, Agencia Estatal Consejo Superior de Investigaciones Científicas (CSIC), Campus 'Alameda del Obispo', Avenida Menéndez Pidal s/n, Apartado 14004 Córdoba, Spain

Email: jrtejero@ujaen.es; cgomezlama@ias.csic.es; valverde@ias.csic.es; jesus.mercado@ias.csic.es; fjluque@ujaen.es.

*Corresponding author F. Luque, fjluque@ujaen.es. ORCiD ID: 0000-0003-1354-3533

Table S1

937-HP_NODE_NU	GENE NAME	GENE LENG	DIFF	%	937-138_NODE_NU	GENE LE	DIFF	%	937-150_NODE_N	GENE LE	DIFF	%	937-403_NODE_N	GENE LEN	DIFF	%
100g0000040.1	Unknown protein	2903	55	1,894591802	100g0000040.1	2903	-197,5	-6,803306924	100g0000040.1	2903	41,5	1,429555632	100g0000040.1	2903	-68	-2,342404409
100g0000050.1	Unknown protein	2732	66	2,415812592	100g0000050.1	2732	-133	-4,868228404	100g0000050.1	2732	69,5	2,543923865	100g0000050.1	2732	-44,5	-1,628843338
100g0000060.1	Unknown protein	1986	27,5	1,38469285	100g0000060.1	1986	-108,5	-5,463242699	100g0000060.1	1986	36	1,812688822	100g0000060.1	1986	-27	-1,359516616
100g0000190.1	Unknown protein	569	60	10,54481547	100g0000190.1	569	-26	-4,569420035	100g0000190.1	569	24	4,217926186	100g0000190.1	569	-12,5	-2,196836555
1013g0000010.1	Unknown protein	254	5,5	2,165354331	1013g0000010.1	254	-21	-8,267716535	1013g0000010.1	254	12,5	4,921259843	1013g0000010.1	254	-32	-12,5984252
1013g0000020.1	Unknown protein	724	43,5	6,008287293	1013g0000020.1	724	-25	-3,453038674	1013g0000020.1	724	18	2,486187845	1013g0000020.1	724	-19,5	-2,693370166
1013g0000030.1	Unknown protein	906	31	3,421633554	1013g0000030.1	906	-118,5	-13,0794702	1013g0000030.1	906	10	1,103752759	1013g0000030.1	906	-15	-1,655629139
1019g0000020.1	Unknown protein	1843	69	3,743895822	1019g0000020.1	1843	-184,5	-10,01085187	1019g0000020.1	1843	35	1,899077591	1019g0000020.1	1843	-56	-3,038524145
101g0000020.1	Guanine nucleotide-binding protein subunit beta-2-like 1	2564	33,5	1,306552262	101g0000020.1	2564	-154,5	-6,02574103	101g0000020.1	2564	79	3,081123245	101g0000020.1	2564	-42	-1,638065523
101g0000080.1	Maltose permease MAL31	1913	24	1,254573968	101g0000080.1	1913	-142,5	-7,449032933	101g0000080.1	1913	21	1,097752222	101g0000080.1	1913	-38	-1,986408782
101g0000100.1	Histidine--tRNA ligase	1355	41,5	3,062730627	101g0000100.1	1355	-89	-6,568265683	101g0000100.1	1355	18	1,328413284	101g0000100.1	1355	-41	-3,025830258
1023g0000020.1	ATP-dependent zinc metalloprotease FtsH	1231	38	3,086921202	1023g0000020.1	1231	-72	-5,848903331	1023g0000020.1	1231	23,5	1,909017059	1023g0000020.1	1231	-13,5	-1,096669375
1025g0000020.1	DNA polymerase IV	1481	18	1,215395003	1025g0000020.1	1481	-36,5	-2,464550979	1025g0000020.1	1481	38	2,565833896	1025g0000020.1	1481	-19,5	-1,31667792
1028g0000030.1	Unknown protein	2792	132	4,727793696	1028g0000030.1	2792	-106,5	-3,814469914	1028g0000030.1	2792	97	3,474212034	1028g0000030.1	2792	-88	-3,151862464
1036g0000010.1	C2H2 type master regulator of conidiophore development BrlA	2304	44,5	1,931423611	1036g0000010.1	2304	-101	-4,383680556	1036g0000010.1	2304	46,5	2,018229167	1036g0000010.1	2304	-38,5	-1,671006944
1042g0000010.1	Elongation of very long chain fatty acids protein 5	2164	31	1,432532348	1042g0000010.1	2164	-160	-7,393715342	1042g0000010.1	2164	23,5	1,085951941	1042g0000010.1	2164	-40	-1,848428836
1044g0000010.1	Unknown protein	1790	60,5	3,379888268	1044g0000010.1	1790	-151,5	-8,463687151	1044g0000010.1	1790	25	1,396648045	1044g0000010.1	1790	-38,5	-2,150837989
104g0000020.1	Sulfolipid-1 exporter MmpL8	422	14	3,317535545	104g0000020.1	422	-19,5	-4,620853081	104g0000020.1	422	13	3,08056872	104g0000020.1	422	-4,5	-1,066350711
104g0000040.1	Ubiquitin carboxyl-terminal hydrolase 6	1773	20	1,128031585	104g0000040.1	1773	-104,5	-5,893965031	104g0000040.1	1773	28	1,579244219	104g0000040.1	1773	-50	-2,820078962
104g0000060.1	Epithelial splicing regulatory protein 2	2571	42,5	1,653053287	104g0000060.1	2571	-166,5	-6,476079347	104g0000060.1	2571	32,5	1,264099572	104g0000060.1	2571	-30,5	-1,186308829
104g0000100.1	Palmitoyltransferase PFA5	1725	22,5	1,304347826	104g0000100.1	1725	-87,5	-5,072463768	104g0000100.1	1725	38,5	2,231884058	104g0000100.1	1725	-18	-1,043478261
104g0000120.1	Palmitoyltransferase ZDHHC16	338	43,5	12,86982249	104g0000120.1	338	-5	-1,479289941	104g0000120.1	338	7,5	2,218934911	104g0000120.1	338	-20,5	-6,065088757
104g0000160.1	Voltage-dependent P/Q-type calcium channel subunit alpha-1A	455	29,5	6,483516484	104g0000160.1	455	-39	-8,571428571	104g0000160.1	455	5	1,098901099	104g0000160.1	455	-13	-2,857142857
1052g0000040.1	Antibiotic efflux pump periplasmic linker protein ArpA	781	65,5	8,386683739	1052g0000040.1	781	-45,5	-5,825864277	1052g0000040.1	781	13,5	1,728553137	1052g0000040.1	781	-22	-2,816901409
1052g0000060.1	Leucine-rich PPR motif-containing protein, mitochondria	1974	71,5	3,622087133	1052g0000060.1	1974	-143,5	-7,269503546	1052g0000060.1	1974	31,5	1,595744681	1052g0000060.1	1974	-40	-2,026342452
1058g0000010.1	Acetyl-coenzyme A synthetase	2231	81	3,630658897	1058g0000010.1	2231	-141	-6,320035858	1058g0000010.1	2231	28,5	1,277454057	1058g0000010.1	2231	-30	-1,344688481
106g0000050.1	Argininosuccinate synthase	1850	23,5	1,27027027	106g0000050.1	1850	-118,5	-6,405405405	106g0000050.1	1850	42,5	2,297297297	106g0000050.1	1850	-99	-5,351351351
1076g0000010.1	Carcinoembryonic antigen-related cell adhesion molecule 1	629	41,5	6,597774245	1076g0000010.1	629	-38	-6,041335453	1076g0000010.1	629	12,5	1,987281399	1076g0000010.1	629	-12	-1,907790143
107g0000050.1	Endochitinase	1565	52	3,322683706	107g0000050.1	1565	-90,5	-5,782747604	107g0000050.1	1565	30	1,916932907	107g0000050.1	1565	-70	-4,472843451
107g0000120.1	Abhydrolase domain-containing protein abhd-5.1	1706	22,5	1,31887456	107g0000120.1	1706	-109,5	-6,418522861	107g0000120.1	1706	40	2,344665885	107g0000120.1	1706	-55,5	-3,253223916
107g0000160.1	Aspartate 1-decarboxylase	869	54,5	6,271576525	107g0000160.1	869	-41	-4,718066743	107g0000160.1	869	11,5	1,323360184	107g0000160.1	869	-15,5	-1,783659379
107g0000170.1	High-affinity glucose transporter	1646	24	1,458080194	107g0000170.1	1646	-86,5	-5,255164034	107g0000170.1	1646	36,5	2,217496962	107g0000170.1	1646	-42	-2,55164034
107g0000180.1	Cytoplasmic 60S subunit biogenesis factor REI1 homolog	1116	33	2,956989247	107g0000180.1	1116	-40,5	-3,629032258	107g0000180.1	1116	54,5	4,883512545	107g0000180.1	1116	-24,5	-2,195340502
108g0000200.1	UDP-N-acetylmuramate--L-alanine ligase	1044	15,5	1,48467433	108g0000200.1	1044	-89	-8,524904215	108g0000200.1	1044	11,5	1,101532567	108g0000200.1	1044	-24,5	-2,346743295
1096g0000010.1	Serine/threonine-protein kinase SRPK	1294	21	1,622874807	1096g0000010.1	1294	-132,5	-10,23956723	1096g0000010.1	1294	29	2,241112828	1096g0000010.1	1294	-26	-2,00927357
109g0000030.1	Cytochrome c	802	25	3,117206983	109g0000030.1	802	-41,5	-5,174563591	109g0000030.1	802	19	2,369077307	109g0000030.1	802	-32	-3,990024938
10g0000040.1	1-aminocyclopropane-1-carboxylate oxidase 2	1216	40	3,289473684	10g0000040.1	1216	-73,5	-6,044407895	10g0000040.1	1216	34,5	2,837171053	10g0000040.1	1216	-33	-2,71381579
10g0000060.1	1-(5-phosphoribosyl)-5-[(5-phosphoribosylamino)methylideneamino] im	1384	55,5	4,010115607	10g0000060.1	1384	-88,5	-6,394508671	10g0000060.1	1384	31,5	2,276011561	10g0000060.1	1384	-31,5	-2,276011561
10g0000220.1	5'-3' exoribonuclease 2	4749	52,5	1,105495894	10g0000220.1	4749	-298	-6,275005264	10g0000220.1	4749	72	1,516108655	10g0000220.1	4749	-101	-2,126763529
10g0000230.1	Signal peptidase complex subunit 3	1042	26,5	2,54318618	10g0000230.1	1042	-17	-1,631477927	10g0000230.1	1042	25,5	2,447216891	10g0000230.1	1042	-11,5	-1,103646833
110g0000030.1	Unknown protein	706	13	1,841359773	110g0000030.1	706	-35	-4,957507082	110g0000030.1	706	9	1,274787535	110g0000030.1	706	-11,5	-1,628895184
110g0000170.1	Unknown protein	503	21	4,174950298	110g0000170.1	503	-24	-4,771371769	110g0000170.1	503	11,5	2,286282306	110g0000170.1	503	-8,5	-1,689860835
111g0000100.1	Unknown protein	2128	62	2,913533835	111g0000100.1	2128	-92,5	-4,346804511	111g0000100.1	2128	50	2,34962406	111g0000100.1	2128	-25	-1,17481203
111g0000150.1	Unknown protein	1052	37	3,517110266	111g0000150.1	1052	-24,5	-2,328897338	111g0000150.1	1052	21,5	2,043726236	111g0000150.1	1052	-16,5	-1,568441065
112g0000030.1	Unknown protein	630	17,5	2,777777778	112g0000030.1	630	-10,5	-1,666666667	112g0000030.1	630	38,5	6,111111111	112g0000030.1	630	-26,5	-4,206349206
112g0000050.1	Unknown protein	803	34,5	4,296388543	112g0000050.1	803	-66,5	-8,281444583	112g0000050.1	803	27	3,362391034	112g0000050.1	803	-37,5	-4,669987547
112g0000140.1	Unknown protein	366	67,5	18,44262295	112g0000140.1	366	-12	-3,278688525	112g0000140.1	366	16,5	4,508196721	112g0000140.1	366	-10,5	-2,868852459
113g0000080.1	Calcium-binding protein 39-like	2458	29	1,179820993	113g0000080.1	2458	-96	-3,905614321	113g0000080.1	2458	30,5	1,240846216	113g0000080.1	2458	-61,5	-2,502034174
116g0000140.1	Phenylalanine--tRNA ligase alpha subunit	792	32,5	4,103535354	116g0000140.1	792	-39	-4,924242424	116g0000140.1	792	10,5	1,325757576	116g0000140.1	792	-10,5	-1,325757576
116g0000150.1	Interferon-induced GTP-binding protein Mx1	880	29,5	3,352272727	116g0000150.1	880	-87	-9,886363636	116g0000150.1	880	9	1,022727273	116g0000150.1	880	-34,5	-3,920454546
117g0000090.1	E3 ubiquitin-protein ligase MARCH11	391	24	6,138107417	117g0000090.1	391	-24,5	-6,265984655	117g0000090.1	391	18	4,603580563	117g0000090.1	391	-6,5	-1,662404092
117g0000110.1	Ubiquitin carboxyl-terminal hydrolase	1450	41,5	2,862068966	117g0000110.1	1450	-63,5	-4,379310345	117g0000110.1	1450	33,5	2,310344828	117g0000110.1	1450	-50	-3,448275862
117g0000140.1	Potassium channel subfamily K member 12	1886	31,5	1,670201485	117g0000140.1	1886	-141	-7,476139979	117g0000140.1	1886	36,5	1,935312831	117g0000140.1	1886	-78,5	-4,162248144
1195g0000010.1	Transcription factor MYB80	1918	41,5	2,1637122	1195g0000010.1	1918	-87,5	-4,562043796	1195g0000010.1	1918	35,5	1,85088634	1195g0000010.1	1918	-32,5	-1,69447341
1199g0000020.1	Unknown protein	408	21,5	5,269607843	1199g0000020.1	408	-30,5	-7,475490196	1199g0000020.1	408	7,5	1,838235294	1199g0000020.1	408	-19,5	-4,779411765
1199g0000040.1	Unknown protein	862	19	2,204176334	1199g0000040.1	862	-52	-6,032482599	1199g0000040.1	862	9,5	1,102088167	1199g0000040.1	862	-32	-3,712296984
119g0000130.1	Bifunctional enzyme IspD/IspF	327	48,5	14,83180428	119g0000130.1	327	-24	-7,339449541	119g0000130.1	327	7	2,140672783	119g0000130.1	327	-9	-2,752293578
11g0000160.1	Unknown protein	488	75,5	15,47131148	11g0000160.1	488	-34,5	-7,069672131	11g0000160.1	488	10,5	2,151639344	11g0000160.1	488	-9	-1,844262295
11g0000250.1	Unknown protein	1228	12,5	1,017915309	11g0000250.1	1228	-64,5	-5,252442997	11g0000250.1	1228	21,5	1,750814332	11g0000250.1	1228	-41,5	-3,379478827
11g0000290.1	Unknown protein	890	86	9,662921348	11g0000290.1	890	-43,5	-4,887640449	11g0000290.1	890	34,5	3,876404494	11g0000290.1	890	-13	-1,460674157

120g0000090.1	UvrABC system protein B	1598	56	3,504380476	120g0000090.1	1598	-98	-6,132665832	120g0000090.1	1598	17	1,063829787	120g0000090.1	1598	-46,5	-2,909887359
121g0000060.1	Succinate/fumarate mitochondrial transporter	1195	87,5	7,322175732	121g0000060.1	1195	-77	-6,443514644	121g0000060.1	1195	18	1,506276151	121g0000060.1	1195	-22,5	-1,882845188
121g0000160.1	Histone-lysine N-methyltransferase SET9	2801	83,5	2,981078186	121g0000160.1	2801	-166,5	-5,944305605	121g0000160.1	2801	58,5	2,088539807	121g0000160.1	2801	-47,5	-1,69582292
121g0000190.1	Elongation factor 1-alpha	1136	12,5	1,100352113	121g0000190.1	1136	-83	-7,306338028	121g0000190.1	1136	12	1,056338028	121g0000190.1	1136	-40,5	-3,565140845
122g0000020.1	Unknown protein	1188	13	1,094276094	122g0000020.1	1188	-69	-5,808080808	122g0000020.1	1188	29,5	2,483164983	122g0000020.1	1188	-44,5	-3,745791246
122g0000060.1	Unknown protein	1741	34,5	1,981619759	122g0000060.1	1741	-51,5	-2,958070075	122g0000060.1	1741	50	2,871912694	122g0000060.1	1741	-27,5	-1,579551982
123g0000160.1	Metal homeostasis factor ATX1	1144	88	7,692307692	123g0000160.1	1144	-84	-7,342657343	123g0000160.1	1144	13	1,136363636	123g0000160.1	1144	-50	-4,370629371
123g0000210.1	Leucine-rich repeat-containing protein sog2	2950	38,5	1,305084746	123g0000210.1	2950	-240,5	-8,152542373	123g0000210.1	2950	57,5	1,949152542	123g0000210.1	2950	-46	-1,559322034
124g0000030.1	Valine--tRNA ligase	1257	70	5,568814638	124g0000030.1	1257	-58	-4,6141607	124g0000030.1	1257	15	1,193317422	124g0000030.1	1257	-26	-2,068416866
125g0000090.1	Unknown protein	2233	64	2,866099418	125g0000090.1	2233	-189	-8,463949843	125g0000090.1	2233	52	2,328705777	125g0000090.1	2233	-41	-1,83609494
125g0000110.1	Unknown protein	1570	55	3,503184713	125g0000110.1	1570	-99	-6,305732484	125g0000110.1	1570	33,5	2,133757962	125g0000110.1	1570	-46,5	-2,96178344
126g0000020.1	Secondary metabolism regulator LAE1	2319	42,5	1,832686503	126g0000020.1	2319	-129	-5,562742561	126g0000020.1	2319	69	2,97542044	126g0000020.1	2319	-55	-2,371711945
126g0000030.1	V-type proton ATPase subunit B	1236	37,5	3,033980583	126g0000030.1	1236	-44	-3,55987055	126g0000030.1	1236	22	1,779935275	126g0000030.1	1236	-39,5	-3,19579288
126g0000120.1	Leydig cell tumor 10 kDa protein homolog	2141	44	2,055114433	126g0000120.1	2141	-114,5	-5,347968239	126g0000120.1	2141	25,5	1,191032228	126g0000120.1	2141	-71	-3,31620738
1278g0000010.1	Unknown protein	921	36,5	3,963083605	1278g0000010.1	921	-65	-7,057546146	1278g0000010.1	921	37,5	4,071661238	1278g0000010.1	921	-78,5	-8,523344191
127g0000050.1	General transcription and DNA repair factor IIH subunit TFB5	2011	49,5	2,461461959	127g0000050.1	2011	-88,5	-4,400795624	127g0000050.1	2011	36	1,790154152	127g0000050.1	2011	-28,5	-1,417205371
127g0000060.1	Fatty acid oxidation complex subunit alpha	1022	14	1,369863014	127g0000060.1	1022	-34	-3,326810176	127g0000060.1	1022	20	1,956947162	127g0000060.1	1022	-16	-1,56555773
127g0000160.1	Kinesin-like protein KIF1C	5628	56,5	1,003909026	127g0000160.1	5628	-396	-7,036247335	127g0000160.1	5628	92,5	1,643567875	127g0000160.1	5628	-166	-2,949538024
128g0000130.1	Unknown protein	1938	43	2,21878225	128g0000130.1	1938	-122	-6,295149639	128g0000130.1	1938	49	2,528379773	128g0000130.1	1938	-26	-1,341589267
128g0000140.1	Unknown protein	1902	49,5	2,602523659	128g0000140.1	1902	-116,5	-6,125131441	128g0000140.1	1902	49,5	2,602523659	128g0000140.1	1902	-29	-1,524710831
129g0000030.1	Unknown protein	264	28	10,60606061	129g0000030.1	264	-9	-3,409090909	129g0000030.1	264	5	1,893939394	129g0000030.1	264	-14	-5,303030303
129g0000070.1	Unknown protein	1526	26	1,703800786	129g0000070.1	1526	-54,5	-3,571428571	129g0000070.1	1526	19	1,24508519	129g0000070.1	1526	-17	-1,114023591
12g0000060.1	Unknown protein	2778	53	1,907847372	12g0000060.1	2778	-203,5	-7,325413967	12g0000060.1	2778	55	1,979841613	12g0000060.1	2778	-36	-1,295896328
12g0000110.1	Unknown protein	2973	81,5	2,741338715	12g0000110.1	2973	-137	-4,608139926	12g0000110.1	2973	40,5	1,362260343	12g0000110.1	2973	-100	-3,363605785
12g0000180.1	Unknown protein	561	25	4,456327986	12g0000180.1	561	-33	-5,882352941	12g0000180.1	561	15	2,673796791	12g0000180.1	561	-9	-1,604278075
12g0000200.1	Unknown protein	322	16	4,968944099	12g0000200.1	322	-18,5	-5,745341615	12g0000200.1	322	9	2,795031056	12g0000200.1	322	-15,5	-4,813664596
12g0000210.1	Unknown protein	3555	38,5	1,082981716	12g0000210.1	3555	-176,5	-4,964838256	12g0000210.1	3555	43	1,209563994	12g0000210.1	3555	-66	-1,856540084
12g0000230.1	Unknown protein	1767	35,5	2,009054895	12g0000230.1	1767	-116	-6,564799095	12g0000230.1	1767	21	1,188455009	12g0000230.1	1767	-31	-1,754385965
12g0000300.1	Unknown protein	4114	87	2,11473019	12g0000300.1	4114	-285	-6,927564414	12g0000300.1	4114	45,5	1,105979582	12g0000300.1	4114	-108	-2,625182304
12g0000330.1	Unknown protein	1432	23	1,606145251	12g0000330.1	1432	-105,5	-7,367318436	12g0000330.1	1432	23	1,606145251	12g0000330.1	1432	-34,5	-2,409217877
130g0000010.1	Unknown protein	535	90	16,82242991	130g0000010.1	535	-15	-2,803738318	130g0000010.1	535	25,5	4,76635514	130g0000010.1	535	-5,5	-1,028037383
130g0000170.1	Unknown protein	318	39	12,26415094	130g0000170.1	318	-19,5	-6,132075472	130g0000170.1	318	10,5	3,301886793	130g0000170.1	318	-5,5	-1,729559748
131g0000090.1	NADH-cytochrome b5 reductase 1	1440	21,5	1,493055556	131g0000090.1	1440	-103,5	-7,1875	131g0000090.1	1440	24	1,666666667	131g0000090.1	1440	-19	-1,319444444
131g0000120.1	Zinc finger protein 143	711	16	2,250351617	131g0000120.1	711	-14,5	-2,039381153	131g0000120.1	711	15	2,109704641	131g0000120.1	711	-12	-1,687763713
132g0000110.1	Maintenance of telomere capping protein 1	2010	78,5	3,905472637	132g0000110.1	2010	-143,5	-7,139303483	132g0000110.1	2010	37,5	1,865671642	132g0000110.1	2010	-46,5	-2,313432836
133g0000170.1	Elongation factor 1-beta	2915	55,5	1,903945112	133g0000170.1	2915	-145	-4,974271012	133g0000170.1	2915	46	1,578044597	133g0000170.1	2915	-56	-1,92109777
134g0000100.1	Pre-mRNA-splicing ATP-dependent RNA helicase prp-28	626	25	3,993610224	134g0000100.1	626	-58,5	-9,345047923	134g0000100.1	626	12	1,916932907	134g0000100.1	626	-34,5	-5,511182109
134g0000140.1	Calcipressin-like protein	770	58	7,532467533	134g0000140.1	770	-54	-7,012987013	134g0000140.1	770	13	1,688311688	134g0000140.1	770	-15	-1,948051948
135g0000080.1	Unknown protein	916	60	6,550218341	135g0000080.1	916	-40	-4,366812227	135g0000080.1	916	31	3,384279476	135g0000080.1	916	-16,5	-1,801310044
136g0000030.1	Iron-sulfur clusters transporter ATM1, mitochondria	4440	57,5	1,295045045	136g0000030.1	4440	-292	-6,576576577	136g0000030.1	4440	92,5	2,083333333	136g0000030.1	4440	-140,5	-3,164414414
137g0000070.1	Seed linoleate 9S-lipoxygenase-2	764	19,5	2,552356021	137g0000070.1	764	-30	-3,926701571	137g0000070.1	764	30,5	3,992146597	137g0000070.1	764	-16,5	-2,159685864
138g0000080.1	Unknown protein	788	34	4,314720812	138g0000080.1	788	-75,5	-9,581218274	138g0000080.1	788	11	1,395939086	138g0000080.1	788	-10	-1,269035533
139g0000020.1	Ankyrin repeat and protein kinase domain-containing protein 1	1317	50,5	3,834472286	139g0000020.1	1317	-128,5	-9,757023538	139g0000020.1	1317	15,5	1,176917236	139g0000020.1	1317	-43	-3,264996204
139g0000040.1	1,4-alpha-glucan branching enzyme GlgB	1972	23,5	1,19168357	139g0000040.1	1972	-128,5	-6,516227181	139g0000040.1	1972	45	2,281947262	139g0000040.1	1972	-78,5	-3,980730223
139g0000060.1	Alpha-L-rhamnosidase rgxB	1373	49,5	3,605243991	139g0000060.1	1373	-94,5	-6,882738529	139g0000060.1	1373	36	2,62199563	139g0000060.1	1373	-34,5	-2,512745812
13g0000050.1	Unknown protein	1830	37,5	2,049180328	13g0000050.1	1830	-128,5	-7,021857924	13g0000050.1	1830	18,5	1,010928962	13g0000050.1	1830	-53,5	-2,923497268
1405g0000020.1	Calmodulin	768	9	1,171875	1405g0000020.1	768	-40,5	-5,2734375	1405g0000020.1	768	10	1,302083333	1405g0000020.1	768	-19	-2,473958333
141g0000040.1	DNA-directed RNA polymerase subunit beta	4489	67,5	1,503675652	141g0000040.1	4489	-257	-5,725105814	141g0000040.1	4489	57,5	1,280908888	141g0000040.1	4489	-119,5	-2,66206282
141g0000060.1	Mediator of RNA polymerase II transcription subunit 9	1187	38	3,201347936	141g0000060.1	1187	-65,5	-5,51811289	141g0000060.1	1187	23	1,937657961	141g0000060.1	1187	-17,5	-1,474304971
141g0000110.1	Trafficking protein particle complex subunit 3	1635	102,5	6,26911315	141g0000110.1	1635	-87	-5,321100917	141g0000110.1	1635	38	2,324159021	141g0000110.1	1635	-55,5	-3,394495413
141g0000140.1	Calpain	2573	31,5	1,224251846	141g0000140.1	2573	-99,5	-3,867081228	141g0000140.1	2573	86	3,342401866	141g0000140.1	2573	-26,5	-1,029926156
142g0000150.1	Unknown protein	3227	61,5	1,905794856	142g0000150.1	3227	-248,5	-7,700650759	142g0000150.1	3227	47	1,456461109	142g0000150.1	3227	-44,5	-1,378989774
142g0000180.1	Unknown protein	2345	24	1,023454158	142g0000180.1	2345	-159,5	-6,801705757	142g0000180.1	2345	38	1,620469083	142g0000180.1	2345	-40,5	-1,727078891
143g0000080.1	Serine--tRNA ligase	2268	47	2,072310406	143g0000080.1	2268	-79	-3,48324515	143g0000080.1	2268	36,5	1,609347443	143g0000080.1	2268	-46	-2,028218695
143g0000090.1	Lipoprotein-releasing system ATP-binding protein LolD	1204	32	2,657807309	143g0000090.1	1204	-76,5	-6,353820598	143g0000090.1	1204	26,5	2,200996678	143g0000090.1	1204	-39	-3,239202658
144g0000130.1	Anti-sigma F factor	215	41,5	19,30232558	144g0000130.1	215	-5	-2,325581395	144g0000130.1	215	11	5,11627907	144g0000130.1	215	-8,5	-3,953488372
144g0000170.1	Acetyltransferase ataH	1589	33,5	2,108244179	144g0000170.1	1589	-112,5	-7,079924481	144g0000170.1	1589	16,5	1,038388924	144g0000170.1	1589	-32	-2,013845186
145g0000020.1	Isochorismatase domain-containing protein 2	702	75	10,68376068	145g0000020.1	702	-66	-9,401709402	145g0000020.1	702	29	4,131054131	145g0000020.1	702	-18	-2,564102564
145g0000150.1	Beta-glucosidase 1B	2209	56,5	2,557718425	145g0000150.1	2209	-145	-6,564056134	145g0000150.1	2209	38,5	1,742870077	145g0000150.1	2209	-54,5	-2,467179719
146g0000090.1	Unknown protein	4435	48	1,082299887	146g0000090.1	4435	-220,5	-4,971815107	146g0000090.1	4435	84	1,894024803	146g0000090.1	4435	-98,5	-2,22096956
147g0000040.1	Unknown protein	3023	112,5	3,72146874	147g0000040.1	3023	-161	-5,325835263	147g0000040.1	3023	42	1,38934833	147g0000040.1	3023	-36,5	-1,207409858
147g0000050.1	Unknown protein	975	21,5	2,205128205	147g0000050.1	975	-12,5	-1,282051282	147g0000050.1	975	31,5	3,230769231	147g0000050.1	975	-18,5	-1,8974358974

148g0000040.1	Unknown protein	965	19	1,968911917	148g0000040.1	965	-50	-5,18134715	148g0000040.1	965	33	3,419689119	148g0000040.1	965	-12,5	-1,295336788
149g0000040.1	Pirin-like protein	908	20,5	2,257709251	149g0000040.1	908	-57	-6,27753304	149g0000040.1	908	22,5	2,477973568	149g0000040.1	908	-21,5	-2,36784141
14g0000110.1	Homogentisate 1,2-dioxygenase	1940	86,5	4,458762887	14g0000110.1	1940	-129,5	-6,675257732	14g0000110.1	1940	27	1,391752577	14g0000110.1	1940	-22	-1,134020619
14g0000210.1	Isoprenyl transferase 2	315	16,5	5,238095238	14g0000210.1	315	-28	-8,888888889	14g0000210.1	315	6	1,904761905	14g0000210.1	315	-6	-1,904761905
14g0000230.1	Acetylxylan esterase	1252	34	2,715654952	14g0000230.1	1252	-65,5	-5,231629393	14g0000230.1	1252	41	3,274760383	14g0000230.1	1252	-31,5	-2,515974441
150g0000040.1	Vanillin dehydrogenase	1472	42,5	2,887228261	150g0000040.1	1472	-132,5	-9,001358696	150g0000040.1	1472	17,5	1,188858696	150g0000040.1	1472	-35	-2,377717391
150g0000060.1	Kelch domain-containing protein 3	2949	53,5	1,814174296	150g0000060.1	2949	-170,5	-5,781620888	150g0000060.1	2949	56,5	1,915903696	150g0000060.1	2949	-46	-1,559850797
151g0000070.1	Pectate lyase H	1356	20,5	1,51179941	151g0000070.1	1356	-64	-4,719764012	151g0000070.1	1356	26	1,91740413	151g0000070.1	1356	-19	-1,401179941
151g0000120.1	Lysosomal Pro-X carboxypeptidase	2164	55,5	2,564695009	151g0000120.1	2164	-141,5	-6,538817006	151g0000120.1	2164	50,5	2,333641405	151g0000120.1	2164	-54	-2,495378928
152g0000110.1	Aprataxin-like protein	878	78,5	8,940774488	152g0000110.1	878	-62,5	-7,118451025	152g0000110.1	878	12	1,366742597	152g0000110.1	878	-11,5	-1,309794989
153g0000090.1	Cutinase	1045	14,5	1,387559809	153g0000090.1	1045	-70	-6,698564593	153g0000090.1	1045	29	2,775119617	153g0000090.1	1045	-26,5	-2,535885168
154g0000100.1	RNA pyrophosphohydrolase	612	61	9,967320261	154g0000100.1	612	-26	-4,248366013	154g0000100.1	612	32	5,22875817	154g0000100.1	612	-8	-1,307189543
155g0000100.1	Subtilisin	2250	31,5	1,4	155g0000100.1	2250	-237,5	-10,55555556	155g0000100.1	2250	49,5	2,2	155g0000100.1	2250	-81	-3,6
157g0000050.1	Coiled-coil domain-containing protein 154	2253	80	3,550821127	157g0000050.1	2253	-138	-6,125166445	157g0000050.1	2253	48	2,130492676	157g0000050.1	2253	-27,5	-1,220594763
157g0000190.1	Nucleolar protein 6	3465	57,5	1,65945166	157g0000190.1	3465	-189,5	-5,468975469	157g0000190.1	3465	73	2,106782107	157g0000190.1	3465	-68	-1,962481963
158g0000050.1	UDP-glucose 4-epimerase	1122	11,5	1,024955437	158g0000050.1	1122	-57,5	-5,124777184	158g0000050.1	1122	13	1,158645276	158g0000050.1	1122	-19,5	-1,737967914
159g0000060.1	Unknown protein	1248	14,5	1,161858974	159g0000060.1	1248	-69	-5,528846154	159g0000060.1	1248	16	1,282051282	159g0000060.1	1248	-13,5	-1,081730769
159g0000130.1	Unknown protein	4202	89,5	2,129938125	159g0000130.1	4202	-260,5	-6,199428843	159g0000130.1	4202	101,5	2,415516421	159g0000130.1	4202	-64	-1,523084246
159g0000140.1	Unknown protein	2005	23	1,14713217	159g0000140.1	2005	-139,5	-6,957605985	159g0000140.1	2005	51	2,543640898	159g0000140.1	2005	-77,5	-3,865336658
159g0000160.1	Unknown protein	3318	60	1,808318264	159g0000160.1	3318	-171,5	-5,168776371	159g0000160.1	3318	45,5	1,371308017	159g0000160.1	3318	-35	-1,054852321
15g0000030.1	Altered inheritance of mitochondria protein 4	323	13,5	4,179566564	15g0000030.1	323	-18,5	-5,72755418	15g0000030.1	323	6,5	2,012383901	15g0000030.1	323	-7	-2,167182663
15g0000050.1	Trihydroxynaphthalene reductase	1403	27,5	1,960085531	15g0000050.1	1403	-94	-6,699928724	15g0000050.1	1403	14,5	1,033499644	15g0000050.1	1403	-19	-1,354240912
15g0000190.1	Ferric reductase transmembrane component 1	1937	133	6,866288074	15g0000190.1	1937	-77,5	-4,001032525	15g0000190.1	1937	27,5	1,419721218	15g0000190.1	1937	-23,5	-1,213216314
160g0000050.1	Proline--tRNA ligase	1010	33	3,267326733	160g0000050.1	1010	-82	-8,118811881	160g0000050.1	1010	18,5	1,831683168	160g0000050.1	1010	-16	-1,584158416
161g0000050.1	N-terminal acetyltransferase B complex subunit arm1	2496	55,5	2,223557692	161g0000050.1	2496	-196	-7,852564103	161g0000050.1	2496	41,5	1,662660256	161g0000050.1	2496	-63	-2,524038462
161g0000120.1	Chaperone protein DnaJ	1865	22	1,179624665	161g0000120.1	1865	-87	-4,664879357	161g0000120.1	1865	24	1,286863271	161g0000120.1	1865	-81	-4,343163539
162g0000080.1	Unknown protein	737	17	2,306648575	162g0000080.1	737	-35	-4,748982361	162g0000080.1	737	13	1,763907734	162g0000080.1	737	-29	-3,934871099
162g0000090.1	Unknown protein	1272	15	1,179245283	162g0000090.1	1272	-75,5	-5,935534591	162g0000090.1	1272	15,5	1,218553459	162g0000090.1	1272	-42,5	-3,341194969
162g0000150.1	Unknown protein	1854	32	1,725997843	162g0000150.1	1854	-92,5	-4,989212514	162g0000150.1	1854	65	3,505933118	162g0000150.1	1854	-20,5	-1,105717368
163g0000050.1	Branched-chain-amino-acid aminotransferase	1727	27	1,563404748	163g0000050.1	1727	-113,5	-6,57209033	163g0000050.1	1727	31	1,795020266	163g0000050.1	1727	-36	-2,084539664
164g0000040.1	Histone acetyltransferase mst2	3499	61,5	1,757645041	164g0000040.1	3499	-179,5	-5,130037154	164g0000040.1	3499	84,5	2,414975707	164g0000040.1	3499	-60	-1,71477565
164g0000050.1	Sulfate adenylyltransferase subunit 2	979	33	3,370786517	164g0000050.1	979	-66	-6,741573034	164g0000050.1	979	20	2,042900919	164g0000050.1	979	-49,5	-5,056179775
164g0000180.1	FRAS1-related extracellular matrix protein 1	1300	18	1,384615385	164g0000180.1	1300	-105,5	-8,115384615	164g0000180.1	1300	33	2,538461539	164g0000180.1	1300	-33	-2,538461539
166g0000060.1	Unknown protein	721	14,5	2,0110957	166g0000060.1	721	-30	-4,160887656	166g0000060.1	721	27,5	3,814147018	166g0000060.1	721	-13	-1,803051318
168g0000150.1	Unknown protein	931	39,5	4,242749732	168g0000150.1	931	-34,5	-3,705692803	168g0000150.1	931	12,5	1,34264232	168g0000150.1	931	-20,5	-2,201933405
168g0000170.1	Unknown protein	2088	70	3,352490422	168g0000170.1	2088	-93,5	-4,477969349	168g0000170.1	2088	38	1,819923372	168g0000170.1	2088	-26	-1,245210728
168g0000190.1	Unknown protein	5906	73	1,236031155	168g0000190.1	5906	-396	-6,705045716	168g0000190.1	5906	99	1,676261429	168g0000190.1	5906	-168	-2,844564849
169g0000050.1	Eukaryotic translation initiation factor 3 subunit C	536	5,5	1,026119403	169g0000050.1	536	-19,5	-3,638059702	169g0000050.1	536	18,5	3,451492537	169g0000050.1	536	-11,5	-2,145522388
169g0000130.1	50S ribosomal protein L23	1992	26,5	1,330321285	169g0000130.1	1992	-142	-7,128514056	169g0000130.1	1992	33	1,656626506	169g0000130.1	1992	-65	-3,263052209
16g0000020.1	Haloacid dehalogenase-like hydrolase domain-containing 5	1103	20,5	1,858567543	16g0000020.1	1103	-85	-7,706255666	16g0000020.1	1103	18	1,631912965	16g0000020.1	1103	-59,5	-5,394378967
16g0000100.1	Short chain dehydrogenase atnD	1221	29	2,375102375	16g0000100.1	1221	-44	-3,603603604	16g0000100.1	1221	24,5	2,006552007	16g0000100.1	1221	-28	-2,293202293
16g0000180.1	ATP-dependent DNA helicase DDM1	2792	72	2,578796562	16g0000180.1	2792	-61	-2,184813754	16g0000180.1	2792	70	2,507163324	16g0000180.1	2792	-63,5	-2,274355301
170g0000070.1	Chitin deacetylase ARB_04768	1595	73,5	4,60815047	170g0000070.1	1595	-107	-6,70846395	170g0000070.1	1595	20	1,253918495	170g0000070.1	1595	-35	-2,194357367
171g0000040.1	Unknown protein	1565	107	6,837060703	171g0000040.1	1565	-154	-9,840255591	171g0000040.1	1565	31,5	2,012779553	171g0000040.1	1565	-56,5	-3,610223642
171g0000120.1	Unknown protein	950	61	6,421052632	171g0000120.1	950	-76,5	-8,052631579	171g0000120.1	950	16	1,684210526	171g0000120.1	950	-20	-2,105263158
172g0000080.1	Protein unc-45 homolog B	2188	73	3,336380256	172g0000080.1	2188	-132,5	-6,055758684	172g0000080.1	2188	27	1,234003656	172g0000080.1	2188	-57	-2,60511883
172g0000120.1	GDP-Man:Man(3)GlcNAc(2)-PP-Dol alpha-1,2-mannosyltransferase	3348	34	1,015531661	172g0000120.1	3348	-247	-7,377538829	172g0000120.1	3348	63,5	1,896654719	172g0000120.1	3348	-71,5	-2,135603345
173g0000050.1	Unknown protein	731	11,5	1,573187415	173g0000050.1	731	-36	-4,924760602	173g0000050.1	731	18,5	2,530779754	173g0000050.1	731	-20,5	-2,804377565
173g0000120.1	Unknown protein	393	9	2,290076336	173g0000120.1	393	-34	-8,651399491	173g0000120.1	393	8	2,03562341	173g0000120.1	393	-5,5	-1,399491094
174g0000020.1	Unknown protein	320	14	4,375	174g0000020.1	320	-3,5	-1,09375	174g0000020.1	320	7,5	2,34375	174g0000020.1	320	-8,5	-2,65625
174g0000040.1	Unknown protein	846	25,5	3,014184397	174g0000040.1	846	-52	-6,146572104	174g0000040.1	846	9	1,063829787	174g0000040.1	846	-26	-3,073286052
175g0000070.1	Oxygen-dependent choline dehydrogenase	1672	100	5,980861244	175g0000070.1	1672	-108	-6,459330144	175g0000070.1	1672	52	3,110047847	175g0000070.1	1672	-35	-2,093301435
177g0000090.1	WD repeat-containing protein JIP5	709	7,5	1,057827927	177g0000090.1	709	-30	-4,231311707	177g0000090.1	709	20,5	2,891396333	177g0000090.1	709	-11	-1,551480959
178g0000010.1	Unknown protein	2333	43,5	1,864552079	178g0000010.1	2333	-153,5	-6,579511359	178g0000010.1	2333	43	1,843120446	178g0000010.1	2333	-81,5	-3,493356194
179g0000040.1	Histidinol-phosphate aminotransferase	1184	40,5	3,420608108	179g0000040.1	1184	-71	-5,996621622	179g0000040.1	1184	14,5	1,224662162	179g0000040.1	1184	-18,5	-1,5625
17g0000040.1	Proline dehydrogenase 1, mitochondrial	1832	18,5	1,009825328	17g0000040.1	1832	-91,5	-4,994541485	17g0000040.1	1832	48,5	2,647379913	17g0000040.1	1832	-46	-2,510917031
17g0000140.1	Vacuolar protein-sorting protein BRO1	960	26,5	2,760416667	17g0000140.1	960	-73	-7,604166667	17g0000140.1	960	30	3,125	17g0000140.1	960	-20,5	-2,135416667
1809g0000010.1	DNA-directed RNA polymerase subunit K	747	21	2,81124498	1809g0000010.1	747	-38,5	-5,15394913	1809g0000010.1	747	10,5	1,40562249	1809g0000010.1	747	-21,5	-2,878179384
180g0000060.1	Urease accessory protein UreE	762	31	4,06824147	180g0000060.1	762	-23	-3,018372703	180g0000060.1	762	22	2,887139108	180g0000060.1	762	-15	-1,968503937
181g0000070.1	50S ribosomal protein L1	779	20,5	2,631578947	181g0000070.1	779	-33,5	-4,300385109	181g0000070.1	779	48,5	6,22593068	181g0000070.1	779	-26	-3,337612324
181g0000130.1	Peptide-N(4)-(N-acetyl-beta-glucosaminyl)asparagine amidase	230	94,5	41,08695652	181g0000130.1	230	-12	-5,217391304	181g0000130.1	230	2,5	1,086956522	181g0000130.1	230	-24	-10,43478261
182g0000030.1	GPI mannosyltransferase 2	335	16	4,776119403	182g0000030.1	335	-37	-11,04477612	182g0000030.1	335	10	2,985074627	182g0000030.1	335	-7,5	-2,23880597

182g0000050.1	Chitin synthase 1	2238	52,5	2,345844504	182g0000050.1	2238	-137	-6,121537087	182g0000050.1	2238	80	3,574620197	182g0000050.1	2238	-31	-1,385165326
183g0000120.1	Unknown protein	208	50,5	24,27884615	183g0000120.1	208	-14	-6,730769231	183g0000120.1	208	9,5	4,567307692	183g0000120.1	208	-13,5	-6,490384615
184g0000040.1	Unknown protein	2390	30,5	1,276150628	184g0000040.1	2390	-133,5	-5,585774059	184g0000040.1	2390	71,5	2,991631799	184g0000040.1	2390	-40	-1,673640167
184g0000130.1	Unknown protein	839	53	6,3170441	184g0000130.1	839	-13	-1,549463647	184g0000130.1	839	29,5	3,516090584	184g0000130.1	839	-18,5	-2,20500596
184g0000180.1	Unknown protein	329	92	27,96352584	184g0000180.1	329	-27,5	-8,358662614	184g0000180.1	329	4	1,215805471	184g0000180.1	329	-20,5	-6,23100304
184g0000230.1	Unknown protein	1412	42	2,974504249	184g0000230.1	1412	-108,5	-7,684135977	184g0000230.1	1412	19,5	1,38101983	184g0000230.1	1412	-18	-1,274787535
185g0000040.1	Unknown protein	3071	104,5	3,402800391	185g0000040.1	3071	-180,5	-5,877564311	185g0000040.1	3071	31,5	1,02572452	185g0000040.1	3071	-69	-2,246825138
186g0000050.1	tRNA uridine 5-carboxymethylaminomethyl modification enzyme MnmG	708	94	13,27683616	186g0000050.1	708	-34,5	-4,872881356	186g0000050.1	708	18,5	2,61299435	186g0000050.1	708	-24	-3,389830509
186g0000070.1	FAD-dependent monooxygenase OpS4	1405	41	2,918149466	186g0000070.1	1405	-76	-5,409252669	186g0000070.1	1405	16	1,138790036	186g0000070.1	1405	-35	-2,491103203
186g0000080.1	Imidazole glycerol phosphate synthase subunit HisF	1272	55,5	4,363207547	186g0000080.1	1272	-112,5	-8,844339623	186g0000080.1	1272	20	1,572327044	186g0000080.1	1272	-34	-2,672955975
187g0000090.1	UDP-N-acetylmuramoylalanine--D-glutamate ligase	867	52	5,997693195	187g0000090.1	867	-21	-2,422145329	187g0000090.1	867	11	1,268742791	187g0000090.1	867	-29	-3,344867359
188g0000010.1	Unknown protein	4187	57	1,36135658	188g0000010.1	4187	-294	-7,021733938	188g0000010.1	4187	98	2,34057798	188g0000010.1	4187	-109,5	-2,61523764
188g0000070.1	Unknown protein	432	33,5	7,75462963	188g0000070.1	432	-10	-2,314814815	188g0000070.1	432	27,5	6,365740741	188g0000070.1	432	-6	-1,388888889
188g0000130.1	Unknown protein	1989	44,5	2,237305179	188g0000130.1	1989	-95	-4,776269482	188g0000130.1	1989	51	2,564102564	188g0000130.1	1989	-22,5	-1,13122172
188g0000160.1	Unknown protein	3202	50	1,561524048	188g0000160.1	3202	-135	-4,216114928	188g0000160.1	3202	42	1,3116802	188g0000160.1	3202	-124,5	-3,888194878
189g0000010.1	Unknown protein	1426	43,5	3,050490884	189g0000010.1	1426	-78	-5,469845722	189g0000010.1	1426	28	1,963534362	189g0000010.1	1426	-49	-3,436185133
18g0000130.1	Brefeldin A-inhibited guanine nucleotide-exchange protein 2	2527	122	4,827859122	18g0000130.1	2527	-144,5	-5,718242976	18g0000130.1	2527	45,5	1,800554017	18g0000130.1	2527	-58,5	-2,314998021
18g0000190.1	RNA polymerase sigma-54 factor	603	15	2,487562189	18g0000190.1	603	-30,5	-5,058043118	18g0000190.1	603	11	1,824212272	18g0000190.1	603	-15	-2,487562189
191g0000040.1	HMG box-containing protein C19G7.04	803	33	4,109589041	191g0000040.1	803	-59,5	-7,409713574	191g0000040.1	803	18	2,241594022	191g0000040.1	803	-21,5	-2,677459527
191g0000070.1	Valine--tRNA ligase	4058	84,5	2,082306555	191g0000070.1	4058	-206,5	-5,088713652	191g0000070.1	4058	87	2,143913258	191g0000070.1	4058	-55,5	-1,367668802
191g0000130.1	EEF1A lysine methyltransferase 1	612	36	5,882352941	191g0000130.1	612	-31	-5,065359477	191g0000130.1	612	15	2,450980392	191g0000130.1	612	-27	-4,411764706
192g0000040.1	Unknown protein	2459	26,5	1,077673851	192g0000040.1	2459	-142	-5,774705165	192g0000040.1	2459	27,5	1,118340789	192g0000040.1	2459	-102,5	-4,168361122
192g0000130.1	Unknown protein	650	23,5	3,615384615	192g0000130.1	650	-25,5	-3,923076923	192g0000130.1	650	9	1,384615385	192g0000130.1	650	-20,5	-3,153846154
193g0000060.1	3-ketodihydrosphingosine reductase gsl-3	1406	42	2,987197724	193g0000060.1	1406	-153,5	-10,91749644	193g0000060.1	1406	30,5	2,169274538	193g0000060.1	1406	-17,5	-1,244665718
194g0000080.1	Unknown protein	1593	82	5,147520402	194g0000080.1	1593	-56	-3,515379787	194g0000080.1	1593	44,5	2,793471438	194g0000080.1	1593	-18	-1,129943503
195g0000020.1	Unknown protein	1316	32,5	2,469604863	195g0000020.1	1316	-73,5	-5,585106383	195g0000020.1	1316	19	1,443768997	195g0000020.1	1316	-17	-1,291793313
195g0000090.1	Unknown protein	1244	33	2,652733119	195g0000090.1	1244	-66	-5,305466238	195g0000090.1	1244	37	2,974276527	195g0000090.1	1244	-17,5	-1,406752412
1964g0000010.1	Protein LIGHT-DEPENDENT SHORT HYPOCOTYLS 5	215	32	14,88372093	1964g0000010.1	215	-8,5	-3,953488372	1964g0000010.1	215	12	5,581395349	1964g0000010.1	215	-3	-1,395348837
196g0000080.1	Chromodomain Y-like protein 2	4091	43	1,051087754	196g0000080.1	4091	-172,5	-4,216572965	196g0000080.1	4091	118	2,884380347	196g0000080.1	4091	-49	-1,197751161
196g0000090.1	Forkhead-associated domain-containing protein 1	1473	84	5,702647658	196g0000090.1	1473	-65	-4,412763069	196g0000090.1	1473	17,5	1,188051595	196g0000090.1	1473	-37,5	-2,545824847
199g0000050.1	Glucosamine-6-phosphate deaminase	1499	33,5	2,234823216	199g0000050.1	1499	-81	-5,403602402	199g0000050.1	1499	19	1,267511674	199g0000050.1	1499	-40	-2,66844563
19g0000020.1	Unknown protein	873	57,5	6,586483391	19g0000020.1	873	-71	-8,132875143	19g0000020.1	873	15,5	1,775486827	19g0000020.1	873	-24	-2,749140894
19g0000140.1	Unknown protein	1172	28	2,389078498	19g0000140.1	1172	-93,5	-7,9778157	19g0000140.1	1172	13	1,109215017	19g0000140.1	1172	-15	-1,279863481
1g0000340.1	Unknown protein	415	75,5	18,19277108	1g0000340.1	415	-27	-6,506024096	1g0000340.1	415	12,5	3,012048193	1g0000340.1	415	-14,5	-3,493975904
1g0000440.1	Unknown protein	2535	85,5	3,372781065	1g0000440.1	2535	-61,5	-2,426035503	1g0000440.1	2535	72	2,840236686	1g0000440.1	2535	-55	-2,169625247
200g0000050.1	L-lactate dehydrogenase	456	80,5	17,65350877	200g0000050.1	456	-57,5	-12,60964912	200g0000050.1	456	8	1,754385965	200g0000050.1	456	-8,5	-1,864035088
201g0000060.1	Unknown protein	328	11,5	3,506097561	201g0000060.1	328	-24	-7,317073171	201g0000060.1	328	11,5	3,506097561	201g0000060.1	328	-15,5	-4,725609756
201g0000140.1	Unknown protein	2874	90	3,131524008	201g0000140.1	2874	-158	-5,49756437	201g0000140.1	2874	58,5	2,035490605	201g0000140.1	2874	-75,5	-2,627000696
202g0000060.1	Unknown protein	920	76	8,260869565	202g0000060.1	920	-54,5	-5,923913044	202g0000060.1	920	23	2,5	202g0000060.1	920	-25	-2,717391304
202g0000150.1	Unknown protein	1613	67,5	4,184748915	202g0000150.1	1613	-64	-3,967761934	202g0000150.1	1613	24	1,487910725	202g0000150.1	1613	-21,5	-1,332920025
203g0000080.1	Unknown protein	1555	39	2,508038585	203g0000080.1	1555	-102	-6,559485531	203g0000080.1	1555	23	1,479099679	203g0000080.1	1555	-61,5	-3,954983923
203g0000090.1	Unknown protein	2284	30	1,313485114	203g0000090.1	2284	-113,5	-4,969352014	203g0000090.1	2284	59	2,583187391	203g0000090.1	2284	-52	-2,276707531
203g0000160.1	Unknown protein	717	28,5	3,974895398	203g0000160.1	717	-40	-5,578800558	203g0000160.1	717	30,5	4,253835425	203g0000160.1	717	-10,5	-1,464435146
204g0000030.1	Unknown protein	1063	27,5	2,587017874	204g0000030.1	1063	-25,5	-2,39887112	204g0000030.1	1063	37	3,480714958	204g0000030.1	1063	-42	-3,951081844
205g0000090.1	S-phase delaying protein 2	740	32	4,324324324	205g0000090.1	740	-41,5	-5,608108108	205g0000090.1	740	28,5	3,851351351	205g0000090.1	740	-8	-1,081081081
205g0000140.1	Unknown protein	1672	26	1,555023923	205g0000140.1	1672	-84	-5,023923445	205g0000140.1	1672	26,5	1,58492823	205g0000140.1	1672	-56,5	-3,379186603
206g0000050.1	N-glycosylase/DNA lyase	1375	21,5	1,563636364	206g0000050.1	1375	-89	-6,472727273	206g0000050.1	1375	35,5	2,581818182	206g0000050.1	1375	-27,5	-2
206g0000070.1	U6 snRNA-associated Sm-like protein LSm2	1781	121,5	6,822010107	206g0000070.1	1781	-111	-6,232453678	206g0000070.1	1781	33,5	1,88096575	206g0000070.1	1781	-45	-2,52667041
206g0000080.1	WD repeat-containing protein 3	2939	76,5	2,602926165	206g0000080.1	2939	-220,5	-7,502551888	206g0000080.1	2939	40,5	1,378019735	206g0000080.1	2939	-110,5	-3,759782239
20g0000110.1	Indole-3-glycerol phosphate synthase	2321	88,5	3,813011633	20g0000110.1	2321	-135	-5,816458423	20g0000110.1	2321	40	1,723395088	20g0000110.1	2321	-43,5	-1,874192159
20g0000180.1	Meiosis-specific serine/threonine-protein kinase mek1	861	35,5	4,12311266	20g0000180.1	861	-24,5	-2,845528455	20g0000180.1	861	13,5	1,567944251	20g0000180.1	861	-25,5	-2,961672474
20g0000270.1	Ribosome maturation protein SBDS	947	68,5	7,233368532	20g0000270.1	947	-51,5	-5,438225977	20g0000270.1	947	18	1,900739176	20g0000270.1	947	-10,5	-1,10876452
210g0000100.1	Unknown protein	2687	29	1,079270562	210g0000100.1	2687	-109	-4,056568664	210g0000100.1	2687	30,5	1,135094901	210g0000100.1	2687	-29	-1,079270562
210g0000180.1	Unknown protein	2464	118,5	4,809253247	210g0000180.1	2464	-108	-4,383116883	210g0000180.1	2464	66,5	2,698863636	210g0000180.1	2464	-36	-1,461038961
211g0000050.1	Satratoxin biosynthesis SC1 cluster protein 4	1731	38,5	2,224147891	211g0000050.1	1731	-69,5	-4,01502022	211g0000050.1	1731	30	1,733102253	211g0000050.1	1731	-37,5	-2,166377816
211g0000070.1	Equisetin cluster transcription factor eqxF	2025	22	1,086419753	211g0000070.1	2025	-97,5	-4,814814815	211g0000070.1	2025	35	1,728395062	211g0000070.1	2025	-22,5	-1,111111111
212g0000040.1	Exopolygalacturonase	2060	44	2,13592233	212g0000040.1	2060	-80	-3,883495146	212g0000040.1	2060	39,5	1,917475728	212g0000040.1	2060	-48	-2,330097087
212g0000060.1	Pyruvate dehydrogenase E1 component subunit alpha-1, mitochondria	369	11,5	3,116531165	212g0000060.1	369	-9,5	-2,574525745	212g0000060.1	369	13	3,52303523	212g0000060.1	369	-5	-1,35501355
213g0000040.1	Malate synthase, glyoxysomal	1682	49	2,913198573	213g0000040.1	1682	-130,5	-7,75862069	213g0000040.1	1682	42	2,497027348	213g0000040.1	1682	-19,5	-1,159334126
214g0000130.1	Unknown protein	637	31	4,866562009	214g0000130.1	637	-37	-5,808477237	214g0000130.1	637	28	4,395604396	214g0000130.1	637	-18,5	-2,904238619
215g0000040.1	Unknown protein	1506	21,5	1,427622842	215g0000040.1	1506	-90,5	-6,009296149	215g0000040.1	1506	33,5	2,224435591	215g0000040.1	1506	-34,5	-2,290836653
216g0000050.1	Vacuolar protein sorting/targeting protein 10	1390	27	1,942446043	216g0000050.1	1390	-30	-2,158273381	216g0000050.1	1390	36,5	2,625899281	216g0000050.1	1390	-41,5	-2,985611511

217g0000110.1	Arginine biosynthesis bifunctional protein ArgJ, mitochondria	1394	22	1,578192253	217g0000110.1	1394	-121,5	-8,715925395	217g0000110.1	1394	22	1,578192253	217g0000110.1	1394	-17,5	-1,255380201
217g0000160.1	Pre-mRNA-splicing factor CWC2	1879	75	3,991484832	217g0000160.1	1879	-99	-5,268759979	217g0000160.1	1879	42	2,235231506	217g0000160.1	1879	-48,5	-2,581160192
217g0000180.1	Cytochrome c oxidase subunit 3	398	8	2,010050251	217g0000180.1	398	-21,5	-5,40201005	217g0000180.1	398	11,5	2,889447236	217g0000180.1	398	-15	-3,768844221
218g0000050.1	High osmolarity signaling protein SHO1	3437	43,5	1,265638638	218g0000050.1	3437	-225	-6,54640675	218g0000050.1	3437	43,5	1,265638638	218g0000050.1	3437	-53,5	-1,55659005
21g0000060.1	Unknown protein	1174	35,5	3,023850085	21g0000060.1	1174	-63	-5,366269165	21g0000060.1	1174	41,5	3,534923339	21g0000060.1	1174	-17,5	-1,490630324
21g0000170.1	Unknown protein	441	29,5	6,689342404	21g0000170.1	441	-36,5	-8,276643991	21g0000170.1	441	20	4,535147392	21g0000170.1	441	-9,5	-2,154195011
21g0000240.1	Unknown protein	2042	26	1,273261508	21g0000240.1	2042	-113	-5,533790402	21g0000240.1	2042	63,5	3,109696376	21g0000240.1	2042	-29,5	-1,444662096
220g0000070.1	Homeobox protein 4	2794	29	1,03793844	220g0000070.1	2794	-184	-6,585540444	220g0000070.1	2794	79,5	2,845382964	220g0000070.1	2794	-51	-1,825340014
220g0000090.1	Penaeidin-3g	939	47	5,005324814	220g0000090.1	939	-116,5	-12,40681576	220g0000090.1	939	28,5	3,03514377	220g0000090.1	939	-11,5	-1,224707135
221g0000010.1	Acetyl-coenzyme A synthetase	1933	20	1,034661149	221g0000010.1	1933	-279	-14,43352302	221g0000010.1	1933	20,5	1,060527677	221g0000010.1	1933	-188,5	-9,751681324
221g0000040.1	Peroxisomal hydratase-dehydrogenase-epimerase	3124	50	1,600512164	221g0000040.1	3124	-222,5	-7,122279129	221g0000040.1	3124	54	1,728553137	221g0000040.1	3124	-74	-2,368758003
221g0000080.1	Heterokaryon incompatibility protein 6, OR allele	1272	31,5	2,476415094	221g0000080.1	1272	-105	-8,254716981	221g0000080.1	1272	19,5	1,533018868	221g0000080.1	1272	-37,5	-2,948113208
221g0000090.1	ATP synthase subunit delta	684	81,5	11,91520468	221g0000090.1	684	-45,5	-6,652046784	221g0000090.1	684	12	1,754385965	221g0000090.1	684	-14	-2,046783626
221g0000110.1	Pheromone-regulated membrane protein 10	2789	67,5	2,420222302	221g0000110.1	2789	-199,5	-7,15310147	221g0000110.1	2789	70,5	2,527787738	221g0000110.1	2789	-42,5	-1,523843672
222g0000060.1	Meiotically up-regulated gene 190 protein	3976	63,5	1,597082495	222g0000060.1	3976	-310,5	-7,809356137	222g0000060.1	3976	43	1,081488934	222g0000060.1	3976	-96,5	-2,427062374
223g0000120.1	Mitochondrial distribution and morphology protein 12	1754	103	5,872291904	223g0000120.1	1754	-88,5	-5,045610034	223g0000120.1	1754	24,5	1,396807298	223g0000120.1	1754	-35	-1,995438997
224g0000090.1	Unknown protein	960	53	5,520833333	224g0000090.1	960	-24,5	-2,552083333	224g0000090.1	960	19	1,979166667	224g0000090.1	960	-10	-1,041666667
225g0000060.1	Glutathione-dependent formaldehyde-activating enzyme	565	12	2,123893805	225g0000060.1	565	-59,5	-10,53097345	225g0000060.1	565	17,5	3,097345133	225g0000060.1	565	-18	-3,185840708
226g0000070.1	Unknown protein	3149	33,5	1,063829787	226g0000070.1	3149	-192,5	-6,113051763	226g0000070.1	3149	55	1,746586218	226g0000070.1	3149	-48,5	-1,540171483
227g0000050.1	30S ribosomal protein S6	663	9	1,357466063	227g0000050.1	663	-34	-5,128205128	227g0000050.1	663	31	4,67571644	227g0000050.1	663	-11	-1,659125189
227g0000070.1	DNA repair protein RAD5	3795	53	1,39657444	227g0000070.1	3795	-244,5	-6,442687747	227g0000070.1	3795	77	2,028985507	227g0000070.1	3795	-85,5	-2,252964427
227g0000080.1	Gonadotropin-releasing hormone II receptor	1081	42,5	3,931544866	227g0000080.1	1081	-89,5	-8,279370953	227g0000080.1	1081	20,5	1,896392229	227g0000080.1	1081	-34,5	-3,191489362
227g0000150.1	Phosphorus acquisition-controlling protein	2823	88	3,117251151	227g0000150.1	2823	-110	-3,896563939	227g0000150.1	2823	34	1,20439249	227g0000150.1	2823	-70,5	-2,497343252
229g0000110.1	Calmodulin	680	94	13,82352941	229g0000110.1	680	-30,5	-4,485294118	229g0000110.1	680	21	3,088235294	229g0000110.1	680	-23	-3,382352941
22g0000010.1	Cytochrome c oxidase subunit 5A, mitochondrial	1270	36	2,834645669	22g0000010.1	1270	-31	-2,440944882	22g0000010.1	1270	39,5	3,110236221	22g0000010.1	1270	-45,5	-3,582677165
230g0000030.1	Elongation factor 1-alpha	1301	56	4,304381245	230g0000030.1	1301	-83,5	-6,418139892	230g0000030.1	1301	15	1,152959262	230g0000030.1	1301	-17,5	-1,345119139
230g0000070.1	1-deoxy-D-xylulose 5-phosphate reductoisomerase	545	50	9,174311927	230g0000070.1	545	-32,5	-5,963302752	230g0000070.1	545	21,5	3,944954128	230g0000070.1	545	-15	-2,752293578
231g0000030.1	Phosphoenolpyruvate/phosphate translocator 1, chloroplastic	2465	33	1,338742394	231g0000030.1	2465	-203	-8,235294118	231g0000030.1	2465	30,5	1,237322515	231g0000030.1	2465	-51,5	-2,089249493
233g0000010.1	Maintenance of mitochondrial morphology protein 1	3599	45	1,250347319	233g0000010.1	3599	-278	-7,72436788	233g0000010.1	3599	54	1,500416782	233g0000010.1	3599	-44,5	-1,236454571
233g0000120.1	Polyadenylate-binding protein 3	2083	21	1,008161306	233g0000120.1	2083	-210,5	-10,1056169	233g0000120.1	2083	41,5	1,992318771	233g0000120.1	2083	-90,5	-4,344695151
235g0000100.1	Unknown protein	758	33	4,353562005	235g0000100.1	758	-55	-7,255936676	235g0000100.1	758	15	1,978891821	235g0000100.1	758	-31	-4,089709763
236g0000030.1	Unknown protein	1323	38,5	2,91005291	236g0000030.1	1323	-74	-5,593348451	236g0000030.1	1323	23,5	1,776266062	236g0000030.1	1323	-13,5	-1,020408163
238g0000030.1	Cleavage and polyadenylation specificity factor subunit 2	3335	61,5	1,844077961	238g0000030.1	3335	-237	-7,106446777	238g0000030.1	3335	77,5	2,323838081	238g0000030.1	3335	-50,5	-1,514242879
238g0000070.1	Ubiquitin-conjugating enzyme E2 E2	883	29	3,284258211	238g0000070.1	883	-50,5	-5,719139298	238g0000070.1	883	22	2,491506229	238g0000070.1	883	-25	-2,831257078
239g0000020.1	Trafficking protein particle complex II-specific subunit 120 homolog	1658	25,5	1,537997588	239g0000020.1	1658	-79	-4,76477684	239g0000020.1	1658	33,5	2,020506635	239g0000020.1	1658	-77	-4,644149578
239g0000070.1	Cutinase	945	27,5	2,91005291	239g0000070.1	945	-33,5	-3,544973545	239g0000070.1	945	27,5	2,91005291	239g0000070.1	945	-14,5	-1,534391534
239g0000080.1	ABC transporter G family member 11	5811	174,5	3,002925486	239g0000080.1	5811	-448	-7,709516434	239g0000080.1	5811	69,5	1,196007572	239g0000080.1	5811	-110	-1,892961625
23g0000260.1	Ubiquitin thioesterase OTU1	1393	56	4,020100503	23g0000260.1	1393	-108	-7,753050969	23g0000260.1	1393	14	1,005025126	23g0000260.1	1393	-45,5	-3,266331658
23g0000270.1	Chromatin structure-remodeling complex subunit RSC1	3872	93,5	2,414772727	23g0000270.1	3872	-239	-6,172520661	23g0000270.1	3872	117,5	3,034607438	23g0000270.1	3872	-45,5	-1,175103306
240g0000030.1	Unknown protein	932	64,5	6,920600858	240g0000030.1	932	-91,5	-9,817596567	240g0000030.1	932	21	2,253218884	240g0000030.1	932	-9,5	-1,019313305
240g0000040.1	Unknown protein	1914	58	3,03030303	240g0000040.1	1914	-152,5	-7,967607106	240g0000040.1	1914	48,5	2,533960293	240g0000040.1	1914	-43	-2,246603971
241g0000150.1	ATP synthase subunit a 2	2107	24	1,139060275	241g0000150.1	2107	-193	-9,159943047	241g0000150.1	2107	27,5	1,305173232	241g0000150.1	2107	-50,5	-2,396772663
241g0000160.1	Mitochondrial import receptor subunit tom20	636	22,5	3,537735849	241g0000160.1	636	-40,5	-6,367924528	241g0000160.1	636	12	1,886792453	241g0000160.1	636	-9	-1,41509434
243g0000070.1	Nuclear pore complex protein Nup214	942	23	2,441613588	243g0000070.1	942	-81,5	-8,651804671	243g0000070.1	942	9,5	1,008492569	243g0000070.1	942	-49	-5,201698514
245g0000050.1	Prolyl 4-hydroxylase subunit alpha-2	886	49,5	5,586907449	245g0000050.1	886	-46	-5,191873589	245g0000050.1	886	30,5	3,442437923	245g0000050.1	886	-31	-3,498871332
245g0000070.1	Transcriptional regulatory protein moc3	2195	50	2,277904328	245g0000070.1	2195	-113	-5,148063781	245g0000070.1	2195	51	2,323462415	245g0000070.1	2195	-44	-2,004555809
246g0000050.1	Protein MCM10 homolog	2327	28	1,203266008	246g0000050.1	2327	-126	-5,414697035	246g0000050.1	2327	25,5	1,095831543	246g0000050.1	2327	-49	-2,105715514
248g0000080.1	Phosphopentomutase	1171	46	3,928266439	248g0000080.1	1171	-130	-11,10162254	248g0000080.1	1171	16,5	1,409052092	248g0000080.1	1171	-25	-2,134927413
248g0000150.1	Potassium-transporting ATPase ATP-binding subunit	720	69	9,583333333	248g0000150.1	720	-53	-7,361111111	248g0000150.1	720	22,5	3,125	248g0000150.1	720	-13,5	-1,875
249g0000110.1	Kelch repeat-containing protein ARB_01230	2003	69,5	3,469795307	249g0000110.1	2003	-158,5	-7,913130305	249g0000110.1	2003	52	2,596105841	249g0000110.1	2003	-35	-1,747378932
24g0000050.1	Unknown protein	1636	86,5	5,287286064	24g0000050.1	1636	-95	-5,806845966	24g0000050.1	1636	34	2,078239609	24g0000050.1	1636	-22,5	-1,375305624
24g0000140.1	Unknown protein	2142	55,5	2,591036415	24g0000140.1	2142	-75	-3,50140056	24g0000140.1	2142	50,5	2,357609711	24g0000140.1	2142	-101	-4,715219421
24g0000190.1	Unknown protein	905	19,5	2,154696133	24g0000190.1	905	-48,5	-5,359116022	24g0000190.1	905	24	2,651933702	24g0000190.1	905	-45,5	-5,027624309
24g0000200.1	Unknown protein	928	31	3,340517241	24g0000200.1	928	-62,5	-6,734913793	24g0000200.1	928	23	2,478448276	24g0000200.1	928	-43,5	-4,6875
24g0000260.1	Unknown protein	1049	78,5	7,483317445	24g0000260.1	1049	-37	-3,527168732	24g0000260.1	1049	17,5	1,668255481	24g0000260.1	1049	-20	-1,906577693
250g0000050.1	Unknown protein	589	72	12,22410866	250g0000050.1	589	-42	-7,130730051	250g0000050.1	589	15	2,546689304	250g0000050.1	589	-19,5	-3,310696095
252g0000030.1	Ubiquitin carboxyl-terminal hydrolase 20	337	98	29,08011869	252g0000030.1	337	-26	-7,715133531	252g0000030.1	337	6	1,78041543	252g0000030.1	337	-3,5	-1,038575668
253g0000100.1	GMP synthase [glutamine-hydrolyzing]	2826	143	5,060155697	253g0000100.1	2826	-139	-4,91861288	253g0000100.1	2826	171,5	6,068648266	253g0000100.1	2826	-39,5	-1,397735315
255g0000040.1	Unknown protein	2236	114,5	5,120751342	255g0000040.1	2236	-143,5	-6,417710197	255g0000040.1	2236	23,5	1,0509839	255g0000040.1	2236	-43	-1,923076923
257g0000010.1	Aldehyde dehydrogenase	1965	37,5	1,908396947	257g0000010.1	1965	-100,5	-5,114503817	257g0000010.1	1965	20,5	1,043256998	257g0000010.1	1965	-37,5	-1,908396947
257g0000050.1	Unknown protein	787	48	6,099110546	257g0000050.1	787	-31,5	-4,002541296	257g0000050.1	787	15	1,905972046	257g0000050.1	787	-9	-1,143583227
258g0000010.1	Carotenoid 9,10(9',10')-cleavage dioxygenase	2303	31	1,346070343	258g0000010.1	2303	-98,5	-4,277029961	258g0000010.1	2303	62	2,692140686	258g0000010.1	2303	-25,5	-1,107251411

258g0000030.1	Potassium channel toxin BmTXK-beta-2	1169	100,5	8,597091531	258g0000030.1	1169	-63,5	-5,431993157	258g0000030.1	1169	21	1,796407186	258g0000030.1	1169	-22	-1,881950385
25g0000250.1	Unknown protein	996	14	1,40562249	25g0000250.1	996	-66,5	-6,676706827	25g0000250.1	996	16,5	1,656626506	25g0000250.1	996	-14,5	-1,455823293
25g0000290.1	Unknown protein	2777	28	1,008282319	25g0000290.1	2777	-225,5	-8,120273677	25g0000290.1	2777	53	1,90853439	25g0000290.1	2777	-35	-1,260352899
25g0000300.1	Unknown protein	1760	64	3,636363636	25g0000300.1	1760	-95,5	-5,426136364	25g0000300.1	1760	72,5	4,119318182	25g0000300.1	1760	-24,5	-1,392045455
260g0000060.1	Solute carrier family 41 member 1	182	8	4,395604396	260g0000060.1	182	-7,5	-4,120879121	260g0000060.1	182	2	1,098901099	260g0000060.1	182	-3,5	-1,923076923
260g0000110.1	Serine/threonine-protein kinase SRPK	2198	111,5	5,072793449	260g0000110.1	2198	-131,5	-5,982711556	260g0000110.1	2198	34,5	1,569608735	260g0000110.1	2198	-71,5	-3,252957234
261g0000070.1	Unknown protein	2227	33	1,4818141	261g0000070.1	2227	-98	-4,400538842	261g0000070.1	2227	76,5	3,435114504	261g0000070.1	2227	-35,5	-1,594072744
262g0000020.1	Unknown protein	5116	76	1,485535575	262g0000020.1	5116	-286,5	-5,600078186	262g0000020.1	5116	81	1,583268178	262g0000020.1	5116	-60	-1,172791243
263g0000020.1	Unknown protein	5598	96,5	1,723829939	263g0000020.1	5598	-269	-4,805287603	263g0000020.1	5598	108,5	1,938192212	263g0000020.1	5598	-140	-2,500893176
266g0000130.1	Unknown protein	1132	43	3,798586572	266g0000130.1	1132	-39,5	-3,489399293	266g0000130.1	1132	31	2,738515901	266g0000130.1	1132	-35,5	-3,136042403
267g0000030.1	Unknown protein	1573	18	1,144310235	267g0000030.1	1573	-55,5	-3,528289892	267g0000030.1	1573	38	2,415766052	267g0000030.1	1573	-27,5	-1,748251748
268g0000040.1	Unknown protein	526	17,5	3,326996198	268g0000040.1	526	-23,5	-4,467680608	268g0000040.1	526	14	2,661596958	268g0000040.1	526	-52	-9,885931559
269g0000020.1	Unknown protein	716	63,5	8,868715084	269g0000020.1	716	-22	-3,072625698	269g0000020.1	716	48,5	6,773743017	269g0000020.1	716	-7,5	-1,047486034
26g0000040.1	Regulation of nuclear pre-mRNA domain-containing protein 2	1804	72	3,99113082	26g0000040.1	1804	-81	-4,490022173	26g0000040.1	1804	26	1,441241685	26g0000040.1	1804	-22	-1,219512195
26g0000050.1	Transcription factor tau subunit sfc1	3081	50,5	1,639078221	26g0000050.1	3081	-191,5	-6,215514443	26g0000050.1	3081	37	1,200908796	26g0000050.1	3081	-56	-1,817591691
26g0000090.1	Mannan endo-1,6-alpha-mannosidase DCW1	1909	20,5	1,07386066	26g0000090.1	1909	-89	-4,662126768	26g0000090.1	1909	65,5	3,431115767	26g0000090.1	1909	-41,5	-2,173913044
26g0000180.1	Lactose permease	1888	44	2,330508475	26g0000180.1	1888	-114,5	-6,064618644	26g0000180.1	1888	33	1,747881356	26g0000180.1	1888	-49	-2,595338983
26g0000220.1	Alkaline phosphatase	2723	45	1,652589056	26g0000220.1	2723	-95,5	-3,507161219	26g0000220.1	2723	66,5	2,442159383	26g0000220.1	2723	-57	-2,093279471
26g0000240.1	Cysteine-rich secretory protein LCCL domain-containing 2	2280	26	1,140350877	26g0000240.1	2280	-150,5	-6,600877193	26g0000240.1	2280	69,5	3,048245614	26g0000240.1	2280	-27,5	-1,206140351
271g0000070.1	Presequence translocated-associated motor subunit PAM17, mitochond	850	51,5	6,058823529	271g0000070.1	850	-74	-8,705882353	271g0000070.1	850	29	3,411764706	271g0000070.1	850	-11,5	-1,352941177
271g0000110.1	Histone-lysine N-methyltransferase SMYD3	1725	71	4,115942029	271g0000110.1	1725	-63,5	-3,68115942	271g0000110.1	1725	30	1,739130435	271g0000110.1	1725	-23,5	-1,362318841
274g0000040.1	WW domain-containing adapter protein with coiled-coi	575	60	10,43478261	274g0000040.1	575	-41	-7,130434783	274g0000040.1	575	9	1,565217391	274g0000040.1	575	-10,5	-1,826086957
274g0000100.1	Sequestosome-1	215	69	32,09302326	274g0000100.1	215	-8,5	-3,953488372	274g0000100.1	215	9	4,186046512	274g0000100.1	215	-6	-2,790697674
274g0000160.1	UDP-N-acetylglucosamine--N-acetylmuramyl-(pentapeptide) pyrophosp	1264	17,5	1,384493671	274g0000160.1	1264	-91,5	-7,238924051	274g0000160.1	1264	13,5	1,068037975	274g0000160.1	1264	-48,5	-3,837025317
275g0000050.1	O-methyltransferase afvC	1549	105,5	6,810845707	275g0000050.1	1549	-49	-3,163331181	275g0000050.1	1549	18,5	1,194318915	275g0000050.1	1549	-16,5	-1,065203357
276g0000050.1	EF-hand calcium-binding domain-containing protein 5	2033	64	3,148057059	276g0000050.1	2033	-108	-5,312346286	276g0000050.1	2033	75,5	3,713723561	276g0000050.1	2033	-43,5	-2,139695032
276g0000100.1	Protein IBD2	3195	56,5	1,768388106	276g0000100.1	3195	-178,5	-5,58685446	276g0000100.1	3195	81,5	2,55086072	276g0000100.1	3195	-43	-1,345852895
277g0000060.1	4-hydroxy-3-methylbut-2-enyl diphosphate reductase	938	50	5,330490405	277g0000060.1	938	-68,5	-7,302771855	277g0000060.1	938	22	2,345415778	277g0000060.1	938	-16,5	-1,759061834
279g0000040.1	Unknown protein	1130	19,5	1,725663717	279g0000040.1	1130	-56,5	-5	279g0000040.1	1130	18,5	1,637168142	279g0000040.1	1130	-52	-4,601769912
27g0000130.1	Multiple inositol polyphosphate phosphatase 1	602	21	3,488372093	27g0000130.1	602	-14	-2,325581395	27g0000130.1	602	8,5	1,411960133	27g0000130.1	602	-12	-1,993355482
27g0000200.1	UDP-N-acetylglucosamine 1-carboxyvinyltransferase	1400	18	1,285714286	27g0000200.1	1400	-129	-9,214285714	27g0000200.1	1400	26	1,857142857	27g0000200.1	1400	-32	-2,285714286
280g0000080.1	Unknown protein	1808	41,5	2,295353982	280g0000080.1	1808	-111	-6,139380531	280g0000080.1	1808	32,5	1,797566372	280g0000080.1	1808	-22,5	-1,244469027
283g0000070.1	Cryptochrome-1	2045	121,5	5,941320293	283g0000070.1	2045	-145,5	-7,114914425	283g0000070.1	2045	45,5	2,224938875	283g0000070.1	2045	-37	-1,809290954
283g0000100.1	Death-inducer obliterator 1	2408	81,5	3,384551495	283g0000100.1	2408	-144	-5,980066445	283g0000100.1	2408	41	1,702657807	283g0000100.1	2408	-61	-2,533222591
284g0000040.1	Protein kinase dsk1	1121	25	2,23015165	284g0000040.1	1121	-59,5	-5,307760928	284g0000040.1	1121	20,5	1,828724353	284g0000040.1	1121	-29,5	-2,631578947
284g0000080.1	Uracil permease	1964	76,5	3,895112016	284g0000080.1	1964	-154,5	-7,866598778	284g0000080.1	1964	31	1,578411405	284g0000080.1	1964	-27,5	-1,400203666
285g0000020.1	Filamin A-interacting protein 1-like	2015	73,5	3,64764268	285g0000020.1	2015	-82	-4,069478908	285g0000020.1	2015	42	2,084367246	285g0000020.1	2015	-36	-1,786600496
285g0000050.1	GPI mannosyltransferase 1	1511	16,5	1,091992058	285g0000050.1	1511	-66	-4,367968233	285g0000050.1	1511	23	1,522170748	285g0000050.1	1511	-22,5	-1,489080079
285g0000110.1	Carboxylesterase A	3498	81,5	2,329902802	285g0000110.1	3498	-170,5	-4,874213837	285g0000110.1	3498	68,5	1,958261864	285g0000110.1	3498	-121,5	-3,473413379
286g0000090.1	Ubiquitin-conjugating enzyme E2 J2	1548	20,5	1,324289406	286g0000090.1	1548	-105	-6,782945736	286g0000090.1	1548	32,5	2,099483204	286g0000090.1	1548	-18	-1,162790698
289g0000020.1	Glyceraldehyde-3-phosphate dehydrogenase	1215	98,5	8,106995885	289g0000020.1	1215	-51	-4,197530864	289g0000020.1	1215	25	2,057613169	289g0000020.1	1215	-16,5	-1,358024691
289g0000090.1	40S ribosomal protein S10	784	26	3,316326531	289g0000090.1	784	-29,5	-3,762755102	289g0000090.1	784	22,5	2,869897959	289g0000090.1	784	-14,5	-1,849489796
28g0000120.1	V-type proton ATPase subunit C	1478	22,5	1,52232747	28g0000120.1	1478	-91	-6,156968877	28g0000120.1	1478	51,5	3,48443843	28g0000120.1	1478	-41,5	-2,807848444
28g0000130.1	AP-3 complex subunit sigma	630	74	11,74603175	28g0000130.1	630	-42	-6,666666667	28g0000130.1	630	27,5	4,365079365	28g0000130.1	630	-14,5	-2,301587302
28g0000140.1	Iron-sulfur cluster assembly protein CyaY	942	38	4,033970276	28g0000140.1	942	-69	-7,324840764	28g0000140.1	942	20	2,123142251	28g0000140.1	942	-19,5	-2,070063694
28g0000260.1	Pyriculol/pyriculariol biosynthesis cluster transcription factor 1	3061	67	2,188827181	28g0000260.1	3061	-262,5	-8,57562888	28g0000260.1	3061	37	1,208755309	28g0000260.1	3061	-86,5	-2,825873897
290g0000040.1	N-acetyl-gamma-glutamyl-phosphate reductase	1081	41	3,792784459	290g0000040.1	1081	-30	-2,775208141	290g0000040.1	1081	15,5	1,433857539	290g0000040.1	1081	-23	-2,127659575
292g0000010.1	Unknown protein	2266	44,5	1,963812886	292g0000010.1	2266	-91	-4,015887026	292g0000010.1	2266	50	2,206531333	292g0000010.1	2266	-26,5	-1,169461606
292g0000070.1	Unknown protein	1313	43,5	3,31302361	292g0000070.1	1313	-101,5	-7,730388424	292g0000070.1	1313	26,5	2,018278751	292g0000070.1	1313	-17	-1,294744859
293g0000020.1	Unknown protein	3523	59	1,674709055	293g0000020.1	3523	-217,5	-6,173715583	293g0000020.1	3523	72	2,043712745	293g0000020.1	3523	-58,5	-1,660516605
295g0000020.1	Unknown protein	1979	58	2,930773118	295g0000020.1	1979	-118,5	-5,987872663	295g0000020.1	1979	46	2,324406266	295g0000020.1	1979	-21,5	-1,086407276
295g0000110.1	Unknown protein	1059	20,5	1,93578848	295g0000110.1	1059	-84,5	-7,979225685	295g0000110.1	1059	11,5	1,085930123	295g0000110.1	1059	-33,5	-3,163361662
296g0000010.1	Solute carrier family 35 member C2	3941	40,5	1,027657955	296g0000010.1	3941	-254	-6,445064704	296g0000010.1	3941	89,5	2,270997209	296g0000010.1	3941	-44,5	-1,129155037
296g0000030.1	Trehalase	3024	60,5	2,000661376	296g0000030.1	3024	-221,5	-7,32473545	296g0000030.1	3024	63	2,083333333	296g0000030.1	3024	-57	-1,884920635
29g0000010.1	Na(+)/H(+) antiporter	1545	42	2,718446602	29g0000010.1	1545	-134	-8,673139159	29g0000010.1	1545	43,5	2,815533981	29g0000010.1	1545	-29	-1,877022654
29g0000030.1	Multidrug resistance protein MdtC	1366	79,5	5,819912152	29g0000030.1	1366	-79	-5,783308931	29g0000030.1	1366	14,5	1,061493411	29g0000030.1	1366	-43	-3,147877013
2g0000140.1	Acetolactate synthase, mitochondrial	2835	59,5	2,098765432	2g0000140.1	2835	-133,5	-4,708994709	2g0000140.1	2835	33,5	1,181657848	2g0000140.1	2835	-46	-1,622574956
2g0000430.1	3-hydroxyisobutyrate dehydrogenase, mitochondrial	3861	58	1,502201502	2g0000430.1	3861	-248,5	-6,436156436	2g0000430.1	3861	39,5	1,023051023	2g0000430.1	3861	-50	-1,295001295
300g0000060.1	Serine/threonine-protein kinase/endoribonuclease IRE1	595	14	2,352941177	300g0000060.1	595	-32	-5,378151261	300g0000060.1	595	8	1,344537815	300g0000060.1	595	-20	-3,361344538
300g0000100.1	Zinc transport protein ZntB	2348	31,5	1,341567291	300g0000100.1	2348	-172,5	-7,346678024	300g0000100.1	2348	68	2,896081772	300g0000100.1	2348	-28,5	-1,213798978
301g0000050.1	Unknown protein	419	55	13,12649165	301g0000050.1	419	-21,5	-5,131264917	301g0000050.1	419	29	6,92124105	301g0000050.1	419	-13	-3,102625298
303g0000060.1	Scavenger receptor cysteine-rich type 1 protein M160	560	52	9,285714286	303g0000060.1	560	-6	-1,071428571	303g0000060.1	560	33,5	5,982142857	303g0000060.1	560	-8,5	-1,517857143

304g0000030.1	Oxalate decarboxylase ARB_02208	1570	42	2,675159236	304g0000030.1	1570	-99	-6,305732484	304g0000030.1	1570	53	3,375796178	304g0000030.1	1570	-47,5	-3,025477707
306g0000010.1	Unknown protein	1650	30,5	1,848484849	306g0000010.1	1650	-201	-12,18181818	306g0000010.1	1650	44,5	2,696969697	306g0000010.1	1650	-53,5	-3,242424242
306g0000030.1	Unknown protein	2449	53	2,164148632	306g0000030.1	2449	-229	-9,35075541	306g0000030.1	2449	77	3,144140466	306g0000030.1	2449	-85,5	-3,491220907
309g0000010.1	Membrane protein	955	31	3,246073298	309g0000010.1	955	-59	-6,178010471	309g0000010.1	955	12	1,256544503	309g0000010.1	955	-13,5	-1,413612565
30g0000030.1	Sterol uptake control protein 2	1549	34,5	2,227243383	30g0000030.1	1549	-89,5	-5,77792124	30g0000030.1	1549	37,5	2,420916721	30g0000030.1	1549	-50	-3,227888961
30g0000100.1	tRNA pseudouridine synthase A	268	83,5	31,15671642	30g0000100.1	268	-17,5	-6,529850746	30g0000100.1	268	15	5,597014925	30g0000100.1	268	-15	-5,597014925
30g0000110.1	Inositol 2-dehydrogenase 1	6071	78	1,284796574	30g0000110.1	6071	-464	-7,64289244	30g0000110.1	6071	66	1,087135563	30g0000110.1	6071	-79	-1,301268325
30g0000130.1	FGGY carbohydrate kinase domain-containing protein	2207	36	1,631173539	30g0000130.1	2207	-144	-6,524694155	30g0000130.1	2207	70	3,171726325	30g0000130.1	2207	-35	-1,585863163
30g0000140.1	Polyadenylation and cleavage factor homolog 4	3696	91	2,462121212	30g0000140.1	3696	-250	-6,764069264	30g0000140.1	3696	69	1,866883117	30g0000140.1	3696	-49	-1,325757576
30g0000210.1	Cys-Gly metallodipeptidase DUG1	1376	26	1,889534884	30g0000210.1	1376	-114	-8,284883721	30g0000210.1	1376	31	2,252906977	30g0000210.1	1376	-57,5	-4,17877907
312g0000110.1	Transcriptional activator protein DAL81	1492	63,5	4,256032172	312g0000110.1	1492	-130,5	-8,746648794	312g0000110.1	1492	42,5	2,848525469	312g0000110.1	1492	-39	-2,613941019
312g0000120.1	Acetyl-CoA acetyltransferase, mitochondrial	1711	25	1,46113384	312g0000120.1	1711	-85,5	-4,997077732	312g0000120.1	1711	36	2,104032729	312g0000120.1	1711	-34	-1,987142022
313g0000080.1	DNA-directed RNA polymerase II subunit RPB1	1646	49,5	3,007290401	313g0000080.1	1646	-58,5	-3,554070474	313g0000080.1	1646	38	2,308626975	313g0000080.1	1646	-21	-1,27582017
316g0000040.1	Protein phosphatase 1 regulatory subunit 14D	704	96	13,63636364	316g0000040.1	704	-24	-3,409090909	316g0000040.1	704	8	1,136363636	316g0000040.1	704	-36,5	-5,184659091
316g0000060.1	Solute carrier family 2, facilitated glucose transporter member 2	1651	28,5	1,726226529	316g0000060.1	1651	-89,5	-5,420956996	316g0000060.1	1651	54,5	3,301029679	316g0000060.1	1651	-64,5	-3,906723198
317g0000100.1	Aromatic-amino-acid aminotransferase 1	1568	34	2,168367347	317g0000100.1	1568	-93,5	-5,963010204	317g0000100.1	1568	34	2,168367347	317g0000100.1	1568	-17	-1,084183674
318g0000050.1	Unknown protein	2303	96	4,168475901	318g0000050.1	2303	-122	-5,297438124	318g0000050.1	2303	32	1,389491967	318g0000050.1	2303	-24,5	-1,063829787
31g0000190.1	Unknown protein	1417	81	5,716302047	31g0000190.1	1417	-88,5	-6,245589273	31g0000190.1	1417	39	2,752293578	31g0000190.1	1417	-16,5	-1,164431898
31g0000200.1	Unknown protein	997	61,5	6,168505517	31g0000200.1	997	-113,5	-11,38415246	31g0000200.1	997	14,5	1,454363089	31g0000200.1	997	-27	-2,708124373
31g0000220.1	Unknown protein	1562	44,5	2,848911652	31g0000220.1	1562	-74,5	-4,769526248	31g0000220.1	1562	58	3,71318822	31g0000220.1	1562	-26,5	-1,696542894
320g0000020.1	ATP-dependent RNA helicase DBP2	2165	33	1,524249423	320g0000020.1	2165	-84,5	-3,90300231	320g0000020.1	2165	33,5	1,547344111	320g0000020.1	2165	-66,5	-3,071593534
320g0000120.1	D-amino-acid oxidase	1220	94,5	7,745901639	320g0000120.1	1220	-100	-8,196721312	320g0000120.1	1220	20	1,639344262	320g0000120.1	1220	-39	-3,196721312
321g0000070.1	tRNA dimethylallyltransferase	283	17,5	6,183745583	321g0000070.1	283	-14,5	-5,123674912	321g0000070.1	283	6	2,120141343	321g0000070.1	283	-6,5	-2,296819788
322g0000030.1	Secreted lipase ARB07186/07185	415	46	11,08433735	322g0000030.1	415	-26	-6,265060241	322g0000030.1	415	12,5	3,012048193	322g0000030.1	415	-12,5	-3,012048193
322g0000080.1	Ras-related protein Rab-1A	1035	25	2,415458937	322g0000080.1	1035	-61,5	-5,942028986	322g0000080.1	1035	16,5	1,594202899	322g0000080.1	1035	-21,5	-2,077294686
323g0000050.1	Histidine--tRNA ligase	1272	62	4,874213837	323g0000050.1	1272	-118	-9,27672956	323g0000050.1	1272	20,5	1,61163522	323g0000050.1	1272	-50	-3,93081761
323g0000080.1	Aspartate--tRNA ligase	2269	29	1,278096078	323g0000080.1	2269	-107	-4,715733803	323g0000080.1	2269	47,5	2,093433231	323g0000080.1	2269	-24	-1,057734685
324g0000010.1	Nuclear autoantigenic sperm protein	2060	41	1,990291262	324g0000010.1	2060	-114	-5,533980583	324g0000010.1	2060	25	1,213592233	324g0000010.1	2060	-76	-3,689320388
325g0000030.1	UDP-N-acetylenolpyruvoylglucosamine reductase	491	32,5	6,619144603	325g0000030.1	491	-17	-3,462321792	325g0000030.1	491	11,5	2,34215886	325g0000030.1	491	-8	-1,629327902
326g0000070.1	Unknown protein	1428	39,5	2,766106443	326g0000070.1	1428	-79,5	-5,567226891	326g0000070.1	1428	42,5	2,976190476	326g0000070.1	1428	-18	-1,260504202
326g0000090.1	Unknown protein	3079	90	2,923026957	326g0000090.1	3079	-177,5	-5,76485872	326g0000090.1	3079	58,5	1,899967522	326g0000090.1	3079	-45	-1,461513478
328g0000020.1	Glutathione-independent formaldehyde dehydrogenase	2081	28,5	1,369533878	328g0000020.1	2081	-88	-4,228736185	328g0000020.1	2081	32,5	1,561749159	328g0000020.1	2081	-71	-3,41182124
328g0000070.1	Pectate lyase L	1537	69,5	4,521795706	328g0000070.1	1537	-96,5	-6,278464541	328g0000070.1	1537	16	1,04098894	328g0000070.1	1537	-26	-1,691607027
329g0000060.1	Biotin synthase	182	90	49,45054945	329g0000060.1	182	-8	-4,395604396	329g0000060.1	182	7	3,846153846	329g0000060.1	182	-7,5	-4,120879121
32g0000080.1	Dehydrogenase orf1	1644	33,5	2,037712895	32g0000080.1	1644	-88	-5,352798054	32g0000080.1	1644	44,5	2,706812652	32g0000080.1	1644	-18,5	-1,125304136
32g0000090.1	Cysteine desulfurase IscS	1735	50	2,88184438	32g0000090.1	1735	-123	-7,089337176	32g0000090.1	1735	25,5	1,469740634	32g0000090.1	1735	-27,5	-1,585014409
32g0000170.1	Protein PA-X	3423	43	1,256208005	32g0000170.1	3423	-241,5	-7,055214724	32g0000170.1	3423	61,5	1,796669588	32g0000170.1	3423	-76,5	-2,234881683
32g0000230.1	Cytochrome c heme lyase	1152	26,5	2,300347222	32g0000230.1	1152	-85,5	-7,421875	32g0000230.1	1152	18	1,5625	32g0000230.1	1152	-16	-1,388888889
330g0000060.1	3-dehydroquinate synthase	2617	49	1,872372946	330g0000060.1	2617	-155	-5,922812381	330g0000060.1	2617	72,5	2,770347726	330g0000060.1	2617	-57	-2,178066488
332g0000040.1	Unknown protein	1475	65	4,406779661	332g0000040.1	1475	-68	-4,610169492	332g0000040.1	1475	29	1,966101695	332g0000040.1	1475	-16	-1,084745763
333g0000010.1	Ubiquitin-associated protein 2	1200	62	5,166666667	333g0000010.1	1200	-57	-4,75	333g0000010.1	1200	26	2,166666667	333g0000010.1	1200	-20,5	-1,708333333
333g0000030.1	CXXC motif containing zinc binding protein	833	10,5	1,260504202	333g0000030.1	833	-36,5	-4,381752701	333g0000030.1	833	37	4,441776711	333g0000030.1	833	-27	-3,241296519
336g0000020.1	Unknown protein	934	16,5	1,766595289	336g0000020.1	934	-27	-2,890792291	336g0000020.1	934	20	2,141327623	336g0000020.1	934	-15	-1,605995717
336g0000050.1	Unknown protein	1182	16,5	1,395939086	336g0000050.1	1182	-38	-3,214890017	336g0000050.1	1182	40,5	3,426395939	336g0000050.1	1182	-32,5	-2,749576988
337g0000020.1	Translation initiation factor IF-2	1898	33,5	1,765015806	337g0000020.1	1898	-111,5	-5,874604847	337g0000020.1	1898	29	1,527924131	337g0000020.1	1898	-22	-1,159114858
339g0000040.1	Unknown protein	1229	49,5	4,027664768	339g0000040.1	1229	-130	-10,57770545	339g0000040.1	1229	27	2,196908055	339g0000040.1	1229	-12,5	-1,017087063
33g0000030.1	Copper-transporting ATPase 2	473	46,5	9,830866808	33g0000030.1	473	-30	-6,342494715	33g0000030.1	473	17	3,594080338	33g0000030.1	473	-12,5	-2,642706131
33g0000040.1	Stomatin-like protein 2, mitochondrial	1458	33	2,263374486	33g0000040.1	1458	-85,5	-5,864197531	33g0000040.1	1458	31,5	2,160493827	33g0000040.1	1458	-35	-2,400548697
33g0000150.1	Maltose permease MAL61	2231	25,5	1,142985208	33g0000150.1	2231	-117,5	-5,266696549	33g0000150.1	2231	42	1,882563873	33g0000150.1	2231	-34,5	-1,546391753
33g0000160.1	Inosine-5'-monophosphate dehydrogenase	800	41	5,125	33g0000160.1	800	-82,5	-10,3125	33g0000160.1	800	13,5	1,6875	33g0000160.1	800	-22,5	-2,8125
33g0000190.1	N amino acid transport system protein	1533	55,5	3,620352251	33g0000190.1	1533	-78	-5,088062622	33g0000190.1	1533	63	4,109589041	33g0000190.1	1533	-18	-1,174168298
33g0000200.1	Sulfite efflux pump SSU1	1366	17,5	1,281112738	33g0000200.1	1366	-62,5	-4,575402635	33g0000200.1	1366	38,5	2,818448023	33g0000200.1	1366	-19	-1,390922401
33g0000210.1	Peptidyl-tRNA hydrolase	603	55	9,12106136	33g0000210.1	603	-19,5	-3,233830846	33g0000210.1	603	19	3,150912106	33g0000210.1	603	-13,5	-2,23880597
343g0000060.1	Unknown protein	3019	34	1,126200729	343g0000060.1	3019	-139,5	-4,620735343	343g0000060.1	3019	80	2,649884068	343g0000060.1	3019	-36	-1,19244783
343g0000100.1	Unknown protein	339	23	6,784660767	343g0000100.1	339	-35,5	-10,4719764	343g0000100.1	339	8,5	2,507374631	343g0000100.1	339	-6,5	-1,91740413
344g0000030.1	tRNA1(Val) (adenine(37)-N6)-methyltransferase	1224	46	3,758169935	344g0000030.1	1224	-90	-7,352941177	344g0000030.1	1224	19	1,552287582	344g0000030.1	1224	-13	-1,062091503
345g0000130.1	Ferric reductase transmembrane component 3	1895	65	3,430079156	345g0000130.1	1895	-132	-6,965699208	345g0000130.1	1895	29,5	1,556728232	345g0000130.1	1895	-51	-2,691292876
348g0000040.1	Unknown protein	674	58,5	8,679525223	348g0000040.1	674	-39,5	-5,860534125	348g0000040.1	674	26,5	3,931750742	348g0000040.1	674	-18	-2,670623145
348g0000070.1	Unknown protein	744	40,5	5,443548387	348g0000070.1	744	-50,5	-6,787634409	348g0000070.1	744	15	2,016129032	348g0000070.1	744	-19	-2,553763441
34g0000050.1	Unknown protein	1702	77	4,524089307	34g0000050.1	1702	-124	-7,285546416	34g0000050.1	1702	60,5	3,554641598	34g0000050.1	1702	-41	-2,40893067
34g0000110.1	Unknown protein	290	18,5	6,379310345	34g0000110.1	290	-29	-10	34g0000110.1	290	6	2,068965517	34g0000110.1	290	-4,5	-1,551724138
350g0000040.1	Maintenance of mitochondrial morphology protein 1	2471	36,5	1,477134763	350g0000040.1	2471	-194	-7,85107244	350g0000040.1	2471	31,5	1,274787535	350g0000040.1	2471	-52,5	-2,124645892

353g0000110.1	Unknown protein	1423	145	10,18973999	353g0000110.1	1423	-50,5	-3,548840478	353g0000110.1	1423	17,5	1,229796205	353g0000110.1	1423	-16,5	-1,159522136
354g0000030.1	Cytochrome P450 monooxygenase eqxH	1807	45	2,49031544	354g0000030.1	1807	-153	-8,467072496	354g0000030.1	1807	23	1,272827892	354g0000030.1	1807	-132,5	-7,332595462
355g0000020.1	Envelope glycoprotein D	578	69	11,93771626	355g0000020.1	578	-37,5	-6,487889273	355g0000020.1	578	9,5	1,643598616	355g0000020.1	578	-18,5	-3,200692042
356g0000020.1	Ferric uptake regulation protein	1012	41	4,051383399	356g0000020.1	1012	-79	-7,806324111	356g0000020.1	1012	13	1,28458498	356g0000020.1	1012	-23,5	-2,322134387
356g0000090.1	D-aminoacyl-tRNA deacylase	1621	56,5	3,485502776	356g0000090.1	1621	-67	-4,13325108	356g0000090.1	1621	22	1,357186922	356g0000090.1	1621	-37,5	-2,313386798
357g0000010.1	Unknown protein	3379	58	1,716484167	357g0000010.1	3379	-139	-4,11364309	357g0000010.1	3379	59	1,746078722	357g0000010.1	3379	-35,5	-1,050606688
358g0000070.1	RNA polymerase-associated protein RapA	819	48	5,860805861	358g0000070.1	819	-61,5	-7,509157509	358g0000070.1	819	9,5	1,15995116	358g0000070.1	819	-31	-3,785103785
359g0000110.1	Dual specificity protein phosphatase 15	869	38	4,372842348	359g0000110.1	869	-46,5	-5,350978136	359g0000110.1	869	41	4,718066743	359g0000110.1	869	-9	-1,035673188
35g0000020.1	2-iminobutanoate/2-iminopropanoate deaminase	853	43	5,041031653	35g0000020.1	853	-51	-5,978898007	35g0000020.1	853	30	3,516998828	35g0000020.1	853	-38,5	-4,513481829
35g0000190.1	MFS transporter prlL	1966	51,5	2,619532045	35g0000190.1	1966	-157	-7,985757884	35g0000190.1	1966	37	1,881993896	35g0000190.1	1966	-39,5	-2,009155646
35g0000210.1	Trichothecene efflux pump TRI12	1948	43,5	2,233059548	35g0000210.1	1948	-120,5	-6,185831622	35g0000210.1	1948	37	1,899383984	35g0000210.1	1948	-64,5	-3,311088296
363g0000020.1	MFS transporter prlL	1819	30,5	1,676745465	363g0000020.1	1819	-43,5	-2,391423859	363g0000020.1	1819	59,5	3,271028037	363g0000020.1	1819	-35	-1,92413414
364g0000050.1	UvrABC system protein B	1720	84	4,88372093	364g0000050.1	1720	-97,5	-5,668604651	364g0000050.1	1720	37,5	2,180232558	364g0000050.1	1720	-28	-1,627906977
364g0000070.1	Aryl hydrocarbon receptor nuclear translocator-like protein 1	2699	52	1,926639496	364g0000070.1	2699	-125	-4,631344943	364g0000070.1	2699	54,5	2,019266395	364g0000070.1	2699	-35,5	-1,315301964
367g0000030.1	Unknown protein	2867	78,5	2,738053715	367g0000030.1	2867	-225,5	-7,865364493	367g0000030.1	2867	32,5	1,133589118	367g0000030.1	2867	-42,5	-1,482385769
369g0000010.1	ATP-dependent zinc metalloprotease FtsH	7150	82,5	1,153846154	369g0000010.1	7150	-303,5	-4,244755245	369g0000010.1	7150	166,5	2,328671329	369g0000010.1	7150	-129,5	-1,811188811
36g0000090.1	Transcription activator of gluconeogenesis CHGG_09150	4524	47,5	1,049955791	36g0000090.1	4524	-251	-5,548187445	36g0000090.1	4524	57,5	1,270999116	36g0000090.1	4524	-50	-1,105216623
36g0000150.1	Serine/threonine-protein kinase 32A	3222	35,5	1,101800124	36g0000150.1	3222	-131	-4,065797641	36g0000150.1	3222	52,5	1,629422719	36g0000150.1	3222	-36,5	-1,132836747
36g0000170.1	BSD domain-containing protein 1	2128	57,5	2,702067669	36g0000170.1	2128	-102	-4,793233083	36g0000170.1	2128	56,5	2,655075188	36g0000170.1	2128	-62	-2,913533835
36g0000260.1	Carboxy-cis,cis-muconate cyclase	1511	36,5	2,415618796	36g0000260.1	1511	-109,5	-7,246856387	36g0000260.1	1511	17,5	1,158173395	36g0000260.1	1511	-25	-1,654533422
36g0000270.1	Catechol 1,2-dioxygenase	1213	14,5	1,195383347	36g0000270.1	1213	-53	-4,369332234	36g0000270.1	1213	37	3,050288541	36g0000270.1	1213	-20	-1,648804617
370g0000040.1	Lipase chaperone	719	43	5,980528512	370g0000040.1	719	-25,5	-3,54659249	370g0000040.1	719	27	3,755215577	370g0000040.1	719	-7,5	-1,043115438
373g0000040.1	Serine/threonine-protein kinase csk1	2650	45	1,698113208	373g0000040.1	2650	-157,5	-5,943396226	373g0000040.1	2650	45	1,698113208	373g0000040.1	2650	-44,5	-1,679245283
375g0000040.1	Protein phosphatase Slingshot homolog 3	677	28,5	4,209748892	375g0000040.1	677	-55,5	-8,197932053	375g0000040.1	677	20	2,954209749	375g0000040.1	677	-24	-3,545051699
375g0000070.1	Non-structural maintenance of chromosome element 4	1433	16,5	1,151430565	375g0000070.1	1433	-58	-4,047452896	375g0000070.1	1433	15	1,046755059	375g0000070.1	1433	-48,5	-3,384508025
377g0000030.1	Unknown protein	581	17,5	3,012048193	377g0000030.1	581	-36	-6,196213425	377g0000030.1	581	7,5	1,290877797	377g0000030.1	581	-23	-3,958691911
377g0000060.1	Unknown protein	1715	23,5	1,370262391	377g0000060.1	1715	-114	-6,647230321	377g0000060.1	1715	18	1,049562682	377g0000060.1	1715	-80	-4,664723032
377g0000070.1	Unknown protein	2990	65,5	2,190635452	377g0000070.1	2990	-158	-5,284280937	377g0000070.1	2990	44	1,471571906	377g0000070.1	2990	-63	-2,107023411
377g0000080.1	Unknown protein	1002	23	2,295409182	377g0000080.1	1002	-66,5	-6,636726547	377g0000080.1	1002	30,5	3,043912176	377g0000080.1	1002	-37	-3,692614771
378g0000030.1	Cis-aconitate decarboxylase	540	14	2,592592593	378g0000030.1	540	-33,5	-6,203703704	378g0000030.1	540	8,5	1,574074074	378g0000030.1	540	-25	-4,62962963
379g0000030.1	Unknown protein	2527	30	1,187178473	379g0000030.1	2527	-189,5	-7,499010685	379g0000030.1	2527	49	1,939058172	379g0000030.1	2527	-45,5	-1,800554017
379g0000090.1	Unknown protein	1774	24,5	1,381059752	379g0000090.1	1774	-132	-7,440811725	379g0000090.1	1774	37	2,085682074	379g0000090.1	1774	-46	-2,593010147
37g0000100.1	Serine-aspartate repeat-containing protein C	782	11,5	1,470588235	37g0000100.1	782	-44,5	-5,690537084	37g0000100.1	782	8	1,023017903	37g0000100.1	782	-54,5	-6,969309463
37g0000110.1	NADH dehydrogenase [ubiquinone] 1 alpha subcomplex subunit 8	1035	72	6,956521739	37g0000110.1	1035	-46	-4,444444444	37g0000110.1	1035	26	2,512077295	37g0000110.1	1035	-24,5	-2,367149759
37g0000140.1	Glycine dehydrogenase (decarboxylating)	3636	64	1,760176018	37g0000140.1	3636	-177,5	-4,881738174	37g0000140.1	3636	76	2,090209021	37g0000140.1	3636	-106,5	-2,929042904
37g0000350.1	Major facilitator-type transporter ecdD	1775	35	1,971830986	37g0000350.1	1775	-119,5	-6,732394366	37g0000350.1	1775	21,5	1,211267606	37g0000350.1	1775	-47,5	-2,676056338
382g0000020.1	Unknown protein	2361	42,5	1,80008471	382g0000020.1	2361	-107	-4,531977975	382g0000020.1	2361	75	3,176620076	382g0000020.1	2361	-24	-1,016518424
382g0000080.1	Unknown protein	1315	70,5	5,36121673	382g0000080.1	1315	-103,5	-7,870722434	382g0000080.1	1315	18	1,368821293	382g0000080.1	1315	-20	-1,520912548
383g0000090.1	Unknown protein	351	6,5	1,851851852	383g0000090.1	351	-23	-6,552706553	383g0000090.1	351	7	1,994301994	383g0000090.1	351	-5,5	-1,566951567
384g0000080.1	Unknown protein	1342	16,5	1,229508197	384g0000080.1	1342	-111	-8,27123696	384g0000080.1	1342	24,5	1,825633383	384g0000080.1	1342	-38	-2,831594635
385g0000050.1	Unknown protein	1447	16,5	1,140290256	385g0000050.1	1447	-92	-6,357982032	385g0000050.1	1447	21,5	1,485832757	385g0000050.1	1447	-63,5	-4,388389772
385g0000070.1	Unknown protein	1388	40	2,88184438	385g0000070.1	1388	-61	-4,39481268	385g0000070.1	1388	49,5	3,566282421	385g0000070.1	1388	-14	-1,008645533
386g0000070.1	Unknown protein	221	92	41,62895928	386g0000070.1	221	-13,5	-6,108597285	386g0000070.1	221	8	3,619909502	386g0000070.1	221	-10,5	-4,751131222
387g0000020.1	Sporozoite surface protein 2	3637	69,5	1,91091559	387g0000020.1	3637	-244	-6,708825956	387g0000020.1	3637	68,5	1,883420401	387g0000020.1	3637	-50	-1,374759417
388g0000020.1	Nitrogen assimilation transcription factor nit-4	2880	87,5	3,038194444	388g0000020.1	2880	-197	-6,840277778	388g0000020.1	2880	40,5	1,40625	388g0000020.1	2880	-89,5	-3,107638889
38g0000140.1	Unknown protein	1126	32	2,841918295	38g0000140.1	1126	-96	-8,525754885	38g0000140.1	1126	30	2,664298401	38g0000140.1	1126	-32,5	-2,886323268
38g0000160.1	Unknown protein	425	29	6,823529412	38g0000160.1	425	-15,5	-3,647058824	38g0000160.1	425	10	2,352941177	38g0000160.1	425	-19	-4,470588235
38g0000200.1	Unknown protein	1832	24,5	1,337336245	38g0000200.1	1832	-114,5	-6,25	38g0000200.1	1832	86	4,694323144	38g0000200.1	1832	-66,5	-3,629912664
38g0000210.1	Unknown protein	1987	58,5	2,94413689	38g0000210.1	1987	-171,5	-8,631102164	38g0000210.1	1987	27	1,358832411	38g0000210.1	1987	-21	-1,056869653
38g0000230.1	Unknown protein	1721	83	4,822777455	38g0000230.1	1721	-96,5	-5,607205113	38g0000230.1	1721	25,5	1,481696688	38g0000230.1	1721	-28	-1,626961069
391g0000060.1	Unknown protein	1615	20	1,238390093	391g0000060.1	1615	-112	-6,93498452	391g0000060.1	1615	29	1,795665635	391g0000060.1	1615	-41	-2,53869969
391g0000070.1	Unknown protein	1118	40,5	3,62254025	391g0000070.1	1118	-24	-2,146690519	391g0000070.1	1118	18,5	1,654740608	391g0000070.1	1118	-28	-2,504472272
392g0000120.1	Retrovirus-related Pol polyprotein from type-1 retrotransposable eleme	2627	59,5	2,264940997	392g0000120.1	2627	-189	-7,194518462	392g0000120.1	2627	57,5	2,188808527	392g0000120.1	2627	-69	-2,626570232
393g0000040.1	Small nuclear ribonucleoprotein Sm D3	523	11	2,103250478	393g0000040.1	523	-20,5	-3,919694073	393g0000040.1	523	30	5,736137667	393g0000040.1	523	-35,5	-6,787762906
393g0000060.1	Protein LOT5	1051	129	12,27402474	393g0000060.1	1051	-90,5	-8,610846813	393g0000060.1	1051	15,5	1,474785918	393g0000060.1	1051	-33,5	-3,187440533
396g0000050.1	Eukaryotic translation initiation factor 3 subunit B	2226	40	1,796945193	396g0000050.1	2226	-115,5	-5,188679245	396g0000050.1	2226	23	1,033243486	396g0000050.1	2226	-58	-2,60557053
398g0000070.1	Unknown protein	2232	26	1,164874552	398g0000070.1	2232	-93	-4,166666667	398g0000070.1	2232	52	2,329749104	398g0000070.1	2232	-25,5	-1,142473118
39g0000010.1	Proprotein convertase subtilisin/kexin type 7	3274	66,5	2,031154551	39g0000010.1	3274	-153	-4,673182651	39g0000010.1	3274	68	2,076970067	39g0000010.1	3274	-38	-1,160659743
39g0000050.1	Protein MNN4	1036	47	4,536679537	39g0000050.1	1036	-30	-2,895752896	39g0000050.1	1036	16	1,544401544	39g0000050.1	1036	-38	-3,667953668
39g0000090.1	30S ribosomal protein S17	1339	16	1,194921583	39g0000090.1	1339	-33	-2,464525766	39g0000090.1	1339	37	2,763256161	39g0000090.1	1339	-26	-1,941747573
39g0000130.1	Elongation factor 4	1079	74	6,858202039	39g0000130.1	1079	-49	-4,541241891	39g0000130.1	1079	28,5	2,641334569	39g0000130.1	1079	-12	-1,112140871
3g0000080.1	Siderophore iron transporter mirB	1954	55,5	2,840327533	3g0000080.1	1954	-137,5	-7,036847492	3g0000080.1	1954	28	1,432958035	3g0000080.1	1954	-57	-2,917093142

3g0000240.1	Annexin A7	2201	44,5	2,021808269	3g0000240.1	2201	-141	-6,40617901	3g0000240.1	2201	48	2,180826897	3g0000240.1	2201	-52,5	-2,385279418
3g0000410.1	Golgi-specific brefeldin A-resistance guanine nucleotide exchange factor	641	47,5	7,410296412	3g0000410.1	641	-40,5	-6,31825273	3g0000410.1	641	10	1,560062403	3g0000410.1	641	-24	-3,744149766
400g0000050.1	WD repeat-containing protein 5B	1424	15,5	1,088483146	400g0000050.1	1424	-98	-6,882022472	400g0000050.1	1424	47,5	3,335674157	400g0000050.1	1424	-28,5	-2,001404494
404g0000070.1	Unknown protein	585	34	5,811965812	404g0000070.1	585	-18,5	-3,162393162	404g0000070.1	585	21	3,58974359	404g0000070.1	585	-20	-3,418803419
404g0000100.1	Unknown protein	3227	64,5	1,998760459	404g0000100.1	3227	-146	-4,524325999	404g0000100.1	3227	53,5	1,657886582	404g0000100.1	3227	-97,5	-3,021382089
406g0000020.1	Apoptosis regulator Bcl-2	2255	70	3,10421286	406g0000020.1	2255	-162,5	-7,206208426	406g0000020.1	2255	41,5	1,840354767	406g0000020.1	2255	-71	-3,148558758
408g0000080.1	Translationally-controlled tumor protein homolog	668	62,5	9,356287425	408g0000080.1	668	-44	-6,586826347	408g0000080.1	668	8,5	1,27245509	408g0000080.1	668	-60,5	-9,056886228
409g0000040.1	Unknown protein	1336	38,5	2,881736527	409g0000040.1	1336	-61,5	-4,603293413	409g0000040.1	1336	38,5	2,881736527	409g0000040.1	1336	-37	-2,769461078
40g0000140.1	UPF0658 Golgi apparatus membrane protein C23H3.04	2164	52,5	2,426062847	40g0000140.1	2164	-120	-5,545286507	40g0000140.1	2164	33	1,524953789	40g0000140.1	2164	-23,5	-1,085951941
40g0000240.1	Nuclear movement protein nudC	2899	45	1,5522594	40g0000240.1	2899	-180,5	-6,226284926	40g0000240.1	2899	45,5	1,569506727	40g0000240.1	2899	-39	-1,34529148
410g0000020.1	Cutinase transcription factor 1 alpha	2475	54,5	2,202020202	410g0000020.1	2475	-224	-9,050505051	410g0000020.1	2475	25	1,01010101	410g0000020.1	2475	-99	-4
410g0000040.1	5'-hydroxyaverantin dehydrogenase	1348	43	3,189910979	410g0000040.1	1348	-64	-4,747774481	410g0000040.1	1348	47,5	3,523738872	410g0000040.1	1348	-39,5	-2,930267062
417g0000050.1	3-oxoacyl-[acyl-carrier-protein] synthase 3	1233	77,5	6,285482563	417g0000050.1	1233	-90,5	-7,339821573	417g0000050.1	1233	20	1,622060016	417g0000050.1	1233	-31,5	-2,554744526
418g0000040.1	Protein kish-A	549	11	2,003642987	418g0000040.1	549	-31	-5,646630237	418g0000040.1	549	7,5	1,366120219	418g0000040.1	549	-7	-1,275045537
419g0000080.1	30S ribosomal protein S15	1015	39,5	3,891625616	419g0000080.1	1015	-60,5	-5,960591133	419g0000080.1	1015	25	2,463054187	419g0000080.1	1015	-16,5	-1,625615764
41g0000130.1	Unknown protein	4358	49	1,124368977	41g0000130.1	4358	-281	-6,447911886	41g0000130.1	4358	64,5	1,480036714	41g0000130.1	4358	-66,5	-1,525929325
41g0000180.1	Unknown protein	3221	57,5	1,785159888	41g0000180.1	3221	-236,5	-7,342440236	41g0000180.1	3221	39	1,210804098	41g0000180.1	3221	-79,5	-2,468177585
420g0000020.1	Integral membrane protein sed5	1802	57	3,163152053	420g0000020.1	1802	-53,5	-2,968923418	420g0000020.1	1802	35,5	1,970033296	420g0000020.1	1802	-61,5	-3,412874584
420g0000030.1	D-galacturonate reductase	1248	21	1,682692308	420g0000030.1	1248	-38,5	-3,084935897	420g0000030.1	1248	18	1,442307692	420g0000030.1	1248	-20	-1,602564103
422g0000050.1	ADP-ribosylation factor	1540	31,5	2,045454546	422g0000050.1	1540	-50,5	-3,279220779	422g0000050.1	1540	46	2,987012987	422g0000050.1	1540	-16	-1,038961039
424g0000020.1	Rho GTPase-activating protein 21	4856	50,5	1,039950577	424g0000020.1	4856	-360	-7,413509061	424g0000020.1	4856	69,5	1,43121911	424g0000020.1	4856	-126,5	-2,605024712
428g0000040.1	mRNA (2'-O-methyladenosine-N(6)-)-methyltransferase	266	58,5	21,9924812	428g0000040.1	266	-19	-7,142857143	428g0000040.1	266	4,5	1,691729323	428g0000040.1	266	-3	-1,127819549
42g0000030.1	Unknown protein	755	59	7,814569536	42g0000030.1	755	-74	-9,801324503	42g0000030.1	755	12,5	1,655629139	42g0000030.1	755	-47	-6,225165563
430g0000010.1	Cell wall integrity protein scw1	1526	16,5	1,081258191	430g0000010.1	1526	-100	-6,553079948	430g0000010.1	1526	37	2,424639581	430g0000010.1	1526	-30	-1,965923984
430g0000020.1	5'-hydroxyaverantin dehydrogenase	1252	23	1,837060703	430g0000020.1	1252	-69	-5,511182109	430g0000020.1	1252	32,5	2,595846645	430g0000020.1	1252	-34,5	-2,755591054
433g0000110.1	L-rhamnono-gamma-lactonase	1808	33	1,825221239	433g0000110.1	1808	-131	-7,245575221	433g0000110.1	1808	26,5	1,465707965	433g0000110.1	1808	-33	-1,825221239
434g0000010.1	Unknown protein	1164	31	2,663230241	434g0000010.1	1164	-31,5	-2,706185567	434g0000010.1	1164	38,5	3,307560138	434g0000010.1	1164	-38	-3,264604811
438g0000060.1	V-type proton ATPase subunit H	1806	62,5	3,4606866	438g0000060.1	1806	-123,5	-6,838316722	438g0000060.1	1806	26,5	1,467331119	438g0000060.1	1806	-21	-1,162790698
43g0000070.1	Secondary metabolism regulator laeA	838	23,5	2,804295943	43g0000070.1	838	-10	-1,193317422	43g0000070.1	838	25	2,983293556	43g0000070.1	838	-8,5	-1,014319809
43g0000180.1	Zinc finger protein 76	3731	39	1,045296167	43g0000180.1	3731	-238,5	-6,3923881	43g0000180.1	3731	47	1,259715894	43g0000180.1	3731	-62	-1,661752881
441g0000020.1	Unknown protein	1061	56,5	5,325164939	441g0000020.1	1061	-63,5	-5,984919887	441g0000020.1	1061	16	1,50801131	441g0000020.1	1061	-25	-2,356267672
441g0000030.1	Unknown protein	5676	57	1,00422833	441g0000030.1	5676	-327,5	-5,769908386	441g0000030.1	5676	138,5	2,440098661	441g0000030.1	5676	-133,5	-2,352008457
445g0000070.1	Glutaredoxin-like protein C5orf63 homolog	383	7	1,82767624	445g0000070.1	383	-27,5	-7,180156658	445g0000070.1	383	9	2,349869452	445g0000070.1	383	-12,5	-3,263707572
446g0000090.1	Unknown protein	1341	76,5	5,704697987	446g0000090.1	1341	-79,5	-5,928411633	446g0000090.1	1341	25	1,864280388	446g0000090.1	1341	-32	-2,386278896
447g0000110.1	MFS transporter prlL	2024	97,5	4,817193676	447g0000110.1	2024	-149,5	-7,386363636	447g0000110.1	2024	22,5	1,111660079	447g0000110.1	2024	-30,5	-1,506916996
447g0000140.1	Ubiquinone biosynthesis O-methyltransferase	907	21,5	2,37045204	447g0000140.1	907	-62,5	-6,890848953	447g0000140.1	907	11	1,212789416	447g0000140.1	907	-40,5	-4,465270121
448g0000020.1	Altered inheritance of mitochondria protein 32	1364	145	10,63049853	448g0000020.1	1364	-58	-4,252199414	448g0000020.1	1364	47	3,445747801	448g0000020.1	1364	-15	-1,099706745
448g0000030.1	Phospholipid-transporting ATPase IB	4521	82,5	1,824817518	448g0000030.1	4521	-361,5	-7,99601858	448g0000030.1	4521	132	2,919708029	448g0000030.1	4521	-50	-1,105950011
450g0000050.1	Unknown protein	1212	48,5	4,001650165	450g0000050.1	1212	-71	-5,858085809	450g0000050.1	1212	18	1,485148515	450g0000050.1	1212	-28	-2,310231023
452g0000100.1	Unknown protein	494	55,5	11,23481781	452g0000100.1	494	-23,5	-4,75708502	452g0000100.1	494	11	2,226720648	452g0000100.1	494	-6,5	-1,315789474
453g0000030.1	Unknown protein	939	55	5,857294995	453g0000030.1	939	-39,5	-4,206602769	453g0000030.1	939	17	1,810436635	453g0000030.1	939	-19,5	-2,076677316
453g0000080.1	Unknown protein	3138	34	1,083492671	453g0000080.1	3138	-236	-7,520713831	453g0000080.1	3138	48	1,529636711	453g0000080.1	3138	-68	-2,166985341
456g0000020.1	Endoribonuclease SymE	535	5,5	1,028037383	456g0000020.1	535	-35	-6,542056075	456g0000020.1	535	23,5	4,392523365	456g0000020.1	535	-25	-4,672897196
457g0000060.1	E3 ubiquitin-protein ligase RNF144A	2218	38,5	1,735798016	457g0000060.1	2218	-179	-8,070333634	457g0000060.1	2218	27	1,217312895	457g0000060.1	2218	-24,5	-1,104598738
457g0000090.1	Cytochrome P450 monooxygenase aclL	1941	42	2,163833076	457g0000090.1	1941	-84	-4,327666152	457g0000090.1	1941	55	2,833590933	457g0000090.1	1941	-43	-2,215352911
458g0000090.1	Unknown protein	1823	36	1,974766868	458g0000090.1	1823	-174,5	-9,572133845	458g0000090.1	1823	26	1,426220516	458g0000090.1	1823	-35,5	-1,94733955
459g0000020.1	Vacuolar protein sorting-associated protein 13a	3146	34,5	1,096630642	459g0000020.1	3146	-206,5	-6,563890655	459g0000020.1	3146	71,5	2,272727273	459g0000020.1	3146	-32,5	-1,033057851
459g0000040.1	Beta-lactamase	227	77	33,92070485	459g0000040.1	227	-20,5	-9,030837004	459g0000040.1	227	2,5	1,101321586	459g0000040.1	227	-12,5	-5,50660793
45g0000020.1	Unknown protein	1890	30	1,587301587	45g0000020.1	1890	-102,5	-5,423280423	45g0000020.1	1890	38	2,010582011	45g0000020.1	1890	-26,5	-1,402116402
45g0000050.1	Unknown protein	2875	62,5	2,173913044	45g0000050.1	2875	-211	-7,339130435	45g0000050.1	2875	29	1,008695652	45g0000050.1	2875	-31,5	-1,095652174
45g0000120.1	Unknown protein	1269	47	3,703703704	45g0000120.1	1269	-78	-6,146572104	45g0000120.1	1269	28	2,206461781	45g0000120.1	1269	-37	-2,915681639
461g0000060.1	Histidinol dehydrogenase	1925	67,5	3,506493507	461g0000060.1	1925	-138,5	-7,194805195	461g0000060.1	1925	27	1,402597403	461g0000060.1	1925	-60,5	-3,142857143
462g0000070.1	Unknown protein	1384	60	4,335260116	462g0000070.1	1384	-82,5	-5,960982659	462g0000070.1	1384	47,5	3,432080925	462g0000070.1	1384	-44,5	-3,215317919
465g0000040.1	Unknown protein	1463	47	3,212576897	465g0000040.1	1463	-83,5	-5,707450444	465g0000040.1	1463	34,5	2,358168148	465g0000040.1	1463	-37,5	-2,563226247
467g0000010.1	Unknown protein	1982	46,5	2,346115035	467g0000010.1	1982	-108,5	-5,474268416	467g0000010.1	1982	59	2,97679112	467g0000010.1	1982	-23,5	-1,185671039
468g0000060.1	Serotonin N-acetyltransferase 1, chloroplastic	629	35,5	5,643879173	468g0000060.1	629	-37	-5,882352941	468g0000060.1	629	34,5	5,484896661	468g0000060.1	629	-19,5	-3,100158983
46g0000050.1	Oxygen-dependent choline dehydrogenase	1294	18,5	1,429675425	46g0000050.1	1294	-27	-2,086553323	46g0000050.1	1294	58	4,482225657	46g0000050.1	1294	-52	-4,018547141
473g0000010.1	Unknown protein	1018	33,5	3,290766208	473g0000010.1	1018	-46	-4,518664047	473g0000010.1	1018	25	2,455795678	473g0000010.1	1018	-12	-1,178781925
473g0000030.1	Unknown protein	1183	24	2,02874049	473g0000030.1	1183	-66,5	-5,621301775	473g0000030.1	1183	23	1,944209637	473g0000030.1	1183	-18	-1,521555368
474g0000050.1	Aldose 1-epimerase	1028	21	2,042801556	474g0000050.1	1028	-51	-4,961089494	474g0000050.1	1028	37,5	3,647859922	474g0000050.1	1028	-17	-1,653696498
479g0000020.1	Unknown protein	827	20	2,418379686	479g0000020.1	827	-53,5	-6,469165659	479g0000020.1	827	40	4,836759371	479g0000020.1	827	-12	-1,451027811
47g0000030.1	Ubiquitin-conjugating enzyme E2 2	574	15,5	2,700348432	47g0000030.1	574	-43	-7,491289199	47g0000030.1	574	21,5	3,745644599	47g0000030.1	574	-23,5	-4,094076655

47g0000070.1	NADH-ubiquinone oxidoreductase 29.9 kDa subunit, mitochondria	3613	44,5	1,231663438	47g0000070.1	3613	-182	-5,037365071	47g0000070.1	3613	40	1,107113202	47g0000070.1	3613	-70,5	-1,951287019
481g0000030.1	Unknown protein	1523	77,5	5,08864084	481g0000030.1	1523	-68,5	-4,497701904	481g0000030.1	1523	35,5	2,330925804	481g0000030.1	1523	-32	-2,101116218
483g0000070.1	Unknown protein	6138	755	12,30042359	483g0000070.1	6138	-366	-5,96285435	483g0000070.1	6138	108,5	1,767676768	483g0000070.1	6138	-73,5	-1,197458456
483g0000080.1	Unknown protein	911	30	3,293084523	483g0000080.1	911	-31	-3,402854007	483g0000080.1	911	11	1,207464325	483g0000080.1	911	-21,5	-2,360043908
485g0000030.1	DNA mismatch repair protein MutL	510	33	6,470588235	485g0000030.1	510	-35,5	-6,960784314	485g0000030.1	510	21,5	4,215686275	485g0000030.1	510	-9,5	-1,862745098
487g0000050.1	Unknown protein	1071	33,5	3,127917834	487g0000050.1	1071	-105,5	-9,850606909	487g0000050.1	1071	12,5	1,16713352	487g0000050.1	1071	-26	-2,427637722
488g0000060.1	RNA-binding motif protein, X-linked 2	1076	27,5	2,555762082	488g0000060.1	1076	-57,5	-5,343866171	488g0000060.1	1076	46	4,275092937	488g0000060.1	1076	-14	-1,301115242
48g0000010.1	Unknown protein	978	43	4,396728016	48g0000010.1	978	-52,5	-5,36809816	48g0000010.1	978	26	2,658486708	48g0000010.1	978	-20	-2,044989775
48g0000210.1	Unknown protein	1728	21,5	1,244212963	48g0000210.1	1728	-116	-6,712962963	48g0000210.1	1728	21	1,215277778	48g0000210.1	1728	-30	-1,736111111
491g0000060.1	Triose phosphate/phosphate translocator, chloroplastic	1382	236	17,07670043	491g0000060.1	1382	-93,5	-6,765557164	491g0000060.1	1382	15,5	1,121562952	491g0000060.1	1382	-40,5	-2,930535456
492g0000030.1	Hepatocyte growth factor receptor	1343	30	2,233804914	492g0000030.1	1343	-76,5	-5,696202532	492g0000030.1	1343	19,5	1,451973194	492g0000030.1	1343	-41,5	-3,090096798
493g0000020.1	40S ribosomal protein S28	511	39,5	7,729941292	493g0000020.1	511	-7,5	-1,467710372	493g0000020.1	511	14,5	2,837573386	493g0000020.1	511	-23,5	-4,598825832
493g0000040.1	FAD-linked oxidoreductase sorD	1579	29	1,836605447	493g0000040.1	1579	-138	-8,739708676	493g0000040.1	1579	29,5	1,868271058	493g0000040.1	1579	-43	-2,723242559
498g0000090.1	E3 ubiquitin-protein ligase UPL7	4119	47	1,141053654	498g0000090.1	4119	-283	-6,87059966	498g0000090.1	4119	93	2,25782957	498g0000090.1	4119	-64,5	-1,565914057
49g0000090.1	Acetophenone carboxylase gamma subunit	3212	50,5	1,572229141	49g0000090.1	3212	-224,5	-6,989414695	49g0000090.1	3212	55,5	1,727895392	49g0000090.1	3212	-80,5	-2,50622665
4g0000010.1	Unknown protein	1575	54,5	3,46031746	4g0000010.1	1575	-130,5	-8,285714286	4g0000010.1	1575	17,5	1,111111111	4g0000010.1	1575	-49	-3,111111111
4g0000050.1	Unknown protein	1850	47,5	2,567567568	4g0000050.1	1850	-113,5	-6,135135135	4g0000050.1	1850	66,5	3,594594595	4g0000050.1	1850	-59	-3,189189189
4g0000080.1	Unknown protein	1081	29	2,682701203	4g0000080.1	1081	-132	-12,21091582	4g0000080.1	1081	18,5	1,711378353	4g0000080.1	1081	-17	-1,572617946
4g0000250.1	Unknown protein	1294	45	3,477588872	4g0000250.1	1294	-122	-9,42812983	4g0000250.1	1294	17	1,313755796	4g0000250.1	1294	-22,5	-1,738794436
4g0000360.1	Unknown protein	2020	23,5	1,163366337	4g0000360.1	2020	-95	-4,702970297	4g0000360.1	2020	79,5	3,935643564	4g0000360.1	2020	-40	-1,98019802
4g0000420.1	Unknown protein	2581	37,5	1,452925223	4g0000420.1	2581	-139,5	-5,404881829	4g0000420.1	2581	48,5	1,879116622	4g0000420.1	2581	-58,5	-2,266563348
4g0000440.1	Unknown protein	1070	61,5	5,747663551	4g0000440.1	1070	-71	-6,635514019	4g0000440.1	1070	16,5	1,542056075	4g0000440.1	1070	-41	-3,831775701
4g0000490.1	Unknown protein	2913	58	1,991074494	4g0000490.1	2913	-189	-6,48815654	4g0000490.1	2913	52	1,78510127	4g0000490.1	2913	-48,5	-1,664950223
500g0000020.1	Serine/threonine-protein kinase SRPK	1178	47	3,989813243	500g0000020.1	1178	-66	-5,602716469	500g0000020.1	1178	16,5	1,400679117	500g0000020.1	1178	-39,5	-3,353140917
501g0000030.1	Unknown protein	1862	55	2,953813104	501g0000030.1	1862	-147,5	-7,921589689	501g0000030.1	1862	39,5	2,121374866	501g0000030.1	1862	-27	-1,450053706
506g0000060.1	Unknown protein	2077	47,5	2,286952335	506g0000060.1	2077	-118	-5,681271064	506g0000060.1	2077	51,5	2,479537795	506g0000060.1	2077	-77,5	-3,731343284
506g0000090.1	Unknown protein	711	81,5	11,46272855	506g0000090.1	711	-51,5	-7,243319269	506g0000090.1	711	14,5	2,039381153	506g0000090.1	711	-7,5	-1,054852321
507g0000050.1	4,5-DOPA dioxygenase extradiol 1	307	48,5	15,7980456	507g0000050.1	307	-18,5	-6,026058632	507g0000050.1	307	4	1,302931596	507g0000050.1	307	-6,5	-2,117263844
509g0000060.1	Peptide chain release factor 1	1787	18	1,007274762	509g0000060.1	1787	-103	-5,763850028	509g0000060.1	1787	34,5	1,930609961	509g0000060.1	1787	-35	-1,958589815
50g0000080.1	Unknown protein	560	9	1,607142857	50g0000080.1	560	-41	-7,321428571	50g0000080.1	560	15,5	2,767857143	50g0000080.1	560	-28,5	-5,089285714
510g0000020.1	RNA replication protein	1259	31	2,462271644	510g0000020.1	1259	-114	-9,054805401	510g0000020.1	1259	25,5	2,025416998	510g0000020.1	1259	-62,5	-4,964257347
510g0000040.1	Chaperone protein DnaJ	2371	40	1,687051877	510g0000040.1	2371	-165,5	-6,98017714	510g0000040.1	2371	34	1,433994095	510g0000040.1	2371	-74	-3,121045972
512g0000030.1	SH3 domain-containing kinase-binding protein 1	3533	145	4,10416077	512g0000030.1	3533	-263	-7,4440985	512g0000030.1	3533	55,5	1,570902915	512g0000030.1	3533	-81,5	-2,306821398
513g0000060.1	Claspin	1350	19	1,407407407	513g0000060.1	1350	-51,5	-3,814814815	513g0000060.1	1350	45	3,333333333	513g0000060.1	1350	-24	-1,777777778
515g0000070.1	Pre-mRNA-processing protein 45	2038	29,5	1,447497547	515g0000070.1	2038	-122,5	-6,010794897	515g0000070.1	2038	43	2,109911678	515g0000070.1	2038	-47,5	-2,330716389
51g0000050.1	Importin beta-like protein kap111	3927	42,5	1,082251082	51g0000050.1	3927	-255,5	-6,506238859	51g0000050.1	3927	103,5	2,635599694	51g0000050.1	3927	-135	-3,437738732
51g0000100.1	DNA-directed RNA polymerase subunit beta'	293	52,5	17,91808874	51g0000100.1	293	-16	-5,460750853	51g0000100.1	293	5	1,706484642	51g0000100.1	293	-7,5	-2,559726963
51g0000160.1	Holo-[acyl-carrier-protein] synthase	215	7,5	3,488372093	51g0000160.1	215	-16	-7,441860465	51g0000160.1	215	12,5	5,813953488	51g0000160.1	215	-5	-2,325581395
521g0000040.1	Unknown protein	2543	30,5	1,199370822	521g0000040.1	2543	-189	-7,432166732	521g0000040.1	2543	30	1,179709005	521g0000040.1	2543	-69	-2,713330712
527g0000010.1	Unknown protein	2807	57,5	2,048450303	527g0000010.1	2807	-102,5	-3,651585322	527g0000010.1	2807	54	1,923762024	527g0000010.1	2807	-33	-1,175632348
527g0000050.1	Unknown protein	2402	25	1,040799334	527g0000050.1	2402	-174,5	-7,264779351	527g0000050.1	2402	26	1,082431307	527g0000050.1	2402	-48,5	-2,019150708
52g0000050.1	Unknown protein	540	104	19,25925926	52g0000050.1	540	-16,5	-3,055555556	52g0000050.1	540	8	1,481481482	52g0000050.1	540	-16	-2,962962963
52g0000090.1	Unknown protein	1699	68	4,002354326	52g0000090.1	1699	-96,5	-5,679811654	52g0000090.1	1699	18,5	1,088875809	52g0000090.1	1699	-28,5	-1,677457328
52g0000150.1	Unknown protein	1226	20	1,63132137	52g0000150.1	1226	-47	-3,83360522	52g0000150.1	1226	15	1,223491028	52g0000150.1	1226	-30,5	-2,48776509
52g0000240.1	Unknown protein	923	43,5	4,712892741	52g0000240.1	923	-45,5	-4,929577465	52g0000240.1	923	11,5	1,245937161	52g0000240.1	923	-20	-2,166847237
52g0000290.1	Unknown protein	1822	102,5	5,625686059	52g0000290.1	1822	-140	-7,683863886	52g0000290.1	1822	38	2,085620198	52g0000290.1	1822	-30	-1,646542261
530g0000060.1	MOB kinase activator-like 1A	1118	17,5	1,56529517	530g0000060.1	1118	-45	-4,025044723	530g0000060.1	1118	33,5	2,996422183	530g0000060.1	1118	-30	-2,683363149
538g0000050.1	Unknown protein	2053	176	8,572820263	538g0000050.1	2053	-136,5	-6,648806625	538g0000050.1	2053	27,5	1,339503166	538g0000050.1	2053	-26,5	-1,29079396
539g0000010.1	Unknown protein	321	39	12,14953271	539g0000010.1	321	-6	-1,869158879	539g0000010.1	321	10,5	3,271028037	539g0000010.1	321	-6	-1,869158879
539g0000020.1	Unknown protein	545	11,5	2,110091743	539g0000020.1	545	-10	-1,834862385	539g0000020.1	545	19,5	3,577981651	539g0000020.1	545	-26	-4,770642202
53g0000090.1	Cutinase	849	36,5	4,299175501	53g0000090.1	849	-49,5	-5,830388693	53g0000090.1	849	12	1,413427562	53g0000090.1	849	-12,5	-1,472320377
53g0000140.1	F-box/LRR-repeat protein 17	2218	67	3,020739405	53g0000140.1	2218	-141,5	-6,37962128	53g0000140.1	2218	32	1,442741208	53g0000140.1	2218	-80	-3,606853021
53g0000150.1	DNA repair protein RAD16	3492	43	1,231386025	53g0000150.1	3492	-205	-5,870561283	53g0000150.1	3492	83	2,376861398	53g0000150.1	3492	-40,5	-1,159793814
53g0000180.1	Gamma-taxilin	2699	121,5	4,501667284	53g0000180.1	2699	-129	-4,779547981	53g0000180.1	2699	38	1,407928863	53g0000180.1	2699	-32	-1,185624305
53g0000230.1	Ornithine decarboxylase	1367	33,5	2,4506218	53g0000230.1	1367	-74	-5,413313826	53g0000230.1	1367	31,5	2,304316021	53g0000230.1	1367	-16,5	-1,207022677
541g0000030.1	Membrane-associated guanylate kinase, WW and PDZ domain-containir	2358	33,5	1,420695505	541g0000030.1	2358	-192	-8,142493639	541g0000030.1	2358	59	2,502120441	541g0000030.1	2358	-63	-2,671755725
544g0000060.1	Succinate--CoA ligase [ADP-forming] subunit beta, mitochondria	1925	29	1,506493507	544g0000060.1	1925	-123	-6,38961039	544g0000060.1	1925	20,5	1,064935065	544g0000060.1	1925	-20,5	-1,064935065
546g0000020.1	Unknown protein	1755	58,5	3,333333333	546g0000020.1	1755	-134	-7,635327635	546g0000020.1	1755	22	1,253561254	546g0000020.1	1755	-27,5	-1,566951567
548g0000080.1	Zinc finger CCCH domain-containing protein 65	664	40	6,024096386	548g0000080.1	664	-39,5	-5,948795181	548g0000080.1	664	17	2,560240964	548g0000080.1	664	-43,5	-6,551204819
549g0000030.1	Protein-tyrosine kinase 2-beta	809	59,5	7,354758962	549g0000030.1	809	-45,5	-5,624227441	549g0000030.1	809	26	3,213844252	549g0000030.1	809	-13,5	-1,668726823
549g0000050.1	Protoheme IX farnesyltransferase	347	4	1,152737752	549g0000050.1	347	-18	-5,187319885	549g0000050.1	347	9	2,593659942	549g0000050.1	347	-25	-7,204610951
549g0000060.1	Zinc cluster transcription factor CZF1	1340	23,5	1,753731343	549g0000060.1	1340	-84,5	-6,305970149	549g0000060.1	1340	40	2,985074627	549g0000060.1	1340	-15	-1,119402985

54g0000030.1	Mitochondrial sodium/calcium exchanger proteir	3594	42,5	1,182526433	54g0000030.1	3594	-200	-5,564830273	54g0000030.1	3594	73	2,03116305	54g0000030.1	3594	-48	-1,335559265
54g0000180.1	Alpha-ketoglutarate-dependent xanthine dioxygenase xanA	1286	26	2,021772939	54g0000180.1	1286	-142	-11,04199067	54g0000180.1	1286	29	2,255054432	54g0000180.1	1286	-36	-2,799377916
552g0000030.1	Ankyrin repeat protein B18	820	24,5	2,987804878	552g0000030.1	820	-35,5	-4,329268293	552g0000030.1	820	20,5	2,5	552g0000030.1	820	-37,5	-4,573170732
552g0000040.1	Clathrin interactor EPSIN 3	2039	37	1,814615007	552g0000040.1	2039	-100,5	-4,928886709	552g0000040.1	2039	23,5	1,152525748	552g0000040.1	2039	-101,5	-4,977930358
552g0000050.1	HTH-type transcriptional repressor PurR	776	8	1,030927835	552g0000050.1	776	-15	-1,932989691	552g0000050.1	776	8	1,030927835	552g0000050.1	776	-15,5	-1,99742268
558g0000020.1	RNA polymerase II subunit A C-terminal domain phosphatase SSU72	250	93,5	37,4	558g0000020.1	250	-10,5	-4,2	558g0000020.1	250	10	4	558g0000020.1	250	-5,5	-2,2
559g0000060.1	GDP-mannose transporter 1	376	34	9,042553192	559g0000060.1	376	-14,5	-3,856382979	559g0000060.1	376	5,5	1,462765957	559g0000060.1	376	-11,5	-3,058510638
559g0000070.1	Chromatin remodeling protein SHL	2088	33	1,58045977	559g0000070.1	2088	-110	-5,268199234	559g0000070.1	2088	29,5	1,412835249	559g0000070.1	2088	-43,5	-2,083333333
55g0000250.1	Pyrroline-5-carboxylate reductase	1004	16	1,593625498	55g0000250.1	1004	-38	-3,784860558	55g0000250.1	1004	22,5	2,241035857	55g0000250.1	1004	-34,5	-3,43625498
565g0000050.1	DNA mismatch repair protein MutS	6438	74	1,149425287	565g0000050.1	6438	-262,5	-4,077353215	565g0000050.1	6438	105,5	1,638707673	565g0000050.1	6438	-193,5	-3,005591799
565g0000060.1	tRNA (guanine(9)-N1)-methyltransferase	1346	26,5	1,968796434	565g0000060.1	1346	-40	-2,971768202	565g0000060.1	1346	45	3,343239227	565g0000060.1	1346	-14	-1,040118871
566g0000030.1	Succinate--CoA ligase [ADP-forming] subunit beta	2412	37	1,533996683	566g0000030.1	2412	-196,5	-8,146766169	566g0000030.1	2412	24,5	1,015754561	566g0000030.1	2412	-79,5	-3,296019901
567g0000030.1	Unknown protein	2523	29	1,149425287	567g0000030.1	2523	-191	-7,570352755	567g0000030.1	2523	54,5	2,160126833	567g0000030.1	2523	-46	-1,823226318
567g0000050.1	Unknown protein	890	42,5	4,775280899	567g0000050.1	890	-18,5	-2,078651685	567g0000050.1	890	38	4,269662921	567g0000050.1	890	-49,5	-5,561797753
568g0000010.1	Siderophore iron transporter 1	1847	21,5	1,164049811	568g0000010.1	1847	-151	-8,175419599	568g0000010.1	1847	29,5	1,597184624	568g0000010.1	1847	-87	-4,710341094
571g0000010.1	Laminin subunit alpha-2	1241	43	3,464947623	571g0000010.1	1241	-62	-4,995970991	571g0000010.1	1241	37,5	3,021756648	571g0000010.1	1241	-25	-2,014504432
574g0000030.1	Unknown protein	905	27,5	3,038674033	574g0000030.1	905	-59	-6,519337017	574g0000030.1	905	25	2,762430939	574g0000030.1	905	-13	-1,436464088
578g0000080.1	Transcription elongation factor A protein 1	2659	31	1,165851824	578g0000080.1	2659	-141,5	-5,321549455	578g0000080.1	2659	85,5	3,215494547	578g0000080.1	2659	-73,5	-2,764197067
57g0000060.1	Pantothenate synthetase	626	40	6,389776358	57g0000060.1	626	-43	-6,869009585	57g0000060.1	626	9,5	1,517571885	57g0000060.1	626	-17,5	-2,795527157
582g0000080.1	Membrane-bound transcription factor site-1 protease	1285	33	2,568093385	582g0000080.1	1285	-75,5	-5,875486381	582g0000080.1	1285	37	2,879377432	582g0000080.1	1285	-24	-1,86770428
586g0000050.1	Unknown protein	1043	35,5	3,403643337	586g0000050.1	1043	-31,5	-3,020134228	586g0000050.1	1043	21	2,013422819	586g0000050.1	1043	-49,5	-4,745925216
589g0000040.1	MFS transporter prlL	1880	29,5	1,569148936	589g0000040.1	1880	-88,5	-4,707446809	589g0000040.1	1880	34,5	1,835106383	589g0000040.1	1880	-44,5	-2,367021277
589g0000070.1	NADP-dependent 3-hydroxy acid dehydrogenase	1197	26,5	2,213868003	589g0000070.1	1197	-45	-3,759398496	589g0000070.1	1197	25	2,08855472	589g0000070.1	1197	-62	-5,179615706
589g0000080.1	Light-harvesting protein B-870 beta chain	586	40	6,825938567	589g0000080.1	586	-35	-5,972696246	589g0000080.1	586	18	3,071672355	589g0000080.1	586	-6,5	-1,109215017
58g0000050.1	Unknown protein	2528	28,5	1,127373418	58g0000050.1	2528	-126,5	-5,003955696	58g0000050.1	2528	57,5	2,274525317	58g0000050.1	2528	-27	-1,068037975
58g0000140.1	Unknown protein	2511	41	1,632815611	58g0000140.1	2511	-79,5	-3,166069295	58g0000140.1	2511	51	2,031063321	58g0000140.1	2511	-28,5	-1,135005974
58g0000200.1	Unknown protein	224	63,5	28,34821429	58g0000200.1	224	-16	-7,142857143	58g0000200.1	224	5	2,232142857	58g0000200.1	224	-2,5	-1,116071429
599g0000030.1	Succinate dehydrogenase [ubiquinone] cytochrome b small subunit, mit	692	24,5	3,540462428	599g0000030.1	692	-51,5	-7,442196532	599g0000030.1	692	14	2,023121387	599g0000030.1	692	-16	-2,312138728
599g0000080.1	Cation/H(+) symporter 13	600	24	4	599g0000080.1	600	-39	-6,5	599g0000080.1	600	20,5	3,416666667	599g0000080.1	600	-17,5	-2,916666667
59g0000030.1	Pre-glycoprotein polyprotein GP complex	230	60	26,08695652	59g0000030.1	230	-26	-11,30434783	59g0000030.1	230	8,5	3,695652174	59g0000030.1	230	-9,5	-4,130434783
59g0000220.1	Palmitoyltransferase SWF1	1832	45,5	2,483624454	59g0000220.1	1832	-99,5	-5,431222707	59g0000220.1	1832	39,5	2,156113537	59g0000220.1	1832	-55	-3,002183406
59g0000250.1	Adenylyl-sulfate kinase	514	40	7,782101167	59g0000250.1	514	-51	-9,922178988	59g0000250.1	514	8,5	1,653696498	59g0000250.1	514	-10,5	-2,042801556
5g0000350.1	Zinc finger MYND domain-containing protein 19	1088	40,5	3,722426471	5g0000350.1	1088	-150,5	-13,83272059	5g0000350.1	1088	15	1,378676471	5g0000350.1	1088	-83	-7,628676471
5g0000460.1	Voltage-dependent anion-selective channel	1334	20	1,499250375	5g0000460.1	1334	-122	-9,145427286	5g0000460.1	1334	30,5	2,286356822	5g0000460.1	1334	-39,5	-2,96101949
601g0000040.1	Unknown protein	700	111	15,85714286	601g0000040.1	700	-81,5	-11,64285714	601g0000040.1	700	15	2,142857143	601g0000040.1	700	-12	-1,714285714
601g0000070.1	Unknown protein	1204	18,5	1,536544851	601g0000070.1	1204	-65	-5,398671096	601g0000070.1	1204	22	1,827242525	601g0000070.1	1204	-13	-1,079734219
603g0000020.1	Unknown protein	3506	44	1,254991443	603g0000020.1	3506	-219,5	-6,26069595	603g0000020.1	3506	54	1,540216771	603g0000020.1	3506	-79,5	-2,267541358
605g0000040.1	UPF0098 protein PH1269	698	34,5	4,94269341	605g0000040.1	698	-44,5	-6,375358166	605g0000040.1	698	21	3,008595989	605g0000040.1	698	-24	-3,438395416
606g0000020.1	Unknown protein	4223	53,5	1,266871892	606g0000020.1	4223	-245	-5,80156287	606g0000020.1	4223	68,5	1,622069619	606g0000020.1	4223	-71	-1,68126924
60g0000090.1	Unknown protein	1901	74	3,892688059	60g0000090.1	1901	-134,5	-7,075223567	60g0000090.1	1901	35	1,841136244	60g0000090.1	1901	-48,5	-2,551288795
612g0000020.1	Succinyl-diaminopimelate desuccinylase	973	14	1,438848921	612g0000020.1	973	-66,5	-6,834532374	612g0000020.1	973	12	1,233299075	612g0000020.1	973	-38	-3,905447071
613g0000030.1	Unknown protein	1663	32	1,924233313	613g0000030.1	1663	-116	-6,975345761	613g0000030.1	1663	30	1,803968731	613g0000030.1	1663	-29,5	-1,773902586
614g0000050.1	1,4-alpha-glucan-branching enzyme	2066	29	1,403678606	614g0000050.1	2066	-119	-5,759922556	614g0000050.1	2066	24,5	1,185866409	614g0000050.1	2066	-59	-2,855759923
615g0000010.1	Mediator of RNA polymerase II transcription subunit 14	789	77,5	9,822560203	615g0000010.1	789	-8	-1,013941698	615g0000010.1	789	10	1,267427123	615g0000010.1	789	-14,5	-1,837769328
619g0000060.1	Bowman-Birk type proteinase inhibitor	1813	56,5	3,116381688	619g0000060.1	1813	-86,5	-4,771097628	619g0000060.1	1813	39,5	2,178709322	619g0000060.1	1813	-29	-1,599558742
61g0000110.1	Amino-acid permease GAP3	1835	27,5	1,498637602	61g0000110.1	1835	-109	-5,940054496	61g0000110.1	1835	35,5	1,934604905	61g0000110.1	1835	-78	-4,250681199
61g0000150.1	Peroxisomal 2,4-dienoyl-CoA reductase SPS19	3290	53,5	1,626139818	61g0000150.1	3290	-152	-4,62006079	61g0000150.1	3290	109,5	3,328267477	61g0000150.1	3290	-38,5	-1,170212766
622g0000040.1	Proteinaceous RNase P 3	1036	68,5	6,611969112	622g0000040.1	1036	-68,5	-6,611969112	622g0000040.1	1036	28	2,702702703	622g0000040.1	1036	-13	-1,254826255
62g0000010.1	Pectate lyase H	1098	49,5	4,508196721	62g0000010.1	1098	-73,5	-6,693989071	62g0000010.1	1098	27	2,459016393	62g0000010.1	1098	-30	-2,732240437
62g0000060.1	3-(3-hydroxy-phenyl)propionate/3-hydroxycinnamic acid hydroxylase	2805	42,5	1,515151515	62g0000060.1	2805	-171,5	-6,114081996	62g0000060.1	2805	32,5	1,158645276	62g0000060.1	2805	-108	-3,85026738
62g0000080.1	Transcription factor MYB124	398	44	11,05527638	62g0000080.1	398	-46	-11,55778894	62g0000080.1	398	13,5	3,391959799	62g0000080.1	398	-7	-1,75879397
62g0000110.1	Adenylate-forming reductase Nps10	2963	39,5	1,333108336	62g0000110.1	2963	-173,5	-5,855551806	62g0000110.1	2963	47,5	1,603104961	62g0000110.1	2963	-48	-1,61997975
62g0000130.1	Vacuolar basic amino acid transporter 2	1432	37,5	2,618715084	62g0000130.1	1432	-45	-3,142458101	62g0000130.1	1432	25	1,745810056	62g0000130.1	1432	-26	-1,815642458
62g0000170.1	Chorismate synthase	500	15	3	62g0000170.1	500	-54	-10,8	62g0000170.1	500	8,5	1,7	62g0000170.1	500	-26,5	-5,3
630g0000010.1	L-arabinitol 4-dehydrogenase	1663	54,5	3,277209862	630g0000010.1	1663	-129	-7,757065544	630g0000010.1	1663	26,5	1,593505713	630g0000010.1	1663	-30	-1,803968731
633g0000010.1	Unknown protein	2284	65	2,845884413	633g0000010.1	2284	-129,5	-5,669877408	633g0000010.1	2284	31,5	1,37915937	633g0000010.1	2284	-76	-3,327495622
633g0000050.1	Unknown protein	1526	85,5	5,602883355	633g0000050.1	1526	-42,5	-2,785058978	633g0000050.1	1526	60,5	3,964613368	633g0000050.1	1526	-19	-1,24508519
636g0000020.1	GTP cyclohydrolase-2	821	22,5	2,740560292	636g0000020.1	821	-45,5	-5,542021925	636g0000020.1	821	17	2,070645554	636g0000020.1	821	-71,5	-8,708891596
636g0000040.1	Zinc finger protein 398	3497	52,5	1,501286817	636g0000040.1	3497	-175,5	-5,018587361	636g0000040.1	3497	66,5	1,901629969	636g0000040.1	3497	-47	-1,344009151
63g0000040.1	Unknown protein	1782	36,5	2,048260382	63g0000040.1	1782	-103,5	-5,808080808	63g0000040.1	1782	32	1,795735129	63g0000040.1	1782	-50	-2,805836139
63g0000090.1	Unknown protein	891	25,5	2,861952862	63g0000090.1	891	-66	-7,407407407	63g0000090.1	891	24,5	2,749719416	63g0000090.1	891	-13,5	-1,515151515
63g0000100.1	Unknown protein	8742	92	1,052390757	63g0000100.1	8742	-417,5	-4,775795013	63g0000100.1	8742	181	2,070464425	63g0000100.1	8742	-88,5	-1,012354152

640g0000030.1	Unknown protein	2768	28	1,011560694	640g0000030.1	2768	-127	-4,588150289	640g0000030.1	2768	44	1,589595376	640g0000030.1	2768	-50,5	-1,824421965
641g0000050.1	Queuine tRNA-ribosyltransferase	223	29	13,0044843	641g0000050.1	223	-9,5	-4,260089686	641g0000050.1	223	3	1,34529148	641g0000050.1	223	-9	-4,03587444
642g0000020.1	Eukaryotic translation initiation factor 2-alpha kinase gcn-2	297	4	1,346801347	642g0000020.1	297	-38	-12,79461279	642g0000020.1	297	3,5	1,178451179	642g0000020.1	297	-11	-3,703703704
646g0000010.1	Dihydroxyacetone kinase	1836	47,5	2,58714597	646g0000010.1	1836	-128	-6,97167756	646g0000010.1	1836	21	1,14379085	646g0000010.1	1836	-26	-1,416122004
652g0000040.1	Guanine nucleotide-binding protein alpha-5 subunit	3495	51	1,459227468	652g0000040.1	3495	-244,5	-6,995708155	652g0000040.1	3495	76	2,17453505	652g0000040.1	3495	-60	-1,716738197
653g0000020.1	Unknown protein	1305	97	7,432950192	653g0000020.1	1305	-109	-8,352490422	653g0000020.1	1305	21,5	1,647509579	653g0000020.1	1305	-28,5	-2,183908046
657g0000040.1	N-acetyl-D-glucosamine kinase	631	70,5	11,17274168	657g0000040.1	631	-24,5	-3,882725832	657g0000040.1	631	22	3,486529319	657g0000040.1	631	-27,5	-4,358161648
659g0000020.1	Unknown protein	827	50,5	6,106408706	659g0000020.1	827	-27,5	-3,325272068	659g0000020.1	827	13,5	1,632406288	659g0000020.1	827	-10	-1,209189843
65g0000160.1	Unknown protein	3599	68	1,889413726	65g0000160.1	3599	-303,5	-8,432898027	65g0000160.1	3599	49	1,361489303	65g0000160.1	3599	-87,5	-2,431230898
662g0000010.1	Ubiquitin-conjugating enzyme E2 E2	771	35,5	4,604409857	662g0000010.1	771	-28,5	-3,696498055	662g0000010.1	771	22,5	2,918287938	662g0000010.1	771	-19,5	-2,529182879
663g0000010.1	1-deoxy-D-xylulose-5-phosphate synthase	2223	43,5	1,956815115	663g0000010.1	2223	-95,5	-4,295996401	663g0000010.1	2223	26	1,169590643	663g0000010.1	2223	-23	-1,034637877
664g0000030.1	Unknown protein	1196	128	10,70234114	664g0000030.1	1196	-91,5	-7,650501672	664g0000030.1	1196	23,5	1,964882943	664g0000030.1	1196	-15	-1,254180602
665g0000010.1	NAD(+) kinase	1435	15,5	1,080139373	665g0000010.1	1435	-67	-4,668989547	665g0000010.1	1435	19,5	1,358885017	665g0000010.1	1435	-51	-3,554006969
665g0000030.1	2,3-bisphospHOGlycerate-dependent phospHOGlycerate mutase	971	32	3,295571576	665g0000030.1	971	-81	-8,341915551	665g0000030.1	971	15	1,544799176	665g0000030.1	971	-19,5	-2,008238929
66g0000030.1	Asparagine--tRNA ligase	2542	49	1,92761605	66g0000030.1	2542	-119,5	-4,701022817	66g0000030.1	2542	96	3,776553895	66g0000030.1	2542	-52	-2,04563336
66g0000050.1	ATP-sensitive inward rectifier potassium channel 15	2252	227,5	10,10213144	66g0000050.1	2252	-146,5	-6,505328597	66g0000050.1	2252	44	1,953818828	66g0000050.1	2252	-56,5	-2,508880995
66g0000070.1	Tryptophan--tRNA ligase	287	28,5	9,930313589	66g0000070.1	287	-7	-2,43902439	66g0000070.1	287	11	3,832752613	66g0000070.1	287	-8,5	-2,961672474
66g0000110.1	Trichothecene biosynthesis transcription regulator TRI10	1726	23,5	1,361529548	66g0000110.1	1726	-125	-7,242178447	66g0000110.1	1726	43	2,491309386	66g0000110.1	1726	-47,5	-2,75202781
672g0000040.1	Unknown protein	533	10,5	1,969981238	672g0000040.1	533	-12,5	-2,34521576	672g0000040.1	533	9	1,688555347	672g0000040.1	533	-24,5	-4,596622889
672g0000070.1	Unknown protein	1163	137,5	11,82287188	672g0000070.1	1163	-73,5	-6,319862425	672g0000070.1	1163	18,5	1,590713672	672g0000070.1	1163	-17,5	-1,504729149
678g0000020.1	ATP synthase subunit a	1415	32	2,261484099	678g0000020.1	1415	-65,5	-4,628975265	678g0000020.1	1415	32	2,261484099	678g0000020.1	1415	-42	-2,96819788
678g0000040.1	General negative regulator of transcription subunit 4	1473	74	5,023761032	678g0000040.1	1473	-115	-7,807196198	678g0000040.1	1473	37,5	2,545824847	678g0000040.1	1473	-76,5	-5,193482688
67g0000010.1	Unknown protein	2882	77,5	2,689104788	67g0000010.1	2882	-162,5	-5,638445524	67g0000010.1	2882	55	1,908396947	67g0000010.1	2882	-72,5	-2,515614157
67g0000090.1	Unknown protein	1967	56,5	2,872394509	67g0000090.1	1967	-66	-3,355363498	67g0000090.1	1967	46	2,33858668	67g0000090.1	1967	-20,5	-1,042196238
686g0000010.1	Unknown protein	3881	59,5	1,533110023	686g0000010.1	3881	-205	-5,282143777	686g0000010.1	3881	79	2,035557846	686g0000010.1	3881	-39	-1,004895646
688g0000030.1	Trans-enoyl reductase FFUJ_12240	1627	20,5	1,259987707	688g0000030.1	1627	-106,5	-6,545789797	688g0000030.1	1627	46,5	2,858020897	688g0000030.1	1627	-23,5	-1,444376152
68g0000020.1	Outer membrane protein assembly factor BamA	1750	28	1,6	68g0000020.1	1750	-186,5	-10,65714286	68g0000020.1	1750	38	2,171428571	68g0000020.1	1750	-23	-1,314285714
68g0000040.1	Alpha-L-arabinofuranosidase A	3919	81,5	2,079612146	68g0000040.1	3919	-221,5	-5,651952029	68g0000040.1	3919	49,5	1,263077316	68g0000040.1	3919	-65	-1,658586374
68g0000060.1	Inter-alpha-trypsin inhibitor heavy chain H3	3031	46,5	1,534147146	68g0000060.1	3031	-184	-6,070603761	68g0000060.1	3031	32,5	1,072253382	68g0000060.1	3031	-87	-2,870339822
68g0000080.1	MFS transporter prlL	2537	28	1,103665747	68g0000080.1	2537	-135,5	-5,340953883	68g0000080.1	2537	78	3,074497438	68g0000080.1	2537	-48,5	-1,91170674
691g0000030.1	Unknown protein	2081	32	1,537722249	691g0000030.1	2081	-128	-6,150888996	691g0000030.1	2081	39	1,874098991	691g0000030.1	2081	-55,5	-2,666987026
691g0000060.1	Unknown protein	755	30	3,973509934	691g0000060.1	755	-17	-2,251655629	691g0000060.1	755	35	4,635761589	691g0000060.1	755	-39,5	-5,23178808
697g0000010.1	Transcription factor kojR	2639	36	1,364153088	697g0000010.1	2639	-172,5	-6,536566881	697g0000010.1	2639	52	1,97044335	697g0000010.1	2639	-52	-1,97044335
69g0000110.1	Conserved oligomeric Golgi complex subunit 2	893	31	3,471444569	69g0000110.1	893	-81	-9,070548712	69g0000110.1	893	14	1,56774916	69g0000110.1	893	-16	-1,791713326
69g0000170.1	Mitochondrial fusion and transport protein ugo1	1992	23	1,154618474	69g0000170.1	1992	-102,5	-5,145582329	69g0000170.1	1992	47,5	2,384538153	69g0000170.1	1992	-45,5	-2,284136546
69g0000190.1	RRP15-like protein	1016	38,5	3,789370079	69g0000190.1	1016	-61,5	-6,053149606	69g0000190.1	1016	16,5	1,624015748	69g0000190.1	1016	-11	-1,082677165
69g0000230.1	Protein RnfH	1366	30,5	2,232796486	69g0000230.1	1366	-85,5	-6,259150805	69g0000230.1	1366	18,5	1,35431918	69g0000230.1	1366	-32,5	-2,37920937
6g0000110.1	DNA polymerase III PolC-type	1359	18,5	1,36129507	6g0000110.1	1359	-70,5	-5,187637969	6g0000110.1	1359	15,5	1,140544518	6g0000110.1	1359	-38	-2,796173657
6g0000120.1	Nitrate reductase [NADH]	3209	60	1,869741352	6g0000120.1	3209	-145	-4,518541602	6g0000120.1	3209	104	3,240885011	6g0000120.1	3209	-55,5	-1,729510751
6g0000190.1	Zinc-binding protein A33	1574	16	1,016518424	6g0000190.1	1574	-81,5	-5,177890724	6g0000190.1	1574	39,5	2,50952986	6g0000190.1	1574	-33	-2,09656925
6g0000290.1	Signal peptidase complex subunit 3A	1759	28,5	1,620238772	6g0000290.1	1759	-118	-6,708357021	6g0000290.1	1759	32,5	1,847640705	6g0000290.1	1759	-102	-5,798749289
6g0000320.1	D-amino acid dehydrogenase	1130	16,5	1,460176991	6g0000320.1	1130	-51	-4,513274336	6g0000320.1	1130	43	3,805309735	6g0000320.1	1130	-25	-2,212389381
6g0000380.1	Met repressor	1284	23	1,791277259	6g0000380.1	1284	-80,5	-6,269470405	6g0000380.1	1284	20	1,557632399	6g0000380.1	1284	-37,5	-2,920560748
6g0000450.1	Glutamyl-tRNA(Gln) amidotransferase subunit A	3265	39	1,194486983	6g0000450.1	3265	-229,5	-7,029096478	6g0000450.1	3265	42	1,286370597	6g0000450.1	3265	-101	-3,093415008
6g0000470.1	UPF0234 protein YajQ	759	37	4,87483531	6g0000470.1	759	-13,5	-1,778656127	6g0000470.1	759	12	1,581027668	6g0000470.1	759	-11,5	-1,515151515
6g0000550.1	Unknown protein	1461	26,5	1,813826147	6g0000550.1	1461	-80	-5,475701574	6g0000550.1	1461	18,5	1,266255989	6g0000550.1	1461	-57,5	-3,935660507
701g0000050.1	MFS transporter asaE	892	49	5,493273543	701g0000050.1	892	-43	-4,820627803	701g0000050.1	892	16	1,793721973	701g0000050.1	892	-40	-4,484304933
701g0000080.1	Histidinol dehydrogenase	421	14	3,325415677	701g0000080.1	421	-45	-10,6888361	701g0000080.1	421	13	3,087885986	701g0000080.1	421	-15,5	-3,681710214
70g0000040.1	Kinesin-like protein 5	3684	46,5	1,262214984	70g0000040.1	3684	-182	-4,940282302	70g0000040.1	3684	50	1,357220413	70g0000040.1	3684	-89	-2,415852334
70g0000150.1	Mitochondrial import inner membrane translocase subunit TIM50	2048	82	4,00390625	70g0000150.1	2048	-102,5	-5,004882813	70g0000150.1	2048	36,5	1,782226563	70g0000150.1	2048	-21,5	-1,049804688
713g0000010.1	Unknown protein	1709	31,5	1,843183148	713g0000010.1	1709	-80,5	-4,710356934	713g0000010.1	1709	28	1,638385021	713g0000010.1	1709	-57	-3,335283792
714g0000020.1	DNA mismatch repair protein MutS	731	61	8,344733242	714g0000020.1	731	-38,5	-5,266757866	714g0000020.1	731	18	2,462380301	714g0000020.1	731	-40,5	-5,540355677
71g0000120.1	Actin-related protein 2/3 complex subunit 1A	1257	77,5	6,165473349	71g0000120.1	1257	-72,5	-5,767700875	71g0000120.1	1257	33,5	2,665075577	71g0000120.1	1257	-40	-3,182179793
722g0000010.1	Fucose-specific lectin	734	26,5	3,610354223	722g0000010.1	734	-52,5	-7,152588556	722g0000010.1	734	12,5	1,702997275	722g0000010.1	734	-14,5	-1,975476839
731g0000010.1	LIM domain-containing protein 1	2013	39	1,937406855	731g0000010.1	2013	-99	-4,918032787	731g0000010.1	2013	62	3,079980129	731g0000010.1	2013	-42,5	-2,111276701
735g0000070.1	3-isopropylmalate dehydrogenase	1073	19,5	1,817334576	735g0000070.1	1073	-127,5	-11,88257223	735g0000070.1	1073	29,5	2,749301025	735g0000070.1	1073	-45	-4,193849021
73g0000110.1	Unknown protein	1090	32,5	2,981651376	73g0000110.1	1090	-72,5	-6,651376147	73g0000110.1	1090	21,5	1,972477064	73g0000110.1	1090	-13	-1,192660551
73g0000130.1	Unknown protein	1477	22	1,489505755	73g0000130.1	1477	-77	-5,213270142	73g0000130.1	1477	29,5	1,997291808	73g0000130.1	1477	-16	-1,083276913
740g0000030.1	Glutamyl-tRNA(Gln) amidotransferase subunit D	389	37,5	9,640102828	740g0000030.1	389	-25	-6,426735219	740g0000030.1	389	8	2,05655527	740g0000030.1	389	-16	-4,11311054
740g0000040.1	General transcription and DNA repair factor IIH helicase subunit XPB	2450	69	2,816326531	740g0000040.1	2450	-175,5	-7,163265306	740g0000040.1	2450	27	1,102040816	740g0000040.1	2450	-30,5	-1,244897959
740g0000070.1	Phosphoribosylaminoimidazole-succinocarboxamide synthase	639	29	4,538341158	740g0000070.1	639	-32,5	-5,086071988	740g0000070.1	639	17	2,660406886	740g0000070.1	639	-7,5	-1,17370892
744g0000030.1	Ferric/cupric reductase transmembrane component B	2742	38	1,385849745	744g0000030.1	2742	-177	-6,455142232	744g0000030.1	2742	74	2,698760029	744g0000030.1	2742	-104,5	-3,811086798

74g0000090.1	Unknown protein	1787	69	3,861219922	74g0000090.1	1787	-150	-8,393956351	74g0000090.1	1787	23	1,287073307	74g0000090.1	1787	-28	-1,566871852
74g0000120.1	Unknown protein	296	87,5	29,56081081	74g0000120.1	296	-4,5	-1,52027027	74g0000120.1	296	13,5	4,560810811	74g0000120.1	296	-5,5	-1,858108108
74g0000160.1	Unknown protein	1352	42	3,106508876	74g0000160.1	1352	-99,5	-7,359467456	74g0000160.1	1352	16	1,183431953	74g0000160.1	1352	-23,5	-1,738165681
750g0000020.1	Copper transporter 6	664	56,5	8,509036145	750g0000020.1	664	-67	-10,09036145	750g0000020.1	664	25,5	3,840361446	750g0000020.1	664	-9,5	-1,430722892
751g0000040.1	Histone H2B	608	98,5	16,20065789	751g0000040.1	608	-29	-4,769736842	751g0000040.1	608	17	2,796052632	751g0000040.1	608	-14	-2,302631579
758g0000010.1	F-box/LRR-repeat protein 4	2651	42,5	1,603168616	758g0000010.1	2651	-187	-7,053941909	758g0000010.1	2651	37	1,395699736	758g0000010.1	2651	-40,5	-1,527725387
758g0000020.1	Regulator of telomere elongation helicase 1 homolog	340	24,5	7,205882353	758g0000020.1	340	-5,5	-1,617647059	758g0000020.1	340	20	5,882352941	758g0000020.1	340	-19,5	-5,735294118
759g0000010.1	Structure-specific endonuclease subunit SLX1	379	20	5,277044855	759g0000010.1	379	-71	-18,73350923	759g0000010.1	379	11	2,90237467	759g0000010.1	379	-20,5	-5,408970976
760g0000020.1	Unknown protein	3072	51,5	1,676432292	760g0000020.1	3072	-141,5	-4,606119792	760g0000020.1	3072	61	1,985677083	760g0000020.1	3072	-54	-1,7578125
760g0000030.1	Unknown protein	629	45	7,154213037	760g0000030.1	629	-10,5	-1,669316375	760g0000030.1	629	8	1,271860095	760g0000030.1	629	-9	-1,430842607
762g0000010.1	Unknown protein	537	35,5	6,610800745	762g0000010.1	537	-34,5	-6,424581006	762g0000010.1	537	25	4,655493482	762g0000010.1	537	-31	-5,772811918
764g0000020.1	Unknown protein	1586	36,5	2,301387138	764g0000020.1	1586	-111	-6,998738966	764g0000020.1	1586	16,5	1,04035309	764g0000020.1	1586	-47,5	-2,994955864
768g0000030.1	Tubulin-specific chaperone A	1221	31	2,538902539	768g0000030.1	1221	-75	-6,142506143	768g0000030.1	1221	21	1,71990172	768g0000030.1	1221	-24	-1,965601966
76g0000010.1	2,3-bisphospHOGlycerate-dependent phospHOGlycerate mutase	2037	32,5	1,595483554	76g0000010.1	2037	-125,5	-6,16102111	76g0000010.1	2037	42	2,06185567	76g0000010.1	2037	-64,5	-3,166421208
76g0000060.1	Cytoskeleton protein RodZ	1376	48	3,488372093	76g0000060.1	1376	-142	-10,31976744	76g0000060.1	1376	23	1,671511628	76g0000060.1	1376	-14	-1,017441861
76g0000070.1	Immunoglobulin A1 protease autotransporter	2015	23	1,141439206	76g0000070.1	2015	-124,5	-6,17866005	76g0000070.1	2015	28	1,389578164	76g0000070.1	2015	-41	-2,034739454
76g0000080.1	DNA-directed RNA polymerase subunit 2	2624	29,5	1,124237805	76g0000080.1	2624	-177,5	-6,764481707	76g0000080.1	2624	43	1,638719512	76g0000080.1	2624	-62,5	-2,381859756
76g0000090.1	Hexose transporter 2	1795	46,5	2,590529248	76g0000090.1	1795	-132	-7,353760446	76g0000090.1	1795	30,5	1,699164345	76g0000090.1	1795	-32,5	-1,810584958
76g0000120.1	60S ribosomal protein L38	549	33,5	6,102003643	76g0000120.1	549	-17	-3,096539162	76g0000120.1	549	21,5	3,916211293	76g0000120.1	549	-6	-1,092896175
771g0000010.1	Unknown protein	376	8,5	2,260638298	771g0000010.1	376	-23	-6,117021277	771g0000010.1	376	4	1,063829787	771g0000010.1	376	-7	-1,861702128
772g0000030.1	Xanthocillin biosynthesis cluster transcription factor xanC	1430	21,5	1,503496504	772g0000030.1	1430	-85	-5,944055944	772g0000030.1	1430	37	2,587412587	772g0000030.1	1430	-24,5	-1,713286713
774g0000010.1	Retrovirus-related Pol polyprotein from type-1 retrotransposable eleme	2698	101,5	3,76204596	774g0000010.1	2698	-349	-12,93550778	774g0000010.1	2698	150	5,559673833	774g0000010.1	2698	-85	-3,150481838
77g0000010.1	Unknown protein	1475	22	1,491525424	77g0000010.1	1475	-77,5	-5,254237288	77g0000010.1	1475	20,5	1,389830509	77g0000010.1	1475	-18	-1,220338983
77g0000170.1	Unknown protein	1214	14,5	1,194398682	77g0000170.1	1214	-112,5	-9,266886326	77g0000170.1	1214	15	1,235584844	77g0000170.1	1214	-27	-2,224052718
780g0000050.1	Unknown protein	3149	60	1,905366783	780g0000050.1	3149	-225	-7,145125437	780g0000050.1	3149	64	2,032391235	780g0000050.1	3149	-64,5	-2,048269292
78g0000050.1	Carboxy-S-adenosyl-L-methionine synthase	407	9,5	2,334152334	78g0000050.1	407	-16	-3,931203931	78g0000050.1	407	9,5	2,334152334	78g0000050.1	407	-6	-1,474201474
795g0000010.1	Unknown protein	320	9,5	2,96875	795g0000010.1	320	-18,5	-5,78125	795g0000010.1	320	5	1,5625	795g0000010.1	320	-8,5	-2,65625
796g0000020.1	Urease accessory protein UreG	1951	90	4,613018965	796g0000020.1	1951	-138	-7,073295746	796g0000020.1	1951	20,5	1,050743209	796g0000020.1	1951	-63	-3,229113275
797g0000020.1	Unknown protein	3781	45	1,190161333	797g0000020.1	3781	-224,5	-5,93758265	797g0000020.1	3781	42,5	1,124041259	797g0000020.1	3781	-53	-1,40174557
798g0000010.1	Unknown protein	340	119,5	35,14705882	798g0000010.1	340	-22	-6,470588235	798g0000010.1	340	7	2,058823529	798g0000010.1	340	-4,5	-1,323529412
79g0000020.1	Unknown protein	2068	56	2,707930368	79g0000020.1	2068	-120,5	-5,82688588	79g0000020.1	2068	26	1,257253385	79g0000020.1	2068	-38	-1,837524178
79g0000130.1	Unknown protein	988	30,5	3,087044534	79g0000130.1	988	-53	-5,36437247	79g0000130.1	988	23	2,327935223	79g0000130.1	988	-19	-1,923076923
7g0000210.1	Unknown protein	1951	44,5	2,280881599	7g0000210.1	1951	-118,5	-6,073808303	7g0000210.1	1951	55,5	2,844695028	7g0000210.1	1951	-40,5	-2,075858534
7g0000260.1	Unknown protein	357	26,5	7,422969188	7g0000260.1	357	-19,5	-5,462184874	7g0000260.1	357	6	1,680672269	7g0000260.1	357	-17	-4,761904762
808g0000020.1	Unknown protein	581	38	6,540447504	808g0000020.1	581	-32,5	-5,593803787	808g0000020.1	581	15	2,581755594	808g0000020.1	581	-16,5	-2,839931153
80g0000060.1	2-oxoglutarate dehydrogenase E1 component	1047	39	3,724928367	80g0000060.1	1047	-50	-4,775549188	80g0000060.1	1047	30	2,865329513	80g0000060.1	1047	-28,5	-2,722063037
80g0000140.1	Mitochondrial inner membrane magnesium transporter mrs2	431	40,5	9,39675174	80g0000140.1	431	-23,5	-5,452436195	80g0000140.1	431	6	1,392111369	80g0000140.1	431	-34,5	-8,004640371
813g0000030.1	Oligopeptide transporter 1	2868	84	2,928870293	813g0000030.1	2868	-140,5	-4,89888424	813g0000030.1	2868	30,5	1,063458856	813g0000030.1	2868	-44	-1,534170153
815g0000010.1	Zinc transporter ZupT	2146	66	3,075489282	815g0000010.1	2146	-164,5	-7,665424045	815g0000010.1	2146	27	1,258154706	815g0000010.1	2146	-50,5	-2,353215284
819g0000020.1	Unknown protein	1775	47	2,647887324	819g0000020.1	1775	-62	-3,492957747	819g0000020.1	1775	55,5	3,126760563	819g0000020.1	1775	-27	-1,521126761
81g0000160.1	Dihydrolipoyllysine-residue acetyltransferase component of pyruvate de	1501	73,5	4,89673551	81g0000160.1	1501	-98,5	-6,562291806	81g0000160.1	1501	16	1,065956029	81g0000160.1	1501	-46	-3,064623584
827g0000010.1	Unknown protein	4099	61	1,488167846	827g0000010.1	4099	-230,5	-5,623322762	827g0000010.1	4099	116,5	2,842156624	827g0000010.1	4099	-62	-1,51256404
833g0000010.1	Unknown protein	1787	84	4,700615557	833g0000010.1	1787	-93	-5,204252938	833g0000010.1	1787	42	2,350307778	833g0000010.1	1787	-54	-3,021824287
836g0000010.1	Pantothenate synthetase	1949	24,5	1,2570549	836g0000010.1	1949	-73,5	-3,7711647	836g0000010.1	1949	40,5	2,077988712	836g0000010.1	1949	-28,5	-1,462288353
84g0000090.1	Ubiquinone biosynthesis protein coq9, mitochondria	870	24,5	2,816091954	84g0000090.1	870	-61	-7,011494253	84g0000090.1	870	18,5	2,126436782	84g0000090.1	870	-31,5	-3,620689655
857g0000030.1	Glutamate--tRNA ligase	622	100,5	16,15755627	857g0000030.1	622	-13	-2,090032154	857g0000030.1	622	15,5	2,491961415	857g0000030.1	622	-21,5	-3,45659164
85g0000110.1	Unknown protein	2698	44,5	1,649369904	85g0000110.1	2698	-132	-4,892512973	85g0000110.1	2698	59,5	2,205337287	85g0000110.1	2698	-32,5	-1,204595997
85g0000130.1	Unknown protein	1137	14	1,231310466	85g0000130.1	1137	-61,5	-5,408970976	85g0000130.1	1137	27,5	2,418645559	85g0000130.1	1137	-11,5	-1,011433597
860g0000010.1	Resistance to glucose repression protein 1	4504	78	1,731793961	860g0000010.1	4504	-143	-3,174955595	860g0000010.1	4504	50,5	1,121225577	860g0000010.1	4504	-65	-1,443161634
862g0000020.1	Isoflavone reductase	1260	15,5	1,23015873	862g0000020.1	1260	-98,5	-7,817460318	862g0000020.1	1260	20,5	1,626984127	862g0000020.1	1260	-47,5	-3,76984127
862g0000030.1	DNA-directed RNA polymerase I subunit RPA43	1460	48	3,287671233	862g0000030.1	1460	-76,5	-5,239726027	862g0000030.1	1460	36,5	2,5	862g0000030.1	1460	-38	-2,602739726
86g0000090.1	Proteasome subunit alpha type-2	1056	21	1,988636364	86g0000090.1	1056	-76,5	-7,244318182	86g0000090.1	1056	12,5	1,183712121	86g0000090.1	1056	-39	-3,693181818
86g0000150.1	Anaerobic nitric oxide reductase flavorubredoxin	1933	59	3,052250388	86g0000150.1	1933	-115,5	-5,975168132	86g0000150.1	1933	39	2,01758924	86g0000150.1	1933	-26,5	-1,370926022
86g0000190.1	Sporulation protein kinase pit1	4610	50	1,084598699	86g0000190.1	4610	-272,5	-5,911062907	86g0000190.1	4610	87,5	1,898047722	86g0000190.1	4610	-81	-1,757049892
87g0000040.1	Pyruvate kinase 1	195	20,5	10,51282051	87g0000040.1	195	-19	-9,743589744	87g0000040.1	195	5	2,564102564	87g0000040.1	195	-13	-6,666666667
891g0000020.1	Unknown protein	1091	28,5	2,61228231	891g0000020.1	1091	-76,5	-7,011915674	891g0000020.1	1091	25,5	2,337305225	891g0000020.1	1091	-35,5	-3,253895509
891g0000040.1	Unknown protein	1390	43	3,09352518	891g0000040.1	1390	-93,5	-6,726618705	891g0000040.1	1390	28	2,014388489	891g0000040.1	1390	-57,5	-4,136690648
89g0000190.1	Unknown protein	1657	63	3,802051901	89g0000190.1	1657	-65,5	-3,952926977	89g0000190.1	1657	51,5	3,108026554	89g0000190.1	1657	-31,5	-1,901025951
89g0000200.1	Unknown protein	2750	46	1,672727273	89g0000200.1	2750	-127,5	-4,636363636	89g0000200.1	2750	57,5	2,090909091	89g0000200.1	2750	-38	-1,381818182
89g0000220.1	Unknown protein	788	15	1,9035533	89g0000220.1	788	-37,5	-4,758883249	89g0000220.1	788	13,5	1,71319797	89g0000220.1	788	-14,5	-1,840101523
89g0000260.1	Unknown protein	477	13,5	2,830188679	89g0000260.1	477	-32,5	-6,813417191	89g0000260.1	477	10,5	2,201257862	89g0000260.1	477	-8,5	-1,78197065
8g0000020.1	HECT-type ubiquitin ligase-interacting protein creD	1644	55	3,345498784	8g0000020.1	1644	-101,5	-6,173965937	8g0000020.1	1644	57,5	3,49756691	8g0000020.1	1644	-34,5	-2,098540146

8g0000050.1	5'-AMP-activated protein kinase subunit gamma	2194	26,5	1,207839562	8g0000050.1	2194	-67	-3,053783045	8g0000050.1	2194	59	2,689152233	8g0000050.1	2194	-77,5	-3,532360985
8g0000180.1	Cutinase	967	63	6,514994829	8g0000180.1	967	-32	-3,309203723	8g0000180.1	967	10	1,034126163	8g0000180.1	967	-17,5	-1,809720786
8g0000310.1	Nicotianamine synthase	1309	27	2,062643239	8g0000310.1	1309	-61	-4,660045837	8g0000310.1	1309	16,5	1,260504202	8g0000310.1	1309	-24,5	-1,871657754
8g0000370.1	Ribosomal RNA large subunit methyltransferase E	466	22,5	4,82832618	8g0000370.1	466	-7	-1,502145923	8g0000370.1	466	11	2,360515022	8g0000370.1	466	-24,5	-5,25751073
8g0000420.1	ATP synthase subunit alpha	509	23	4,518664047	8g0000420.1	509	-33	-6,483300589	8g0000420.1	509	9,5	1,866404715	8g0000420.1	509	-13	-2,554027505
904g0000030.1	CAP-Gly domain-containing linker protein 4	847	10,5	1,239669422	904g0000030.1	847	-27,5	-3,246753247	904g0000030.1	847	46,5	5,489964581	904g0000030.1	847	-23	-2,715466352
918g0000010.1	Transcriptional regulator of yeast form adherence 3	1982	41	2,068617558	918g0000010.1	1982	-53	-2,674066599	918g0000010.1	1982	66	3,329969728	918g0000010.1	1982	-25,5	-1,286579213
91g0000070.1	WD repeat-containing protein 1	3284	43,5	1,324604141	91g0000070.1	3284	-171,5	-5,22228989	91g0000070.1	3284	84,5	2,573081608	91g0000070.1	3284	-89	-2,710109622
925g0000010.1	Meiotically up-regulated gene 9 protein	1651	40	2,422774076	925g0000010.1	1651	-72,5	-4,391278013	925g0000010.1	1651	43,5	2,634766808	925g0000010.1	1651	-38,5	-2,331920049
92g0000170.1	Unknown protein	786	27,5	3,498727735	92g0000170.1	786	-25	-3,180661578	92g0000170.1	786	34,5	4,389312977	92g0000170.1	786	-45	-5,72519084
932g0000010.1	Solute carrier family 2, facilitated glucose transporter member 2	1952	37,5	1,921106557	932g0000010.1	1952	-73	-3,739754098	932g0000010.1	1952	38	1,946721312	932g0000010.1	1952	-28,5	-1,460040984
944g0000020.1	Protein VTS1	2006	23,5	1,171485543	944g0000020.1	2006	-128	-6,380857428	944g0000020.1	2006	25,5	1,271186441	944g0000020.1	2006	-69,5	-3,464606182
957g0000030.1	Esterase	1882	98	5,207226355	957g0000030.1	1882	-98,5	-5,233793836	957g0000030.1	1882	41,5	2,205100956	957g0000030.1	1882	-26,5	-1,408076514
95g0000040.1	50S ribosomal protein L21	932	40	4,291845494	95g0000040.1	932	-36,5	-3,916309013	95g0000040.1	932	12,5	1,341201717	95g0000040.1	932	-26	-2,789699571
95g0000170.1	1D-myo-inositol 2-acetamido-2-deoxy-alpha-D-glucopyranoside deacety	450	15,5	3,444444444	95g0000170.1	450	-15,5	-3,444444444	95g0000170.1	450	21,5	4,777777778	95g0000170.1	450	-6	-1,333333333
95g0000230.1	ATP-dependent RNA helicase DHX8	2791	52,5	1,88104622	95g0000230.1	2791	-125,5	-4,496596202	95g0000230.1	2791	69	2,472232175	95g0000230.1	2791	-64	-2,293084916
965g0000020.1	Unknown protein	789	85	10,77313055	965g0000020.1	789	-51	-6,463878327	965g0000020.1	789	39,5	5,006337136	965g0000020.1	789	-38,5	-4,879594423
97g0000010.1	Nucleoporin GLE1	1709	24,5	1,433586893	97g0000010.1	1709	-122,5	-7,167934465	97g0000010.1	1709	39,5	2,311293154	97g0000010.1	1709	-30,5	-1,784669397
97g0000090.1	Unconventional myosin-XV	1274	33	2,590266876	97g0000090.1	1274	-93	-7,299843014	97g0000090.1	1274	27	2,119309262	97g0000090.1	1274	-16	-1,25588697
97g0000140.1	DNA-directed RNA polymerase subunit beta	334	55,5	16,61676647	97g0000140.1	334	-16,5	-4,940119761	97g0000140.1	334	12,5	3,74251497	97g0000140.1	334	-24	-7,185628743
97g0000160.1	U3 small nucleolar RNA-associated protein 13	2824	38	1,345609065	97g0000160.1	2824	-209	-7,400849858	97g0000160.1	2824	106,5	3,771246459	97g0000160.1	2824	-63,5	-2,248583569
987g0000030.1	Unknown protein	1220	14,5	1,18852459	987g0000030.1	1220	-57,5	-4,713114754	987g0000030.1	1220	39	3,196721312	987g0000030.1	1220	-13	-1,065573771
996g0000010.1	Unknown protein	952	82,5	8,665966387	996g0000010.1	952	-42	-4,411764706	996g0000010.1	952	41,5	4,359243698	996g0000010.1	952	-30,5	-3,203781513
99g0000050.1	Unknown protein	1715	22,5	1,311953353	99g0000050.1	1715	-107,5	-6,268221574	99g0000050.1	1715	46	2,682215743	99g0000050.1	1715	-23,5	-1,370262391
99g0000140.1	Unknown protein	475	56,5	11,89473684	99g0000140.1	475	-14,5	-3,052631579	99g0000140.1	475	20	4,210526316	99g0000140.1	475	-6	-1,263157895
9g0000220.1	Unknown protein	2602	41,5	1,594926979	9g0000220.1	2602	-79	-3,036126057	9g0000220.1	2602	51,5	1,979246733	9g0000220.1	2602	-36	-1,383551115
9g0000240.1	Unknown protein	764	35,5	4,646596859	9g0000240.1	764	-41	-5,366492147	9g0000240.1	764	25,5	3,337696335	9g0000240.1	764	-26,5	-3,468586387

Table S2

937-HP_NODE_NU	GENE NAME	GENE LENGHT	DIFF	%	937-138_NODE_N	GENE LENGHT	DIFF	%	937-150_NODE_I	GENE LENGHT	DIFF	%	937-403_NODE_NU	GENE LENGHT	DIFF	%
116g0000080.1	MFS transporter prlL	1758	-9	-0,51194539	116g0000080.1	1758	-59,5	-3,38452787	116g0000080.1	1758	17,5	0,99544937	116g0000080.1	1758	-26,5	-1,50739477
134g0000120.1	MFS transporter asaE	978	44	4,49897751	134g0000120.1	978	-74	-7,56646217	134g0000120.1	978	7	0,71574642	134g0000120.1	978	-45,5	-4,65235174
14g0000130.1	MFS transporter prlL	1960	-47	-2,39795918	14g0000130.1	1960	-136,5	-6,96428571	14g0000130.1	1960	39,5	2,01530612	14g0000130.1	1960	-18	-0,91836735
15g0000330.1	MFS transporter prlL	2018	5,5	0,27254708	15g0000330.1	2018	-112	-5,55004955	15g0000330.1	2018	0	0	15g0000330.1	2018	-77,5	-3,84043608
16g0000040.1	MFS transporter prlL	2252	10	0,44404973	16g0000040.1	2252	-147,5	-6,54973357	16g0000040.1	2252	47	2,08703375	16g0000040.1	2252	18,5	0,82149201
175g0000010.1	MFS transporter prlL	1304	44,5	3,41257669	175g0000010.1	1304	-40	-3,06748466	175g0000010.1	1304	42,5	3,25920245	175g0000010.1	1304	19	1,45705521
17g0000170.1	MFS transporter prlL	2282	16	0,70113935	17g0000170.1	2282	-143	-6,26643295	17g0000170.1	2282	57,5	2,51971954	17g0000170.1	2282	-15	-0,65731814
252g0000110.1	MFS transporter prlL	1967	5	0,2541942	252g0000110.1	1967	-150,5	-7,65124555	252g0000110.1	1967	19	0,96593798	252g0000110.1	1967	-39,5	-2,00813421
28g0000160.1	MFS transporter prlL	2007	-6,5	-0,32386647	28g0000160.1	2007	-120	-5,97907324	28g0000160.1	2007	0	0	28g0000160.1	2007	-76,5	-3,81165919
32g0000160.1	MFS transporter asaE	1572	34	2,16284987	32g0000160.1	1572	-106,5	-6,77480916	32g0000160.1	1572	52	3,30788804	32g0000160.1	1572	15	0,95419847
358g0000060.1	MFS transporter prlL	1756	8	0,45558087	358g0000060.1	1756	-120,5	-6,86218679	358g0000060.1	1756	2,5	0,14236902	358g0000060.1	1756	-37,5	-2,13553531
35g0000190.1	MFS transporter prlL	1966	51,5	2,61953204	35g0000190.1	1966	-157	-7,98575788	35g0000190.1	1966	37	1,8819939	35g0000190.1	1966	-39,5	-2,00915565
363g0000020.1	MFS transporter prlL	1819	30,5	1,67674546	363g0000020.1	1819	-43,5	-2,39142386	363g0000020.1	1819	59,5	3,27102804	363g0000020.1	1819	-35	-1,92413414
3g0000380.1	MFS transporter prlL	1842	-93	-5,04885993	3g0000380.1	1842	-111,5	-6,05320304	3g0000380.1	1842	27	1,46579805	3g0000380.1	1842	-29,5	-1,60152009
427g0000080.1	MFS transporter asaE	1859	-11	-0,59171598	427g0000080.1	1859	-113,5	-6,10543303	427g0000080.1	1859	34	1,82894029	427g0000080.1	1859	-24	-1,29101668
447g0000110.1	MFS transporter prlL	2024	97,5	4,81719368	447g0000110.1	2024	-149,5	-7,38636364	447g0000110.1	2024	22,5	1,11166008	447g0000110.1	2024	-30,5	-1,506917
49g0000180.1	MFS transporter prlL	3895	9,5	0,24390244	49g0000180.1	3895	-268,5	-6,89345315	49g0000180.1	3895	65,5	1,68164313	49g0000180.1	3895	-66	-1,6944801
504g0000040.1	MFS transporter PfmaC	3535	13	0,36775106	504g0000040.1	3535	-179	-5,06364922	504g0000040.1	3535	40	1,13154173	504g0000040.1	3535	-123	-3,47949081
557g0000020.1	MFS transporter prlL	1681	92	5,47293278	557g0000020.1	1681	-129,5	-7,70374777	557g0000020.1	1681	15	0,892326	557g0000020.1	1681	-67,5	-4,01546698
589g0000040.1	MFS transporter prlL	1880	29,5	1,56914894	589g0000040.1	1880	-88,5	-4,70744681	589g0000040.1	1880	34,5	1,83510638	589g0000040.1	1880	-44,5	-2,36702128
611g0000040.1	MFS transporter prlL	1922	13,5	0,70239334	611g0000040.1	1922	-65	-3,38189386	611g0000040.1	1922	95,5	4,96878252	611g0000040.1	1922	-6	-0,31217482
629g0000050.1	MFS transporter cpaT	2420	91,5	3,78099174	629g0000050.1	2420	-152,5	-6,30165289	629g0000050.1	2420	39,5	1,63223141	629g0000050.1	2420	-11	-0,45454545
662g0000040.1	MFS transporter OpS2	2414	-33	-1,36702568	662g0000040.1	2414	-115	-4,76387738	662g0000040.1	2414	77,5	3,21043911	662g0000040.1	2414	-4	-0,16570008
675g0000040.1	MFS transporter prlL	1953	12,5	0,64004096	675g0000040.1	1953	-69	-3,53302611	675g0000040.1	1953	36,5	1,86891961	675g0000040.1	1953	-28	-1,43369176
68g0000080.1	MFS transporter prlL	2537	28	1,10366575	68g0000080.1	2537	-135,5	-5,34095388	68g0000080.1	2537	78	3,07449744	68g0000080.1	2537	-48,5	-1,91170674
6g0000220.1	MFS transporter prlL	1856	-4	-0,21551724	6g0000220.1	1856	-160	-8,62068966	6g0000220.1	1856	-12	-0,64655172	6g0000220.1	1856	-102	-5,49568966
701g0000050.1	MFS transporter asaE	892	49	5,49327354	701g0000050.1	892	-43	-4,8206278	701g0000050.1	892	16	1,79372197	701g0000050.1	892	-40	-4,48430493
701g0000060.1	MFS transporter asaE	579	-5,5	-0,94991364	701g0000060.1	579	-42,5	-7,3402418	701g0000060.1	579	-23,5	-4,05872193	701g0000060.1	579	-38	-6,56303972
71g0000010.1	MFS transporter prlL	1695	-19,5	-1,15044248	71g0000010.1	1695	-98	-5,78171091	71g0000010.1	1695	62,5	3,68731563	71g0000010.1	1695	-9,5	-0,56047198
758g0000050.1	MFS transporter prlL	2492	-10,5	-0,42134831	758g0000050.1	2492	-149	-5,97913323	758g0000050.1	2492	34,5	1,38443018	758g0000050.1	2492	-48,5	-1,94622793
8g0000190.1	MFS transporter prlL	1655	136	8,21752266	8g0000190.1	1655	-92	-5,55891239	8g0000190.1	1655	14	0,84592145	8g0000190.1	1655	-27	-1,63141994
97g0000080.1	MFS transporter prlL	933	26	2,78670954	97g0000080.1	933	-45,5	-4,87674169	97g0000080.1	933	3	0,32154341	97g0000080.1	933	-4	-0,42872454

937-HP_NODE_NU	GENE NAME	GENE LENGHT	DIFF	%	937-138_NODE_N	GENE LENGHT	DIFF	%	937-150_NODE_I	GENE LENGHT	DIFF	%	937-403_NODE_NU	GENE LENGHT	DIFF	%
299g0000020.1	ABC multidrug transporter mdr1	4702	-4,5	-0,09570396	299g0000020.1	4702	-269	-5,7209698	299g0000020.1	4702	47,5	1,01020842	299g0000020.1	4702	-131	-2,78604849
118g0000070.1	ABC multidrug transporter mdr2	13304	-67,5	-0,50736621	118g0000070.1	13304	-762,5	-5,73135899	118g0000070.1	13304	162	1,21767889	118g0000070.1	13304	-125,5	-0,94332532
281g0000090.1	ABC transporter ATP-binding protein ModF	1865	5	0,26809651	281g0000090.1	1865	-131,5	-7,05093834	281g0000090.1	1865	27	1,44772118	281g0000090.1	1865	5,5	0,29490617
211g0000090.1	ABC transporter ATP-binding protein/permease PDR18	5595	6,5	0,11617516	211g0000090.1	5595	-375	-6,70241287	211g0000090.1	5595	66	1,17962466	211g0000090.1	5595	-116,5	-2,08221626
3g0000440.1	ABC transporter ATP-binding protein/permease PDR18	4603	-12,5	-0,27156202	3g0000440.1	4603	-306	-6,64783837	3g0000440.1	4603	40,5	0,87986096	3g0000440.1	4603	-51	-1,10797306
49g0000220.1	ABC transporter ATP-binding protein/permease PDR18	3818	-0,5	-0,01309586	49g0000220.1	3818	-216,5	-5,67050812	49g0000220.1	3818	108	2,82870613	49g0000220.1	3818	-72,5	-1,89889995
636g0000030.1	ABC transporter ATP-binding protein/permease PDR18	4377	-50	-1,14233493	636g0000030.1	4377	-207,5	-4,74068997	636g0000030.1	4377	133,5	3,05003427	636g0000030.1	4377	12,5	0,28558373
68g0000030.1	ABC transporter ATP-binding protein/permease PDR18	4642	8	0,17233951	68g0000030.1	4642	-256,5	-5,5256355	68g0000030.1	4642	162,5	3,50064627	68g0000030.1	4642	-46	-0,99095218
727g0000030.1	ABC transporter ATP-binding protein/permease PDR18	4775	15,5	0,32460733	727g0000030.1	4775	-362,5	-7,59162304	727g0000030.1	4775	200,5	4,19895288	727g0000030.1	4775	-50	-1,04712042
75g0000090.1	ABC transporter ATP-binding protein/permease PDR18	4832	-42,5	-0,87955298	75g0000090.1	4832	-323	-6,68460265	75g0000090.1	4832	21	0,43460265	75g0000090.1	4832	-90	-1,86258278
75g0000180.1	ABC transporter ATP-binding protein/permease PDR18	4926	-1	-0,02030045	75g0000180.1	4926	-299	-6,06983354	75g0000180.1	4926	96,5	1,9589931	75g0000180.1	4926	-50,5	-1,02517255
10g0000140.1	ABC transporter ATP-binding protein/permease VMR1	4551	-5,5	-0,12085256	10g0000140.1	4551	-207,5	-4,55943749	10g0000140.1	4551	98,5	2,16435948	10g0000140.1	4551	-63	-1,38431114
17g0000190.1	ABC transporter ATP-binding protein/permease VMR1	4711	2,5	0,05306729	17g0000190.1	4711	-302,5	-6,42114201	17g0000190.1	4711	1	0,02122692	17g0000190.1	4711	-165,5	-3,51305455
39g0000150.1	ABC transporter ATP-binding protein/permease VMR1	5084	13	0,25570417	39g0000150.1	5084	-204,5	-4,02242329	39g0000150.1	5084	71	1,39653816	39g0000150.1	5084	-44,5	-0,87529504
496g0000050.1	ABC transporter BEA3	4848	-4,5	-0,09282178	496g0000050.1	4848	-320	-6,60066007	496g0000050.1	4848	74,5	1,53671617	496g0000050.1	4848	-46,5	-0,95915842
32g0000210.1	ABC transporter F family member 5	274	15,5	5,65693431	32g0000210.1	274	-28,5	-10,4014599	32g0000210.1	274	8,5	3,10218978	32g0000210.1	274	-1,5	-0,54744526
239g0000080.1	ABC transporter G family member 11	5811	174,5	3,00292549	239g0000080.1	5811	-448	-7,70951643	239g0000080.1	5811	69,5	1,19600757	239g0000080.1	5811	-110	-1,89296162
28g0000230.1	ABC transporter G family member 11	2100	-19	-0,9047619	28g0000230.1	2100	-89	-4,23809524	28g0000230.1	2100	40	1,9047619	28g0000230.1	2100	-44,5	-2,11904762
238g0000120.1	ABC transporter G family member 2	6568	2,5	0,03806334	238g0000120.1	6568	-435,5	-6,63063337	238g0000120.1	6568	77	1,17235079	238g0000120.1	6568	-159,5	-2,42844093
179g0000070.1	ABC transporter G family member ARB_01379	3448	-18	-0,52204176	179g0000070.1	3448	-274	-7,94663573	179g0000070.1	3448	111,5	3,2337587	179g0000070.1	3448	-59,5	-1,72563805
5g0000410.1	ABC1 family protein MCP2 homolog	2189	-16	-0,73092736	5g0000410.1	2189	-101,5	-4,63682047	5g0000410.1	2189	88	4,0201005	5g0000410.1	2189	-24,5	-1,11923253
32g0000240.1	ABC1 family protein YPL109C, mitochondrial	2183	14	0,64131929	32g0000240.1	2183	-168	-7,69583142	32g0000240.1	2183	37	1,69491525	32g0000240.1	2183	-46,5	-2,1300962

Figure S1 Mapping efficiency of bisulfite treated PE reads with its own genome.

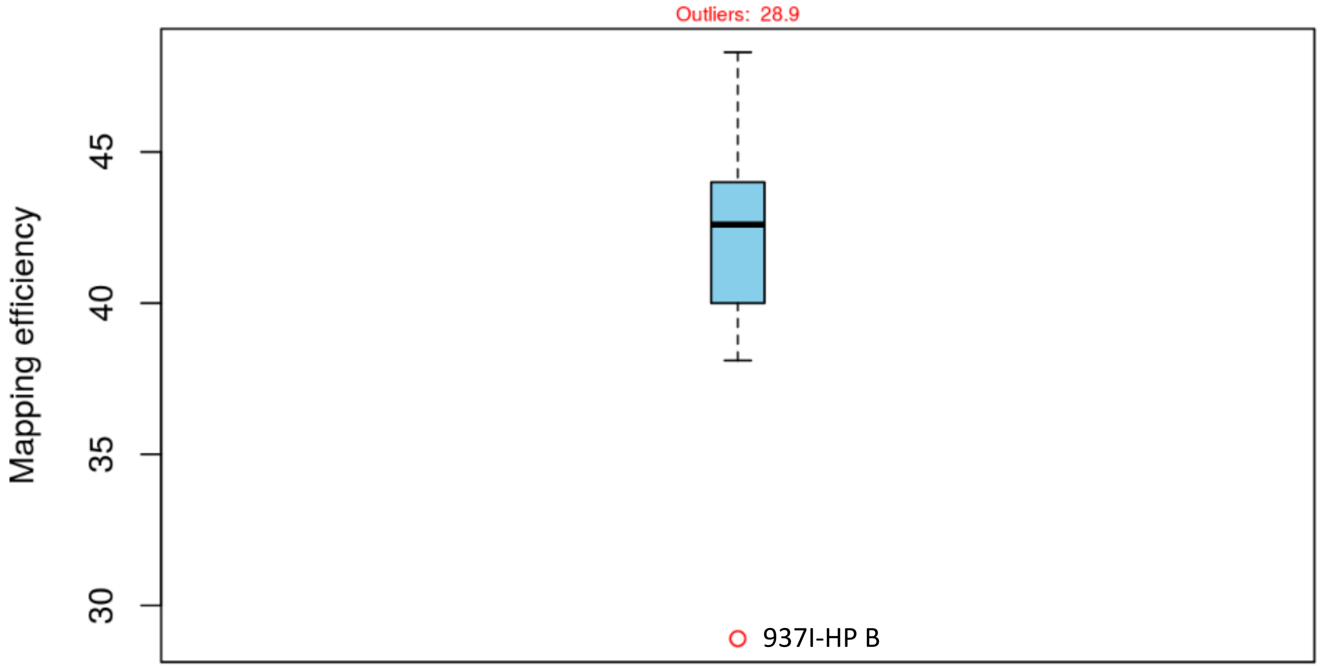

Figure S2 GO term analysis of the 831 genes selected.

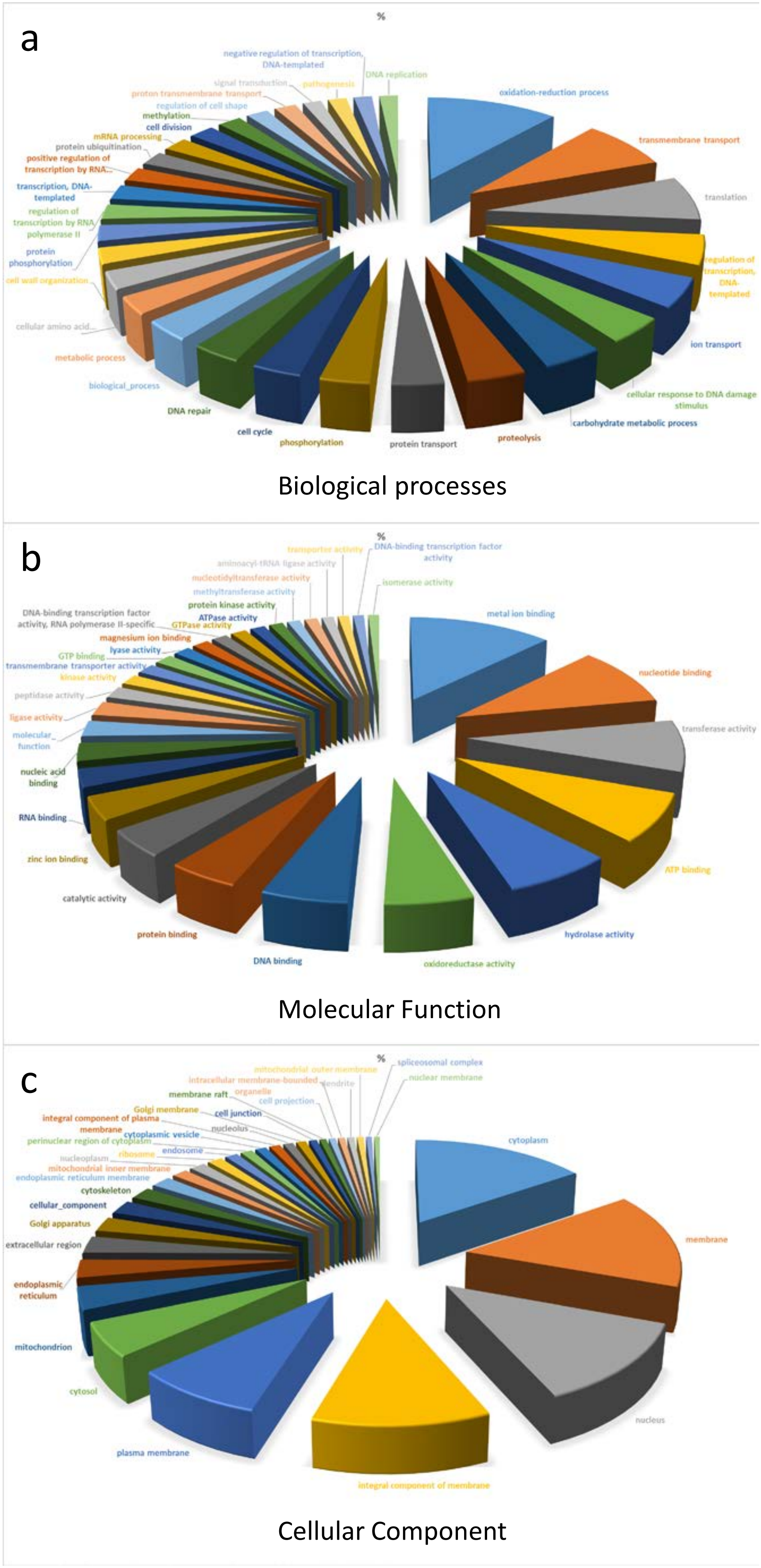

Supplement: Supplementary file 1 [file ijms-21-05197-s001.pdf]
